# Tandocyclinones A and B, Ether Bridged *C*-Glycosyl Benz[*a*]anthracenes from an Intertidal Zone *Streptomyces* sp.

**DOI:** 10.3390/md21090500

**Published:** 2023-09-21

**Authors:** Thanh-Hau Huynh, Eun Seo Bae, Bo Eun Heo, Jayho Lee, Joon Soo An, Yun Kwon, Sang-Jip Nam, Ki-Bong Oh, Jichan Jang, Sang Kook Lee, Dong-Chan Oh

**Affiliations:** 1 Natural Products Research Institute, College of Pharmacy, Seoul National University, Seoul 08826, Republic of Korea; 2019-22632@snu.ac.kr (T.-H.H.); ddol1289@snu.ac.kr (E.S.B.); ahnjunsoo@snu.ac.kr (J.S.A.); sklee61@snu.ac.kr (S.K.L.); 2 Division of Life Science, Department of Bio & Medical Big Data (BK21 Four Program), Research Institute of Life Science, Gyeongsang National University, Jinju 52828, Republic of Korea; hbo0113@naver.com (B.E.H.); jichanjang@gnu.ac.kr (J.J.); 3 Department of Agricultural Biotechnology, College of Agriculture and Life Sciences and Natural Products Research Institute, Seoul National University, Seoul 08826, Republic of Korea; jayho@snu.ac.kr (J.L.); ohkibong@snu.ac.kr (K.-B.O.); 4 Research Institute of Pharmaceutical Sciences, College of Pharmacy, Kyungpook National University, Daegu 41566, Republic of Korea; yunkwon@knu.ac.kr; 5 Department of Chemistry and Nanoscience, Ewha Womans University, Seoul 03760, Republic of Korea; sjnam@ewha.ac.kr

**Keywords:** structure elucidation, *Streptomyces* sp., Crews’ rule, Mosher’s method, Snatzke’s method, *Mycobacterium avium*, antiproliferative activity

## Abstract

Two new proton-deficient metabolites, tandocyclinones A and B (**1** and **2**), were discovered via the chemical profiling of the *Streptomyces* sp. strain TDH03, which was isolated from a marine sediment sample collected from the intertidal mudflat in Tando Port, the Republic of Korea. The structures of **1** and **2** were elucidated as new ether-bridged *C*-glycosyl benz[*a*]anthracenes by using a combination of spectroscopic analyses of ultraviolet (UV) and mass spectrometry (MS) data, along with nuclear magnetic resonance (NMR) spectra, which were acquired in tetrahydrofuran (THF)-*d*_8_ selected after an extensive search for a solvent, resulting in mostly observable exchangeable protons in the ^1^H NMR spectrum. Their configurations were successfully assigned by applying a *J*-based configuration analysis, rotating-frame Overhauser enhancement spectroscopy (ROESY) NMR correlations, chemical derivatization methods based on NMR (a modified version of Mosher’s method) and circular dichroism (CD) (Snatzke’s method using Mo_2_(OAc)_4_-induced CD), as well as quantum-mechanics-based computational methods, to calculate the electronic circular dichroism (ECD). Tandocyclinones A and B (**1** and **2**) were found to have weak antifungal activity against *Trichophyton mentagrophytes* IFM40996 with an MIC value of 128 μg/mL (244 and 265 μM for **1** and **2**, respectively). A further biological evaluation revealed that tandocyclinone A (**1**) displayed inhibitory activity against *Mycobacterium avium* (MIC_50_ = 40.8 μM) and antiproliferative activity against SNU638 and HCT116 cancer cells, with IC_50_ values of 31.9 µM and 49.4 µM, respectively.

## 1. Introduction

The structural determination of natural products requires an integral analysis of the spectroscopic data and significantly depends on the nuclear magnetic resonance (NMR) spectroscopic analysis [1]. However, because the 2D NMR techniques currently utilized in structural analyses are mainly proton-based experiments, the structure elucidation of proton-deficient natural products has been considered a challenging task. In particular, the heuristic “Crews’ rule”, which states that molecules with a ^1^H-to-heteroatom ratio below one may have structures that are difficult to determine via NMR analysis, represents the difficulty in the structure elucidation of proton-deficient natural products [2,3]. Developing and utilizing specialized 2D heteronuclear NMR experiments [4,5,6,7,8] or modern ADEQUATE techniques [9,10] in combination with conventional ^1^H–^13^C techniques (HSQC and HMBC) could allow for a more confident structure determination for proton-deficient molecules. For example, a SEA-XLOC pulse sequence was recently introduced to distinguish between two- and three-bond proton–carbon correlations, especially those involving quaternary carbons, when unambiguous identifications are required [7]. A combination of inverted ^1^*J*_CC_ 1,*n*-ADEQUATE with 1.7 mm cryoprobe technology, using highly proton-deficient alkaloid staurosporine as a case study, is another example [11]. However, such experiments either often require high-quality NMR probes or are only applicable to specific cases, limiting their general application.

The important role of marine natural products in drug discovery from marine microorganisms, algae, sponges, bryozoans, mollusks, etc., has been well-recognized and highlighted in various documents [12,13,14]. Among the sources of marine-derived natural products, marine-derived actinomycetes have long been prolific chemical synthesizers, providing a great variety of biologically active molecules with unprecedented skeletal structures [15]. When structurally novel compounds possess proton-deficient structures, the elucidation of their structures is more challenging than the analogues of previously reported molecules. For example, highly proton-deficient metabolites from marine-derived *Streptomyces* species, marinopyrroles [16] and ammosamides [17], which both lack descriptive NMR signals, were only assigned using X-ray crystallographic techniques.

**Figure 1:**
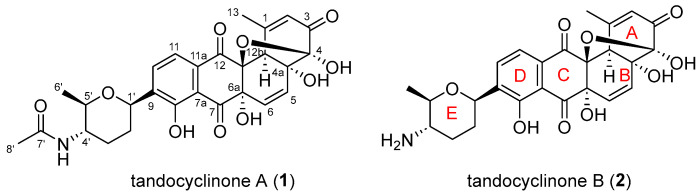
The structure of tandocyclinone A (**1**) and tandocyclinone B (**2**).

As part of our ongoing effort to discover natural products in the marine ecosystem, we collected an intertidal mudflat sample in Tando Port, Gyeonggi-do, Korea, and isolated a marine actinobacterial strain, *Streptomyces* sp. TDH03. A chemical profiling analysis of this strain based on LC/MS uncovered two proton-deficient natural products. The subsequent scale-up and chromatographic purification of the compounds enabled an analysis of their NMR spectra. Because these compounds, named tandocyclinones A and B (**1** and **2**, respectively), have fewer protons than the sum of ^13^C and heteroatoms, detecting the most exchangeable protons and obtaining 2D NMR correlations from these protons was crucial in the structure elucidation. By comparing the ^1^H NMR spectra of **1** in several NMR solvents, tetrahydrofuran (THF)-*d*_8_ was revealed as the best solvent, providing the maximum number of exchangeable protons in the ^1^H NMR spectrum (Figure S1). An analysis of the 1D and 2D NMR spectra acquired in THF-*d*_8_, along with UV, IR, and MS data, allowed for a confident planar structure elucidation of the proton-deficient tandocyclinones A and B (**1** and **2**) as new tetracyclic benz[*a*]anthracenes bearing *C*-glycosylation with a rare deoxyaminosugar (Figure 1).

The configurations of **1** and **2** were further assigned by analyzing *J*-coupling and ROESY correlations, applying modified Mosher’s method and Snatzke’s-induced CD experiments, and calculating ECD spectra. Here, we report the structure elucidation of the tandocyclinones and the biological evaluations in antibacterial, antifungal, antitubercular, and antiproliferative assays.

## 2. Results and Discussion

Tandocyclinone A (**1**) was purified as a brown amorphous powder with a molecular formula of C_27_H_27_NO_10_, determined using high-resolution electrospray ionization mass spectrometry (HR-ESI-MS). As the ratio of the number of hydrogens to the number of carbons and the other heteroatoms is less than 1, this seems to provide a warning that structure determination can be a challenging task, as proposed by Crews’ rule. Therefore, we carried out an NMR solvent investigation to choose an appropriate solvent to obtain more NMR information, such as exchangeable proton signals (Figure S1). As a result, a comparison of protic NMR solvent (CD_3_OD) with other aprotic deuterium solvents (DMSO-*d*_6_, CD_3_CN, acetone-*d*_6_, pyridine-*d*_5_, and THF-*d*_8_) revealed that only the ^1^H NMR spectrum in THF-*d*_8_ provided the maximum number of exchangeable protons with sharp signals (Figure S2). Hence, THF-*d*_8_ was chosen to acquire further NMR spectra for structure elucidation. In this solvent, the molecular formula was substantiated by its ^13^C NMR, which displayed a total of 27 signals (Table 1).

**Figure 2:**
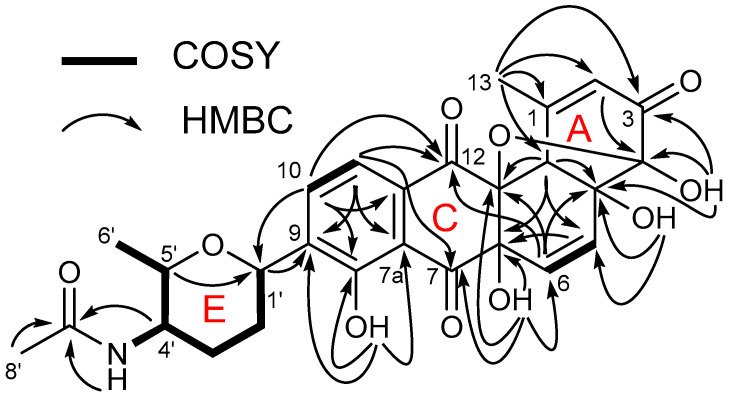
Key COSY and HMBC constructing the planar structure of tandocyclinone A (**1**).

A comprehensive analysis of the ^1^H, ^13^C, and HSQC spectral data (THF-*d*_8_) for **1** assigned one-bond ^1^H–^13^C correlations, four carbonyls, five fully substituted *sp*^2^ carbons, and four oxygenated quaternary carbons. In addition to these resonances, five exchangeable protons (*δ*_H_ 12.5, 6.83, 6.45, 6.07, and 5.24) due to the NH and OH groups were also observed. Given this information, the ^1^H–^1^H COSY data disclosed the presence of three spin systems, as shown in Figure 2. The first system, C-1′–C-6′, was recognized by a series of consecutive proton–proton COSY correlations of methine H-1′, methylene H_2_-2′, methylene H_2_-3′, methine H-4′, methine H-5′, and methyl H_3_-6′, respectively. The ^1^H–^13^C HMBC couplings from H-1′ to C-5′ and vice versa from H-5′ to C-1′ and the oxygen-bearing chemical shifts of C-1′ and C-5′ constructed the connectivity of C-1′ and C-5′ through an oxygen atom, elucidating a tetrahydropyran moiety (E ring). An exchangeable proton (*δ*_H_ 6.83) was subsequently coupled with the methine H-4′ of this moiety, and they further correlated with a carbonyl group C-7′ (*δ*_C_ 168.9) in the HMBC spectrum, indicating amide functionality and the exchangeable proton as an amide 4′-NH. The amide bond assignment was further evidenced by the observation of the characteristic ^15^N chemical shift in the amide region (*δ*_N_/*δ*_H_ 121.8/6.83) in the ^1^H–^15^N HSQC spectrum (Figure S6). The singlet methyl group CH_3_-8′ then showed a strong ^3^*J*_CH_ HMBC cross-peak to this amide carbon C-7′, consequently establishing the first moiety as an (*N*-acetyl)amino–methyl–tetrahydropyran-type moiety (E ring).

The second proton-spin system was defined by the COSY correlation, comprising H-10 (*δ*_H_ 7.84) and H-11 (*δ*_H_ 7.38). Their characteristic vicinal coupling constant (*J* = 8 Hz) was indicative of a three-bond coupling in a six-membered aromatic ring (D ring). As expected, the clear ^3^*J*_CH_ HMBC correlations from H-10 to C-8 (*δ*_C_ 159.1) and C-11a (*δ*_C_ 134.8), and from H-11 to C-9 (*δ*_C_ 139.1) and C-7a (*δ*_C_ 115.8) constructed a 1,2,3,4-tetrasubstituted six-membered aromatic system (D ring) composed of C-7a, C-8, C-9, C-10, C-11, and C-11a. An attachment of a hydroxy group to C-8 (*δ*_C_ 159.1) was then rationalized on the basis of the HMBC couplings of 8-OH (*δ*_H_ 12.5) to C-7a, C-8, and C-9. Furthermore, the E ring was connected to C-9 of this aromatic ring based on the key H-1′/C-8, C-9, and C-10 and H-10/C-1′ HMBC correlations. Subsequently, the strong H-11/C-12 and weak H-11/C-7 HMBC correlations led to the identification of two remaining substituents at C-11a and C-7a on this ring (D ring) as two ketone carbonyls C-12 (*δ*_C_ 190.5) and C-7 (*δ*_C_ 201.1), respectively. The ^4^*J*_C12H10_ HMBC coupling further supported the placement of ketone C-12. The ketone assignment at C-7 could explain a significantly deshielded proton chemical shift for 8-OH (*δ*_H_ 12.5), which could originate from their intramolecular hydrogen bonding.

The COSY cross-peak between two olefinic protons, H-5 (*δ*_H_ 6.20) and H-6 (*δ*_H_ 6.32), constituted the last proton–proton spin system. This was extended to a quaternary carbon C-6a (*δ*_C_ 79.6) through the H-5/C-6a and H-6/C-6a HMBC correlations. Then, an exchangeable proton showed HMBC relationships with C-6 (*δ*_C_ 126.0), C-6a, C-7, and C-12a (*δ*_C_ 89.2), indicating that this exchangeable proton belongs to a hydroxyl group at C-6a. These HMBC correlations, together with the HMBC couplings from H-6 to C-7 and C-12a, allowed us to connect C-6a to C-7 and C-12a. The additional long-range ^4^*J*_CH_ and even weak ^5^*J*_CH_ HMBC resonances of H-6 to C-12 and H-10 to C-12a, respectively, linked C-12 to C-12a, which then provided the formation of another ring of the molecule (C ring) as a cyclohexenedione moiety comprising C-6a–C-7–C-7a–C-11a–C-12–C-12a.

The connection of C-5 to C-4a was likewise deduced by the H-6/C-4a HMBC coupling. Subsequently, a singlet methine, H-12b, correlated to C-4a, C-5, C-6a, and C-12a via ^1^H–^13^C HMBC NMR correlations, demonstrating that C-4a, C-5, C-6, C-6a, C-12a, and C-12b were attributed to another ring of the structure (B ring). A hydroxyl group (4a-OH, *δ*_H_ 5.24) was positioned on C-4a due to its HMBC correlations to C-4a, C-5, and C-12b. The 2,3-bond heteronuclear correlations from the singlet methyl group (H_3_-13) to the fully substituted olefinic C-1, the olefinic methine C-2, and the methine C-12b signals, along with an even weaker 4-bond heteronuclear coupling from H_3_-13 to ketone C-3, were used to construct the α,*β*-unsaturated ketone moiety. This partial structure was connected to the B ring via a C-1–C-12b linkage. This moiety is further linked to a low-field quaternary carbon C-4 (*δ*_C_ 105.4) based on ^3^*J*_C4H2_ resonance. Then, the final exchangeable proton was assigned as 4-OH (*δ*_H_ 6.07) via 4-OH/C-4 HMBC coupling. 4-OH also displayed HMBC correlations with its neighboring carbons, C-3 and C-4a, firmly suggesting a direct C-4–C-4a connection, which was used to install the A ring. Five rings, including one six-membered aromatic ring and four other non-aromatic rings, two double bonds, and four carbonyl groups, constituted a total of fourteen double-bond equivalents (DBEs), while the molecular formula of **1** implied the presence of fifteen DBEs, thereby requiring one more DBE in the structure. Indeed, the downfield carbon chemical shift in C-4 (*δ*_C_ 105.4) was indicative of a dioxygenated carbon. Given that 4-OH was assigned at C-4 and C-12a must bear an oxygen atom by the ^13^C chemical shift in C-12a (*δ*_C_ 89.2), it was rationalized to have an ether bridge between C-4 and C-12a, which also satisfies the last unsaturation number. Hence, the planar structure of tandocyclinone A (**1**) was determined as illustrated in Figure 2.

**Figure 3:**
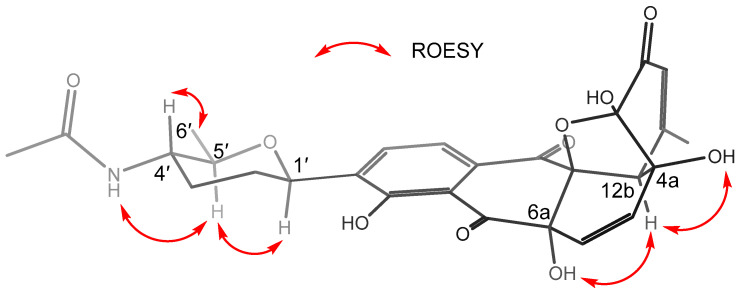
Key ROESY correlations for determination of the relative configuration of tandocyclinone A (**1**).

The relative configurations of the tetrahydropyran moiety (E ring) were assigned through ROESY correlations and proton–proton coupling constants (Figure 3). One large-value coupling constant (*J* = 11.0 Hz) and another small-value one (*J* = 2.0 Hz) of H-1′ implied the axial orientation of H-1′. H-4′ was coupled with H-5′ by a large-value coupling constant (*J* = 10.0 Hz) to place them on axial positions. This was also supported by the 4′-NH/H-5′ and H_3_-6′/ H-4′ ROESY correlations. The ROESY cross-peaks between H-1′ and H-5′ then indicated that they were cofacial, thus allowing for the assignment of the relative configurations for this moiety as 1′R*,4′S*,5′R*. In regard to the relative configurations of the stereocenters on rings A and B, the ROESY correlations were analyzed. The diagnostic ROESY resonances from H-12b to 4a-OH and 6a-OH suggested that they were all on the same phase of the molecule, while the C-4/C-12a ether bridge was on the other side of the molecule due to its rigid structure (Figure 3). Thereby, the relative configurations of the chiral centers on rings A and B were established as 4S*,4aR*,6aS*,12aS*,12bS*. The absolute configurations of tandocyclinone A (**1**) were deduced, together with tandocyclinone B (**2**), vide infra.

Tandocyclinone B (**2**) was isolated as a brown amorphous powder with the molecular formula of C_25_H_25_NO_9_ by using HR-ESI-MS data. The UV spectrum of **2** was identical to that of **1**, indicating the structural similarities between these two molecules. A careful comparison of the ^1^H and ^13^C NMR spectra of **2** with those of **1** revealed that the acetyl signal on the amide functional group was not present. The difference between **1** and **2** was due to the replacement of the amide group with the corresponding primary amine group. This was also confirmed by using combined 2D NMR analyses and the fact that the molecular weight was 42 Daltons less than that of **1**. The structure of **2** was further supported when derivatizing **2** with acetylation reagents under time control yielded an acetylation product that was identical to the HPLC retention time, mass data, and ^1^H NMR spectra of **1** (Figures S18 and S27). Moreover, the CD spectra for **2** were clearly identical to those in **1** (Figure S28); thus, tandocyclinone B (**2**) was assigned as a deacetylated derivative of **1**, and they shared the same absolute configurations for the stereogenic centers.

**Figure 4:**
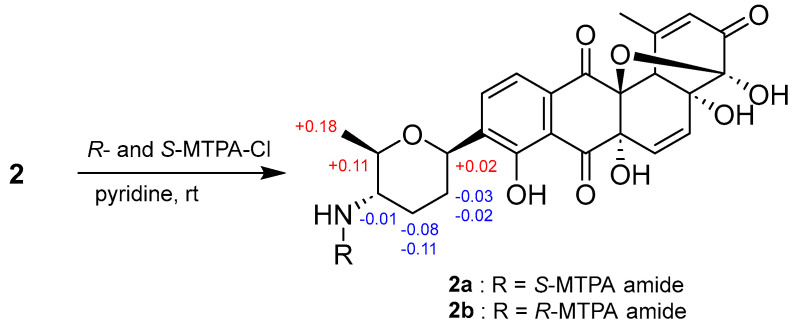
Δ*δ_S-R_* values for (*S*)- and (*R*)-MPTA amides (**2a** and **2b**) of **2**.

The absolute configurations of **1** and **2** were assigned by applying a modified Mosher’s method and Snatzke’s-induced CD experiments and calculating ECD spectra. First, the absolute configuration of the C-4′ chiral center (E ring) was determined by applying the modified Mosher’s method to the α-chiral primary amine [18]. Tandocyclinone B (**2**) with the primary amine group was derivatized with either (*R*)- or (*S*)-α-methoxy-α-(trifluoromethyl)phenylacetyl chloride (MTPA-Cl). The distribution of the Δ*δ*_H_ values (Δ*δ*_H_ = *δ_S_ − δ_R_*) around C-4′ of MTPA amides allowed the *S*-configuration for C-4′ to be assigned (Figure 4). Having established the relative configuration for the tetrahydropyran moiety (E ring) as discussed earlier, the absolute configurations of the chiral centers C-1′ and C-5′ were determined as 1′*R* and 5′*R*, respectively, for both tandocyclinones A and B (**1** and **2**).

**Figure 5:**
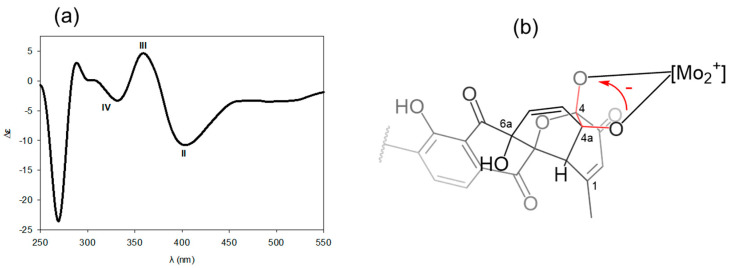
Determination of absolute configuration of *vic*-4,4a-diol moiety of **1** via Snatzke’s method. (**a**) ICD spectra of **1** in solution of Mo_2_(Oac)_4_ in DMSO. (**b**) The sign of the O–C–C-O dihedral angle in the cottonogenic derivative.

Then, the configuration of the 1,2-diol group at C-4 and C-4a of **1** was determined through the application of Snatzke’s method. This was performed by analyzing the sign of the O–C–C–O torsional angle gained from the induced CD (ICD) spectrum consisting of the in situ generations of chiral complexes between a chiral 1,2-diol compound and molybdenum (II) diacetate Mo_2_(OAc)_4_ (Figure 5) [19,20,21]. On the basis of the empirical rule, the negative sign of the O–C–C–O dihedral angle with diagnostic ICD bands such as negative cotton effects arising at 329 nm (band IV) and 406 nm (band II), and a positive one occurring at 357 nm (band II), established the absolute configurations of 4*S* and 4a*R* for **1** and **2**. Based on the relative configuration, the remaining absolute configurations of **1** and **2** were deduced as 6a*S*,12a*S*,12b*S*.

**Figure 6:**
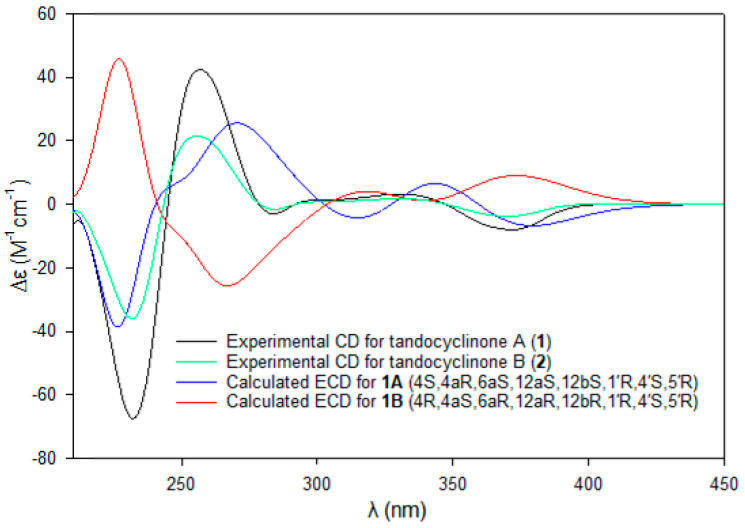
Experimental CD spectra for tandocyclinones A (**1**, black) and B (**2**, green) and calculated ECD spectra for **1A** (blue; 4*S*,4a*R*,6a*S*,12a*S*,12b*S*,1′*R*,4′*S*,5′*R*) and **1B** (red; 4*R*,4a*S*,6a*R*,12a*R*,12b*R*,1′*R*,4′*S*,5′*R*).

ECD calculations for two diastereomers (**1A** and **1B**) of tandocyclinone A (**1**), of which the absolute configurations for the E ring were fixed as 1′*R*, 4′*S*, and 5′*R*, as determined using the modified Mosher’s method, were also performed. A comparison of the experimental and calculated ECD spectra supported the absolute configurations determined using Snatzke’s method for both **1** and **2** (Figure 6).

The biological activities of tandocyclinones A and B (**1** and **2**) were evaluated in several ways. First, antibacterial and antifungal assays against pathogenic strains were carried out. It was found that **1** and **2** did not show any significant inhibitory effects (MIC > 128 μg/mL) against the tested bacterial strains (*Staphylococcus aureus* ATCC25923, *Enterococcus faecalis* ATCC19433, *Enterococcus faecium* ATCC19434, *Klebsiella pneumoniae* ATCC10031, *Salmonella enterica* ATCC14028, and *Escherichia coli* ATCC25922). Both **1** and **2** displayed weak antifungal activity against *Trichophyton mentagrophytes* IFM40996, with a minimum inhibitory concentration (MIC) value of 128 μg/mL (244 and 265 μM for **1** and **2**, respectively) but were inactive (MIC > 128 μg/mL) against the other pathogenic fungi strains, *Aspergillus fumigatus* HIC6094, *Trichophyton rubrum* NBRC9185, and *Candida albicans* ATCC10231. The antitubercular activity of tandocyclinones A and B (**1** and **2**) was evaluated against *Mycobacterium avium*, in which only **1** was active with an MIC_50_ value of 40.8 μM, whereas **2** showed no antimycobacterial activity. Finally, in the cell proliferation assay against the human carcinoma cell lines SNU638 (human gastric cancer cells), SK-HEP-1 (human liver cancer cells), A549 (human lung cancer cells), HCT116 (human colorectal cancer cells), and MDA-MB-231 (human breast cancer cells), **1** also showed weak cytotoxic effects against SNU638 and HCT116 cancer cells with IC_50_ values of 31.9 µM and 49.4 µM, respectively, while **2** again displayed no significant cytotoxicity (IC_50_ > 50 µM). These bioactivity evaluation data, in combination with the structural difference between **1** and **2**, suggested the important role of the *N*-acetylation group for bioactivity effects.

## 3. Materials and Methods

### 3.1. General Experimental Procedures

Optical A JASCO P-2000 polarimeter (sodium light source, JASCO, Easton, PA, USA) with 1 cm cell was used to measure optical rotation data. Applied Photophysics Chirascan-plus CD spectrometer (Applied Photophysics, Leatherhead, Surrey, UK) with 1 mm and 2 mm CD cells was utilized to collect ultraviolet (UV) and circular dichroism (CD) spectroscopic data. A JASCO FT/IR-4200 spectrometer (FT/IR-4200, Tokyo, Japan) was used to acquire infrared (IR) spectra. NMR spectra were collected at Seoul National University’s College of Pharmacy, Seoul, Republic of Korea, using Bruker Avance 800 MHz (Bruker, Billerica, MA, USA) and JEOL 400 MHz (JEOL, Tokyo, Japan) spectrometers. An Agilent Technologies 6130 Quadrupole mass spectrometer coupled with an Agilent Technologies 1200 series high-performance liquid chromatography (HPLC) apparatus (Agilent Technologies, Santa Clara, CA, USA) enabled low-resolution electrospray ionization mass spectroscopic (LR-ESI-MS) data to be obtained. High-resolution electrospray ionization mass spectroscopic (HR-ESI-MS) data were collected in the National Instrumentation Center for Environmental Management (NICEM) at the College of Agriculture and Life Sciences at Seoul National University using an AB SCIEX Q-TOF 5600 HR-MS spectrometer (Framingham, MA, USA).

### 3.2. Isolation of the Bacterial Strain

A mud sample was collected from the intertidal mudflat in Tando Port (37.192541° N, 126.644974° E), Seongam-dong, Danwon-gu, Ansan-si, Gyeonggi-do, the Republic of Korea, on 19 April 2022. Then, the sample was diluted in sterilized deionized water and spread onto various agar media for strain isolation, including actinomycete isolation agar medium, SC agar, R2A agar, Czapek-Dox agar medium, ISP1 agar medium, and ISP4 agar medium (all media were supplemented with 100 mg/L of cycloheximide and 28 g/L of sea salts). The plates were incubated at 30 °C for two weeks. A colony with actinomycete-like morphology was isolated on an ISP4 agar medium and further streaked onto a fresh YEME saline agar plate (4 g of yeast extract, 10 g of malt extract, 4 g of d(+)-glucose, 28 g of sea salt, and 18 g of agar powder per 1 L of sterilized water) to obtain a pure bacterial strain (strain name TDH03). Comparing the 16S rDNA sequence revealed that it belongs to the *Streptomyces* genus (GenBank accession number OR195704) and is most closely related to *Streptomyces hyderabadensis* (98.7 similarity, GenBank accession number FM998652). The TDH03 strain was stored in 30% glycerol aqueous solution at −80 °C for further experiments.

### 3.3. Cultivation and Extraction

The TDH03 strain was resurrected on a YEME saline agar plate for 14 days at 30 °C. A loop of the mycelium was inoculated in 50 mL of DSY saline medium (2 g of yeast extract, 4 g of soytone, 10 g of dextrin, and 28 g of sea salt in 1 L of sterilized water) in a 125 mL Erlenmeyer flask and incubated on a rotary shaker at 200 rpm and 30 °C. Following 3 days of incubation, 10 mL of the seed liquid culture was transferred to 500 mL Erlenmeyer flasks containing 200 mL of DSY saline medium. After another 2-day growth (170 rpm and 30 °C), 20 mL of the broth culture was transferred to 1 L of DSY saline medium in 2.5-L Ultra Yield^®^ flasks (Thompson). After 5 days of cultivation with shaking at 160 rpm and 30 °C, these 30 flasks were extracted repeatedly with 1.5 volumes of ethyl acetate (EtOAc). The EtOAc layer was passed through anhydrous sodium sulfate to remove residual water before being evaporated in vacuo to yield 6.5 g of the dried extract.

### 3.4. Purification of Tandocyclinone A and B (***1*** and ***2***, Respectively)

The above crude extract was adsorbed onto Celite and fractionated via open-column chromatography (60 × 40 mm, YMC*GEL ODS-A, 12 nm, S-75 μm) with a step gradient of 300 mL of MeOH in H_2_O solution (20%, 40%, 60%, 80%, and 100%). To monitor the presence of tandocyclinones A and B (**1** and **2**, respectively), a portion of each fraction was analyzed using LC/MS with a Phenomenex column (Luna^®^, 100-5-C_18_, 100 × 4.6 mm, 5 μm) under gradient systems (flow rate: 0.7 mL/min; UV detection: 210, 230, 254, 280, and 360 nm; 10–100% CH_3_CN–H_2_O with 0.1% formic acid over 20 min). Both **1** and **2** were detected in the 60% MeOH-H_2_O fraction. This fraction was evaporated and redissolved in MeOH to be chromatographed via semi-preparative reversed-phase HPLC (Kromasil^®^, 100-5-C_18_, 250 × 10 mm, 5 μm) under gradient solvent conditions (flow rate: 2 mL/min; UV detection: 240 nm; 20–50% CH_3_CN–H_2_O over 30 min). Tandocyclinones A (**1**) and B (**2**) were eluted at 32 min and 17 min, respectively. Tandocyclinone A (**1**) was further subjected to the same HPLC system under isocratic conditions (40% CH_3_CN–H_2_O; flow rate: 2 mL/min; UV detection: 240 nm) to yield pure **1** (24 mg). In the same manner, tandocyclinone B (**2**) was purified again under isocratic conditions (30% CH_3_CN–H_2_O; flow rate: 2 mL/min; UV detection: 240 nm) to yield pure **2** (17 mg).

*Tandocyclinone A* (**1**): brown amorphous powder, $[\alpha {]}_{D}^{20}$ − 12 (c = 0.1, MeOH); UV (MeOH) λ_max_ (log ε) 239 nm (4.34), 353 nm (3.66); IR (neat) ν_max_ 3334, 2978, 2357, 2318, 1709, 1659, 1548, 1429, 1379, 1243, 1200 cm^−1^; NMR data in THF-*d*_8_, Table 1; HR-ESI-MS [M + H]^+^ *m*/*z* 526.1705 (calcd for C_27_H_28_NO_10_, 526.1708).

*Tandocyclinone B* (**2**): brown amorphous powder, $[\alpha {]}_{D}^{20}$ − 42 (c = 0.1, MeOH); UV (MeOH) λ_max_ (log ε) 240 nm (4.37), 353 nm (3.69); IR (neat) ν_max_ 3164, 2949, 2355, 2317, 1713, 1663, 1548, 1428, 1378, 1241, 1203 cm^−1^; NMR data in THF-*d*_8_, Table 1; HR-ESI-MS [M + H]^+^ *m*/*z* 484.1601 (calcd for C_25_H_26_NO_9_, 484.1602).

### 3.5. Acetylation of Tandocyclinone B (***2***) to Yield Tandocyclinone A (***1***)

Tandocyclinone B (**2**, 2 mg) was initially dried in a vacuum for 15 h and then dissolved into 1 mL of anhydrous pyridine under argon (Ar) gas purging. Next, 20 µL of acetic anhydride was added to the solution, and the reaction was maintained under agitation by stirring at room temperature. After 15 min of reaction, 50 µL of water was introduced to quench the reaction. The solvent was removed under vacuum for 2 h. The acetylation product was purified via semi-preparative reversed-phase HPLC (Kromasil^®^, 100-5-C_18_, 250 × 10 mm, 5 μm) under gradient solvent conditions (flow rate: 2 mL/min; UV detection: 240 nm; 20–50% CH_3_CN–H_2_O over 30 min). The pure acetylation product of **2** was analyzed using LC/MS to compare LC/MS profiles (retention time, UV and MS spectra) with tandocyclinone A (**1**). ^1^H NMR was also acquired to confirm that the acetylation product of **2** was indeed **1** (Figure S18).

### 3.6. Preparing MTPA Amides of Tandocyclinone B (***2***)

Purified **2** (4 mg) was equally divided into two 40 mL vials. After 15 h of lyophilization, each vial was dissolved in 1 mL of anhydrous pyridine under argon gas and further treated with 30 µL of *(R*)- and (*S*)-α-methoxy-α-(trifluoromethyl) phenylacetyl chloride ((*R*)-MTPA-Cl and (*S*)- MTPA-Cl). The reactions were performed with stirring for 2 h at room temperature. To each vial, 200 µL of MeOH was added to quench the reaction. The mixtures were subsequently dried in a vacuum for 3 h, and the amide products were purified using the semi-preparative reversed-phase HPLC (Kromasil 100-5-C_18_, 250 × 10 mm, 5 µm) under gradient solvent conditions (50–90% CH_3_CN–H_2_O over 50 min; flow rate: 2 mL/min; UV detection: 240 nm). The (*S*)-MTPA amide (**2a**) and (*R*)-MTPA amide (**2b**) of **2** were eluted at 47.0 min (1.2 mg) and 48.0 min (1.4 mg), respectively. The molecular formulas of both **2a** and **2b** were assigned to be C_35_H_32_F_3_NO_11_ via HR-ESI-MS analysis (Figures S33 and S34). The ^1^H, COSY, HSQC, and HMBC NMR data were recorded to confirm the structure of MTPA amide products and calculate Δ*δ*_H_ values (Δ*δ*_H_ = *δ_S_* − *δ_R_*) around C-4′.

(*S*)-MTPA amide (**2a**) (1.2 mg): ^1^H NMR (CD_3_OD, 800 MHz) *δ*_H_ 7.851 (1H, d, *J* = 8.0 Hz), 7.553–7.526 (2H, m), 7.437–7.401 (4H, m), 6.395 (1H, dd, *J* = 10.0, 1.0 Hz), 6.243 (1H, dd, *J* = 10.0, 1.0 Hz), 6.079 (1H, s), 4.807 (1H, dd, *J* = 11.0, 2.5 Hz), 3.715 (1H, ddd, *J* = 11.0, 10.5, 4.0 Hz), 3.678–3.660 (1H, m), 3.446 (3H, s), 3.276 (1H, s), 2.449 (3H, s), 2.180–2.152 (1H, m), 1.936 (1H, dq, *J* = 12.5, 4.0 Hz), 1.744 (1H, qd, *J* = 12.5, 4.0 Hz), 1.435 (1H, qd, *J* = 13.5, 4.0 Hz), 1.268 (3H, d, *J* = 6.0 Hz).

(*R*)-MTPA amide (**2b**) (1.4 mg): ^1^H NMR (CD_3_OD, 800 MHz) *δ*_H_ 7.836 (1H, d, *J* = 8.0 Hz), 7.598–7.568 (2H, m), 7.431–7.393 (4H, m), 6.391 (1H, d, *J* = 10.0 Hz), 6.241 (1H, d, *J* = 10.0 Hz), 6.079 (1H, dd, *J* = 1.5, 1.5 Hz), 4.802–4.759 (1H, m), 3.736–3.702 (1H, m), 3.553 (1H, dd, *J* = 10.0, 6.0 Hz), 3.505 (3H, s), 3.272 (1H, d, *J* = 1.0 Hz), 2.447 (3H, d, *J* = 1.0 Hz), 2.211–2.167 (1H, m), 2.022–1.993 (1H, m), 1.848–1.794 (1H, m), 1.479–1.425 (1H, m), 1.084 (3H, d, *J* = 6.0 Hz).

### 3.7. Determination of the Absolute Configuration of 1,2-4,4a-Diol Moiety of 1 by Snatzke’s Method

The CD data were collected with a 1 mm cell at room temperature using step scans of 0.5 nm per step over a range of 200–550 nm. Initially, **1** was dissolved into DMSO (0.5 mg/mL, 0.95 mM), and the CD spectrum was recorded. Then, dimolybdenum tetraacetate (Mo_2_(OAc)_4_) was dissolved in the above solution containing DMSO and **1** (the concentration of Mo_2_(OAc)_4_ was 0.6 mg/mL and 1.41 mM, and the molar ratio of **1** to the Mo_2_(OAc)_4_ was 1:1.5). The first induced CD (ICD) data were measured immediately after dissolving with Mo_2_(OAc)_4_, and the mixture was recorded every 5 min until 30 min after mixing, when a stationary spectrum was reached. Finally, the ICD spectrum of the ligand–metal complex was obtained by subtracting the inherent CD data of **1** from the stationary ICD data. The observed signs of the diagnostic bands were related to the absolute configuration at *vic*-4,4a-diol moiety in accordance to the empirical rule.

### 3.8. Conformational Search and ECD Calculations for ***1***

Two diastereomers of **1** were energetically minimized using the Chem3D Ultra (version 19.0, PerkinElmer, Waltham, USA) with the MM2 force field. A conformational search for the energy-minimized diastereomers of **1** was next applied via the MacroModel (version 11.9, Schrödinger LLC, New York, NY, USA) interfaced with Maestro (version 11.5, Schrödinger LLC) in Merck molecular force field (MMFF94) at gas phase, a mixed torsional/low-mode sampling method with a 20 kJ/mol upper energy limit and 10,000 as the maximum number of steps. The generated conformers were further geometrically optimized by using density functional theory (DFT) calculations using TmoleX 4.3.2 and Turbomole 7.2 (COSMOLogic GmbH, Leverkusen, Germany), the basis set 6-31G(d) for all atoms, and the B3LYP functional level in the gas phase. The optimized stable conformers with a Boltzmann population of over 1% were selected for ECD calculation. Then, ECD spectra for each conformer were simulated by the implementation of the time-dependent density functional theory (TD-DFT) method at the B3LYP/def2-TZVP level in the gas phase. Rotatory strengths for a total of 30 excited states were calculated. These conformers were all weighted by the Boltzmann distribution, and overall ECD spectra were visualized via SpecDis 1.71 (University of Würzburg, Würzburg, Germany) software [22] with a sigma/gamma value of 0.24 eV.

### 3.9. Antibacterial Assays

The procedures for the antibacterial activity assay of tandocyclinones A and B (**1** and **2**) were carried out according to the method of the Clinical and Laboratory Standard Institute (CLSI), as described previously [23]. Six Gram-positive (*Staphylococcus aureus* ATCC25923, *Enterococcus faecalis* ATCC19433, and *Enterococcus faecium* ATCC 19434) and Gram-negative (*Klebsiella pneumoniae* ATCC10031, *Salmonella enterica* ATCC14028, and *Escherichia coli* ATCC25922) bacterial strains were obtained from the American Type Culture Collection (ATCC). The bacteria were incubated overnight in Mueller Hinton Broth (MHB, BD Difco, Sparks, MD, USA) at 37 °C, harvested using a centrifuge, washed twice with sterilized deionized water, and then adjusted to match the turbidity of a 0.5 McFarland standard at a wavelength of 625 nm. Stock solutions of the tandocyclinones were dissolved in DMSO. Each stock solution was diluted with MHB to provide serial 2-fold dilutions in the range of 1–128 μg/mL (DMSO concentration was maintained at 1% according to CLSI guidelines). In each well of a 96-well plate, 90 μL of MHB containing the test compound was mixed with 10 μL of broth containing the test bacterium (final concentration: 5 × 10^5^ cfu/mL). The plates were then incubated for 12 h at 37 °C. MICs were defined as the lowest concentration of the test compound that inhibited bacterial growth. Ampicillin (Duchefa, Amsterdam, Netherlands) and tetracycline (Sigma-Aldrich, St. Louis, MO, USA) were used as positive controls.

### 3.10. Antifungal Assays

To measure the MICs of the tandocyclinones, four human pathogenic fungal strains (*Aspergillus fumigatus* HIC6094, *Trichophyton rubrum* NBRC 9185, *Trichophyton mentagrophytes* IFM40996, and *Candida albicans* ATCC10231) were selected and tested according to the method of the CLSI, as described previously [23]. *Aspergillus fumigatus* HIC6094 was obtained from the National Institute of Health Sciences (Kawasaki City, Kanagawa, Japan). *Trichophyton rubrum* NBRC 9185 was obtained from the NITE Biological Resource Center (Tokyo, Japan). *Trichophyton mentagrophytes* IFM40996 was obtained from the Institute for Fermentation (Osaka, Japan). *Candida albicans* ATCC10231 was obtained from the American Type Culture Collection (ATCC). Stock solutions of the compound were prepared in DMSO and diluted in Roswell Park Memorial Institute (RPMI) 1640 broth (Sigma-Aldrich) in the range of 1–128 μg/mL. In each well of a 96-well plate, 90 μL of RPMI containing the test compound was mixed with 10 μL of broth containing the test fungus (final concentration: 5 × 10^5^ cfu/mL). The plates were incubated for 24 h (for *C. albicans*), 48 h (for *A. fumigatus*), or 96 h (for *T. rubrum* and *T. mentagrophytes*) at 35 °C. MICs were determined as the lowest concentration of test compound that inhibited fungal growth. A culture with DMSO (1%) and a culture supplemented with amphotericin B (Sigma-Aldrich) were used as the negative and positive controls, respectively.

### 3.11. Resazurin Microtiter Assay (REMA) Plate Testing for Mycobacterium avium

The MIC values of tandocyclinones A and B were determined using Resazurin microtiter assay. *M. avium* (GenBank, CP000479.1) was kindly provided by Professor Hwa-Jung Kim (School of Medicine, Chungnam National University). The *M. avium* strain was grown at 37 ℃ with agitation at 180 rpm in a Middlebrook 7H9 culture medium (Difco) supplemented with 10% albumin-dextrose-catalase (ADC; Difco), 0.2% glycerol, and 0.05% Tween 80. To evaluate the MIC, the cation-adjusted Mueller–Hinton (CAMH) medium (Sigma) supplemented with 20 mg/L calcium chloride and 10 mg/L magnesium chloride was used. The exponential-phase bacterial cultures and a 2-fold serial dilution of the test compound solutions were added to each well of a 96-well microtiter plate, and the final bacterial optical density at 600 nm (OD_600_) was adjusted to 0.005. Clarithromycin was used as the positive control, and each plate also contained a DMSO-negative control. The plates were then incubated at 37 ℃ for 4 days, and then 0.025% [*w*/*v*] resazurin was added to each well. After overnight incubation, the fluorescence was measured at 560–590 nm using a SpectraMax M3 multimode microplate reader (Molecular Devices, San Jose, CA, USA). The concentrations required to inhibit bacterial growth by 50% (MIC_50_) were calculated by using GraphPad Prism 8 software (GraphPad Software, Inc., La Jolla, CA, USA).

### 3.12. Cell Proliferation Assay

The cytotoxicity of tandocyclinones A and B were evaluated as previously described using a sulforhodamine B (SRB) assay [24,25]. The five human cancer cell lines, A549 (human lung cancer cells), MDA-MB-231 (human breast cancer cells), HCT116 (human colon cancer cells), SNU-638 (human gastric carcinoma), and SK-Hep-1 (human hepatic carcinoma), were tested with etoposide as a positive control. The A549, MDA-MB-231, HCT116, and SK-Hep-1 cancer cell lines were obtained from the American Type Culture Collection (Manassas, VA, USA).

## 4. Conclusions

In this report, we discovered two new metabolites, tandocyclinones A and B (**1** and **2**), from marine-derived *Streptomyces* sp. The ratios of hydrogens to heteroatroms in the molecular formulae of **1** and **2** were less than 1, which provided a warning for structure elucidation according to Crews’ rule. The application of an appropriate NMR solvent to observe the maximal exchangeable proton signals enabled the observation of their heteronuclear correlations and the successful determination of their structures. The tandocyclinones (**1** and **2**) possess intriguing structural features, including a highly oxygenated tetracyclic benz[*a*]anthracene system, an ether bridge, and 8-*C*-glycosylation with a deoxyaminosugar. They are most structurally related to gephyromycin, with the first angucyclinone bearing an ether bridge [26]. However, **1** and **2** do not possess the typical angucyclinone moiety, which is present in gephyromycin. The tandocyclinones contain the methyl group at C-1 and ketone functionality at C-3, which are different from angucyclinones that usually have the methyl and ketone groups at C-3 and C-1, respectively [27]. This could be explained by the different arrangements of building blocks during the biosynthesis process (Figure S36 and Scheme S1). Moreover, the ether bridge in gephyromycin originates from two oxygen-bond carbons to constitute an additional six-membered ring, while one oxygenated carbon and another ketal carbon in **1** and **2** formed an ether-bridged five-membered ring. Furthermore, **1** and **2** feature a *C*-glycoside-linked deoxyaminosugar at the D ring. Interestingly, we identified a known pyranonaphthoquinone, BE-54238A, by performing a comparison of the spectroscopic data with the literature values (Figures S37 and S38), with the involvement of a 4′-amino-4′-deoxy-α-d-amicetose (α-d-forasmine) as the deoxyaminosugar biosynthetic precursor during 8-*C*-glycosylation [28,29]. Considering the E ring structure of **1** and **2**, it is tempting to suggest that the E ring could originate from the *C*-deoxyaminosugar glycosylation pathway of α-d-forasmine. The α-d-forasmine is often reported in either an *N*-methylated form, as reported in spinosyns [30] and qinimycins [29], or an unusually fused structure, as observed in BE-54238A and glenthenamines [28]. Interestingly, the amine functionality of the α-d-forasmine in **2** is still intact, while **1** bears *N*-acetylated α-d-forasmine. The intact and acetylated forms were observed for the first time in natural products, to the best of our knowledge. In terms of their biological activities, tandocyclinones A and B (**1** and **2**) displayed weak antifungal activity against *Trichophyton mentagrophytes* IFM40996. Furthermore, **1** was also found to have weak inhibitory activity against *Mycobacterium avium* and antiproliferative activity against SNU638 and HCT116 cancer cells, respectively, while tandocyclinone B (**2**) was inactive for these assays. The discovery and structure elucidation of the tandocyclinones highlight that marine-derived actinobacteria produce structurally interesting but challenging natural products with respect to structure elucidation.

## Figures and Tables

**Table 1:** ^1^H (800 MHz) and ^13^C (200 MHz) NMR spectral data of tandocyclinones A (**1**) and B (**2**) in THF-*d*_8_.

Position	1			2		
	*δ*_C_/*δ*_N_	Type	*δ*_H_, Mult (*J* in Hz)	*δ*_C_	Type	*δ*_H_, Mult (*J* in Hz)
1	164.4	C		164.4	C	
2	124.6	CH	6.01, d (1.0)	124.6	CH	6.00, dd (1.0, 1.0)
3	193.3	C		193.5	C	
4	105.4	C		105.5	C	
4a	82.8	C		82.9	C	
5	138.5	CH	6.20, d (10.0)	138.4	CH	6.20, d (10.0)
6	126.0	CH	6.32, d (10.0)	126.1	CH	6.32, d (10.0)
6a	79.6	C		79.6	C	
7	201.1	C		201.1	C	
7a	115.8	C		115.8	C	
8	159.1	C		159.2	C	
9	139.1	C		139.2	C	
10	133.9	CH	7.84, d (8.0)	133.8	CH	7.78, d (8.0)
11	119.0	CH	7.38, d (8.0)	118.9	CH	7.38, d (8.0)
11a	134.8	C		134.8	C	
12	190.5	C		190.5	C	
12a	89.2	C		89.2	C	
12b	54.1	CH	3.18, s	54.1	CH	3.19, d (1.0)
13	26.7	CH_3_	2.41, br s	26.7	CH_3_	2.41, d (1.0)
1′	74.2	CH	4.72, dd (11.0, 2.0)	74.2	CH	4.70, dd (11.0, 2.0)
2′a	33.0	CH_2_	2.14, dddd (13.5, 3.0, 3.0, 2.0)	33.1	CH_2_	2.11, dddd (13.0, 4.0, 3.0, 2.0)
2′b			1.39, m			1.27, m
3′a	31.9	CH_2_	1.99, m	33.5	CH_2_	2.00, ddd (13.0, 4.0, 4.0)
3′b			1.55, m			1.50, dddd (13.0, 13.0, 13.0, 4.0)
4′	51.3	CH	3.66, m	54.2	CH	2.45, m
5′	79.1	CH	3.39, dq (10.0, 6.0)	80.5	CH	3.29, dq (9.0, 6.0)
6′	19.2	CH_3_	1.22, d (6.0)	19.0	CH_3_	1.28, d (6.0)
7′	168.9	C				
8′	22.9	CH_3_	1.81, s			
4-OH			6.07, s			
4a-OH			5.24, s			
6a-OH			6.45, s			
8-OH			12.5, s			
4′-NH	121.8	NH	6.83, d (9.0)			

## Data Availability

All data are contained within this article and the Supplementary Materials.

## Supplementary Materials

The following supporting information can be downloaded at https://www.mdpi.com/article/10.3390/md21090500/s1, Figure S1. Comparison of ^1^H NMR spectra of tandocyclinone A (**1**) in various solvents (pyridine-*d*_5_, acetone-*d*_6_, DMSO-*d*_6_, CD_3_CN, THF-*d*_8_, and CD_3_OD); Figure S2. ^1^H NMR spectrum of tandocyclinone A (**1**) at 800 MHz in THF-*d*_8_; Figure S3. ^13^C NMR spectrum of tandocyclinone A (**1**) at 200 MHz in THF-*d*_8_; Figure S4. COSY NMR spectrum of tandocyclinone A (**1**) at 800 MHz in THF-*d*_8_; Figure S5. HSQC NMR spectrum of tandocyclinone A (**1**) at 800 MHz in THF-*d*_8_; Figure S6. ^1^H–^15^N HSQC NMR spectrum of tandocyclinone A (**1**) at 800 MHz in THF-*d*_8_; Figure S7. HMBC NMR spectrum of tandocyclinone A (**1**) at 800 MHz in THF-*d*_8_; Figure S8. ROESY NMR spectrum of tandocyclinone A (**1**) at 800 MHz in THF-*d*_8_; Figure S9. ^1^H NMR spectrum of tandocyclinone B (**2**) at 800 MHz in THF-*d*_8_; Figure S10. ^13^C NMR spectrum of tandocyclinone B (**2**) at 200 MHz in THF-*d*_8_; Figure S11. COSY NMR spectrum of tandocyclinone B (**2**) at 800 MHz in THF-*d*_8_; Figure S12. HSQC NMR spectrum of tandocyclinone B (**2**) at 800 MHz in THF-*d*_8_; Figure S13. HMBC NMR spectrum of tandocyclinone B (**2**) at 800 MHz in THF-*d*_8_; Figure S14. ROESY NMR spectrum of tandocyclinone B (**2**) at 800 MHz in THF-*d*_8_; Figure S15. ^1^H NMR spectrum of tandocyclinone B (**2**) at 400 MHz in CD_3_OD; Figure S16. ^1^H NMR spectrum of acetylation product of tandocyclinone B (**2**) at 400 MHz in CD_3_OD; Figure S17. 1H NMR spectrum of tandocyclinone A (1) at 400 MHz in CD3OD; Figure S18. Comparison of ^1^H NMR spectra of **2**, acetylation product of **2** and **1** in CD_3_OD; Figure S19. ^1^H NMR spectrum of (*S*)-MTPA amide (**2a**) of tandocyclinone B (**2**) at 800 MHz in CD_3_OD; Figure S20. COSY NMR spectrum of (*S*)-MTPA amide (**2a**) of tandocyclinone B (**2**) at 800 MHz in CD_3_OD; Figure S21. HSQC NMR spectrum of (*S*)-MTPA amide (**2a**) of tandocyclinone B (**2**) at 800 MHz in CD_3_OD; Figure S22. HMBC NMR spectrum of (*S*)-MTPA amide (**2a**) of tandocyclinone B (**2**) at 800 MHz in CD_3_OD; Figure S23. ^1^H NMR spectrum of (*R*)-MTPA amide (**2b**) of tandocyclinone B (**2**) at 800 MHz in CD_3_OD; Figure S24. COSY NMR spectrum of (*R*)-MTPA amide (**2b**) of tandocyclinone B (**2**) at 800 MHz in CD_3_OD; Figure S25. HSQC NMR spectrum of (*R*)-MTPA amide (**2b**) of tandocyclinone B (**2**) at 800 MHz in CD_3_OD; Figure S26. HMBC NMR spectrum of (*R*)-MTPA amide (**2b**) of tandocyclinone B (**2**) at 800 MHz in CD_3_OD; Figure S27. Liquid chromatography profiles of tandocyclinone B (**2**), acetylation product of **2**, and tandocyclinone A (**1**); Figure S28. CD spectra for tandocyclinones A (**1**, red), and B (**2**, blue); Figure S29. IR spectrum of tandocyclinone A (**1**); Figure S30. IR spectrum of tandocyclinone B (**2**); Figure S31. HR-ESI-MS data of tandocyclinone A (**1**); Figure S32. HR-ESI-MS data of tandocyclinone B (**2**); Figure S33. HR-ESI-MS data of (*S*)-MTPA amide (**2a**) of tandocyclinone B (**2**); Figure S34. HR-ESI-MS data of (*R*)-MTPA amide (**2b**) of tandocyclinone B (**2**); Figure S35. The simulated models of two possible diastereomers, **1A** (4*S*,4a*R*,6a*S*,12a*S*,12b*S*,1′*R*,4′*S*,5′*R*) and **1B** (4*R*,4a*S*,6a*R*,12a*R*,12b*R*,1′*R*,4′*S*,5′*R*), of tandocyclinone A (**1**); Figure S36. The proposed polyketide cyclization patterns of (a) tandocyclinones and (b) angucyclinones; Scheme S1. Proposed biosynthetic pathways of tandocyclinones A and B (**1** and **2**); Figure S37. ^1^H NMR (600 MHz, DMSO-*d*_6_) and UV-vis (inset) spectra of BE-54238A (mass 397) from reference; Figure S38. ^1^H NMR (800 MHz, DMSO-*d*_6_), UV-vis (inset) and MS (positive mode, inset) spectra of TDH03.397; Table S1. Energy analysis for diastereomers **1A** (4*S*,4a*R*,6a*S*,12a*S*,12b*S*,1′*R*,4′*S*,5′*R*) and **1B** (4*R*,4a*S*,6a*R*,12a*R*,12b*R*,1′*R*,4′*S*,5′*R*) of tandocyclinone A (**1**); Table S2. ECD calculation of conformer 1 of diastereomer **1A**; Table S3. ECD calculation of conformer 2 of diastereomer **1A**; Table S4. ECD calculation of conformer 3 of diastereomer **1A**; Table S5. ECD calculation of conformer 1 of diastereomer **1B**; Table S6. ECD calculation of conformer 2 of diastereomer **2B**; and Table S7. ECD calculation of conformer 3 of diastereomer **2B**.

## References

[1] Breton R.C., Reynolds W.F. (2013). Using NMR to identify and characterize natural products. Nat. Prod. Rep..

[2] White K.N., Amagata T., Oliver A.G., Tenney K., Wenzel P.J., Crews P. (2008). Structure Revision of Spiroleucettadine, a sponge alkaloid with a bicyclic core meager in H-atoms. J. Org. Chem..

[3] Sidebottom P.J. (2021). Crews’ rule—Still useful but often misquoted. Magn. Reson. Chem..

[4] Schoefberger W., Schlagnitweit J., Müller N., Webb G.A. (2011). Chapter 1—Recent developments in heteronuclear multiple-bond correlation experiments. Annual Reports on NMR Spectroscopy.

[5] Furrer J., Webb G.A. (2011). Chapter 6—Recent developments in HMBC studies. Annual Reports on NMR Spectroscopy.

[6] Williamson R.T., Buevich A.V., Martin G.E., Parella T. (2014). LR-HSQMBC: A Sensitive NMR Technique to probe very long-range heteronuclear coupling pathways. J. Org. Chem..

[7] Gyöngyösi T., Nagy T.M., Kövér K.E., Sørensen O.W. (2018). Distinguishing between two- and three-bond correlations for all ^13^C multiplicities in heteronuclear NMR spectroscopy. Chem. Commun..

[8] Wang Y., Fan A., Cohen R.D., Dal Poggetto G., Huang Z., Yang H., Martin G.E., Sherer E.C., Reibarkh M., Wang X. (2023). Unequivocal identification of two-bond heteronuclear correlations in natural products at nanomole scale by i-HMBC. Nat. Commun..

[9] Martin G.E., Webb G.A. (2011). Chapter 5—Using 1,1- and 1,n-ADEQUATE 2D NMR data in structure elucidation protocols. Annual Reports on NMR Spectroscopy.

[10] Saurí J., Ndukwe I.E., Reibarkh M., Liu Y., Williamson R.T., Martin G.E., Webb G.A. (2019). Chapter One—New variants of the ADEQUATE experiments. Annual Reports on NMR Spectroscopy.

[11] Senior M.M., Williamson R.T., Martin G.E. (2013). Using HMBC and ADEQUATE NMR data to define and differentiate long-range coupling pathways: Is the Crews rule obsolete?. J. Nat. Prod..

[12] Blunt J.W., Carroll A.R., Copp B.R., Davis R.A., Keyzers R.A., Prinsep M.R. (2018). Marine natural products. Nat. Prod. Rep..

[13] Jiménez C. (2018). Marine natural products in medicinal chemistry. ACS Med. Chem. Lett..

[14] Molinski T.F., Dalisay D.S., Lievens S.L., Saludes J.P. (2009). Drug development from marine natural products. Nat. Rev. Drug Discov..

[15] Behie S.W., Bonet B., Zacharia V.M., McClung D.J., Traxler M.F. (2016). Molecules to ecosystems: Actinomycete natural products in situ. Front. Microbiol..

[16] Hughes C.C., Prieto-Davo A., Jensen P.R., Fenical W. (2008). The marinopyrroles, antibiotics of an unprecedented structure class from a marine *Streptomyces* sp.. Org. Lett..

[17] Hughes C.C., MacMillan J.B., Gaudêncio S.P., Jensen P.R., Fenical W. (2009). The ammosamides: Structures of cell cycle modulators from a marine-derived *Streptomyces* species. Angew. Chem. Int. Ed..

[18] Seco J.M., Quiñoá E., Riguera R. (2001). A practical guide for the assignment of the absolute configuration of alcohols, amines and carboxylic acids by NMR. Tetrahedron Asymmetry.

[19] Górecki M., Jabłońska E., Kruszewska A., Suszczyńska A., Urbańczyk-Lipkowska Z., Gerards M., Morzycki J.W., Szczepek W.J., Frelek J. (2007). Practical method for the absolute configuration assignment of tert/tert 1,2-diols using their complexes with Mo_2_(OAc)_4_. J. Org. Chem..

[20] Di Bari L., Pescitelli G., Pratelli C., Pini D., Salvadori P. (2001). Determination of absolute configuration of acyclic 1,2-diols with Mo_2_(OAc)_4_. 1. Snatzke’s method revisited. J. Org. Chem..

[21] Snatzke G., Wagner U., Wolff H.P. (1981). Circulardichroism—LXXV11: Cottonogenic derivatives of chiral bidentate ligands with the complex [Mo_2_(O_2_CCH_3_)_4_]. Tetrahedron.

[22] Bruhn T., Schaumlöffel A., Hemberger Y., Bringmann G. (2013). SpecDis: Quantifying the comparison of calculated and experimental electronic circular dichroism spectra. Chirality.

[23] Cho E., Kwon O.-S., Chung B., Lee J., Sun J., Shin J., Oh K.-B. (2020). Antibacterial activity of chromomycins from a marine-derived *Streptomyces microflavus*. Mar. Drugs.

[24] Vichai V., Kirtikara K. (2006). Sulforhodamine B colorimetric assay for cytotoxicity screening. Nat. Protoc..

[25] Im J.H., Shin Y.-H., Bae E.S., Lee S.K., Oh D.-C. (2023). Jejucarbosides B–E, chlorinated cycloaromatized enediynes, from a marine *Streptomyces* sp.. Mar. Drugs.

[26] Bringmann G., Lang G., Maksimenka K., Hamm A., Gulder T.A.M., Dieter A., Bull A.T., Stach J.E.M., Kocher N., Müller W.E.G. (2005). Gephyromycin, the first bridged angucyclinone, from *Streptomyces griseus* strain NTK 14. Phytochemistry.

[27] Kharel M.K., Pahari P., Shepherd M.D., Tibrewal N., Nybo S.E., Shaaban K.A., Rohr J. (2012). Angucyclines: Biosynthesis, mode-of-action, new natural products, and synthesis. Nat. Prod. Rep..

[28] Wu T., Salim A.A., Cui H., Khalil Z.G., Bernhardt P.V., Capon R.J. (2022). Glenthenamines A–F: Enamine pyranonaphthoquinones from the Australian pasture plant derived *Streptomyces* sp. CMB-PB042. J. Nat. Prod..

[29] Wu C., Du C., Ichinose K., Choi Y.H., van Wezel G.P. (2017). Discovery of *C*-Glycosylpyranonaphthoquinones in *Streptomyces* sp. MBT76 by a combined NMR-based metabolomics and bioinformatics workflow. J. Nat. Prod..

[30] Hong L., Zhao Z., Melançon C.E., Zhang H., Liu H.-W. (2008). In vitro characterization of the enzymes involved in TDP-d-Forosamine biosynthesis in the spinosyn pathway of *Saccharopolyspora spinosa*. J. Am. Chem. Soc..

