# Supplementary material for: Tandocyclinones A and B, Ether Bridged C-Glycosyl Benz[a]anthracenes from an Intertidal Zone Streptomyces sp
Source: Mar Drugs. 2023 Sep 21;21(9):500. doi: 10.3390/md21090500 (PMC10533038; doi:10.3390/md21090500)

Supplementary Materials

Tandocyclinones A and B, Ether Bridged *C*-GlycosylBenz[*a*]anthracenes from an Intertidal Zone *Streptomyces* sp.

Thanh-Hau Huynh [1], Eun Seo Bae [1], Bo Eun Heo [2], Jayho Lee [3], Joon Soo An [1], Yun Kwon [4], Sang-Jip Nam [5], Ki-Bong Oh [3], Jichan Jang [2], Sang Kook Lee [1] and Dong-Chan Oh [1,*]

[1] Natural Products Research Institute, College of Pharmacy, Seoul National University, Seoul 08826, Republic of Korea; 2019-22632@snu.ac.kr (T.-H.H.); ddol1289@snu.ac.kr (E.S.B.); ahnjunsoo@snu.ac.kr (J.S.A.); sklee61@snu.ac.kr (S.K.L.)

[2] Division of Life Science, Department of Bio & Medical Big Data (BK21 Four Program), Research Institute of Life Science, Gyeongsang National University, Jinju 52828, Republic of Korea; hbo0113@naver.com (B.E.H.); jichanjang@gnu.ac.kr (J.J.)

[3] Department of Agricultural Biotechnology, College of Agriculture and Life Sciences and Natural Products Research Institute, Seoul National University, Seoul 08826, Republic of Korea; jayho@snu.ac.kr (J.L.); ohkibong@snu.ac.kr (K.-B.O.)

[4] Research Institute of Pharmaceutical Sciences, College of Pharmacy, Kyungpook National University, Daegu 41566, Republic of Korea; yunkwon@knu.ac.kr

[5] Department of Chemistry and Nanoscience, Ewha Womans University, Seoul 03760, Republic of Korea; sjnam@ewha.ac.kr

[*] Correspondence: dongchanoh@snu.ac.kr; Tel.: +82-880-2491; Fax: +82-762-8322

Table of contents

S3 **Figure S1.** Comparison of ^{1}H NMR spectra of tandocyclinone A (**1**) in various solvents (pyridine-*d*$_5$, acetone-*d*$_6$, DMSO-*d*$_6$, CD_3CN, THF-*d*$_8$, and CD_3OD)

S4 **Figure S2.** ^{1}H NMR spectrum of tandocyclinone A (**1**) at 800 MHz in THF-*d*$_8$

S5 **Figure S3.** ^{13}C NMR spectrum of tandocyclinone A (**1**) at 200 MHz in THF-*d*$_8$

S6 **Figure S4.** COSY NMR spectrum of tandocyclinone A (**1**) at 800 MHz in THF-*d*$_8$

S6 **Figure S5.** HSQC NMR spectrum of tandocyclinone A (**1**) at 800 MHz in THF-*d*$_8$

S7 **Figure S6.** ^{1}H–^{15}N HSQC NMR spectrum of tandocyclinone A (**1**) at 800 MHz in THF-*d*$_8$

S7 **Figure S7.** HMBC NMR spectrum of tandocyclinone A (**1**) at 800 MHz in THF-*d*$_8$

S8 **Figure S8.** ROESY NMR spectrum of tandocyclinone A (**1**) at 800 MHz in THF-*d*$_8$

S9 **Figure S9.** ^{1}H NMR spectrum of tandocyclinone B (**2**) at 800 MHz in THF-*d*$_8$

S10 **Figure S10.** ^{13}C NMR spectrum of tandocyclinone B (**2**) at 200 MHz in THF-*d*$_8$

S11 **Figure S11.** COSY NMR spectrum of tandocyclinone B (**2**) at 800 MHz in THF-*d*$_8$

S11 **Figure S12**. HSQC NMR spectrum of tandocyclinone B (**2**) at 800 MHz in THF-*d*$_8$

S12 **Figure S13.** HMBC NMR spectrum of tandocyclinone B (**2**) at 800 MHz in THF-*d*$_8$

S12 **Figure S14.** ROESY NMR spectrum of tandocyclinone B (**2**) at 800 MHz in THF-*d*$_8$

S13 **Figure S15.** ^{1}H NMR spectrum of tandocyclinone B (**2**) at 400 MHz in CD_3OD

S14 **Figure S16.** ^{1}H NMR spectrum of acetylation product of tandocyclinone B (**2**) at 400 MHz in CD_3OD

S15 **Figure S17.** ^{1}H NMR spectrum of tandocyclinone A (**1**) at 400 MHz in CD_3OD

S16 **Figure S18.** Comparison of ^{1}H NMR spectra of **2,** acetylation product of **2,** and **1** in CD_3OD

S17 **Figure S19.** ^{1}H NMR spectrum of (*S*)-MTPA amide (**2a**) of tandocyclinone B (**2**) at 800 MHz in CD_3OD

S18 **Figure S20.** COSY NMR spectrum of (*S*)-MTPA amide (**2a**) of tandocyclinone B (**2**) at 800 MHz in CD_3OD

S18 **Figure S21.** HSQC NMR spectrum of (*S*)-MTPA amide (**2a**) of tandocyclinone B (**2**) at 800 MHz in CD_3OD

S19 **Figure S22.** HMBC NMR spectrum of (*S*)-MTPA amide (**2a**) of tandocyclinone B (**2**) at 800 MHz in CD_3OD

S20 **Figure S23.** ^{1}H NMR spectrum of (*R*)-MTPA amide (**2b**) of tandocyclinone B (**2**) at 800 MHz in CD_3OD

S21 **Figure S24.** COSY NMR spectrum of (*R*)-MTPA amide (**2b**) of tandocyclinone B (**2**) at 800 MHz in CD_3OD

S21 **Figure S25.** HSQC NMR spectrum of (*R*)-MTPA amide (**2b**) of tandocyclinone B (**2**) at 800 MHz in CD_3OD

S22 **Figure S26.** HMBC NMR spectrum of (*R*)-MTPA amide (**2b**) of tandocyclinone B (**2**) at 800 MHz in CD_3OD

S23 **Figure S27.** Liquid chromatography profiles of tandocyclinone B (**2**), acetylation product of **2**, and tandocyclinone A (**1**)

S23 **Figure S28.** CD spectra for tandocyclinones A (**1**, red), and B (**2**, blue)

S24 **Figure S29**. IR spectrum of tandocyclinone A (**1**)

S25 **Figure S30**. IR spectrum of tandocyclinone B (**2**)

S26 **Figure S31.** HR-ESI-MS data of tandocyclinone A (**1**)

S26 **Figure S32**. HR-ESI-MS data of tandocyclinone B (**2**)
S27 **Figure S33.** HR-ESI-MS data of (*S*)-MTPA amide (**2a**) of tandocyclinone B (**2**)
S27 **Figure S34.** HR-ESI-MS data of (*R*)-MTPA amide (**2b**) of tandocyclinone B (**2**)
S28 **Figure S35.** The simulated models of two possible diastereomers **1A** (4*S*,4a*R*,6a*S*,12a*S*,12b*S*,1′*R*,4′*S*,5′*R*) and **1B** (4*R*,4a*S*,6a*R*,12a*R*,12b*R*,1′*R*,4′*S*,5′*R*) of tandocyclinone A (**1**)
S28 **Table S1.** Energy analysis for diastereomers **1A** (4*S*,4a*R*,6a*S*,12a*S*,12b*S*,1′*R*,4′*S*,5′*R*) and **1B** (4*R*,4a*S*,6a*R*,12a*R*,12b*R*,1′*R*,4′*S*,5′*R*) of tandocyclinone A (**1**)
S29 **Table S2.** ECD calculation of conformer 1 of diastereomer **1A**
S30 **Table S3.** ECD calculation of conformer 2 of diastereomer **1A**
S31 **Table S4.** ECD calculation of conformer 3 of diastereomer **1A**
S32 **Table S5.** ECD calculation of conformer 1 of diastereomer **1B**
S33 **Table S6.** ECD calculation of conformer 2 of diastereomer **2B**
S34 **Table S7.** ECD calculation of conformer 3 of diastereomer **2B**
S35 **Figure S36.** The proposed polyketide cyclization patterns of (a) tandocyclinones and (b) angucyclinones
S36 Proposed biosynthetic pathways of tandocyclinones A and B (**1** and **2**)
S36 **Scheme S1**. Proposed biosynthetic pathways of tandocyclinones A and B (**1** and **2**)
S37 **Figure S37.** ^{1}H NMR (600 MHz, DMSO-d_6) and UV-vis (inset) spectra of BE-54238A (mass 397) from reference
S37 **Figure S38.** ^{1}H NMR (800 MHz, DMSO-d_6), UV-vis (inset) and MS (positive mode, inset) spectra of TDH03.397

Figure S1. Comparison of ^{1}H NMR spectra of tandocyclinone A (**1**) in various solvents (pyridine-*d*5, acetone-*d*6, DMSO-*d*6, CD_3CN, THF-*d*8, and CD_3OD)

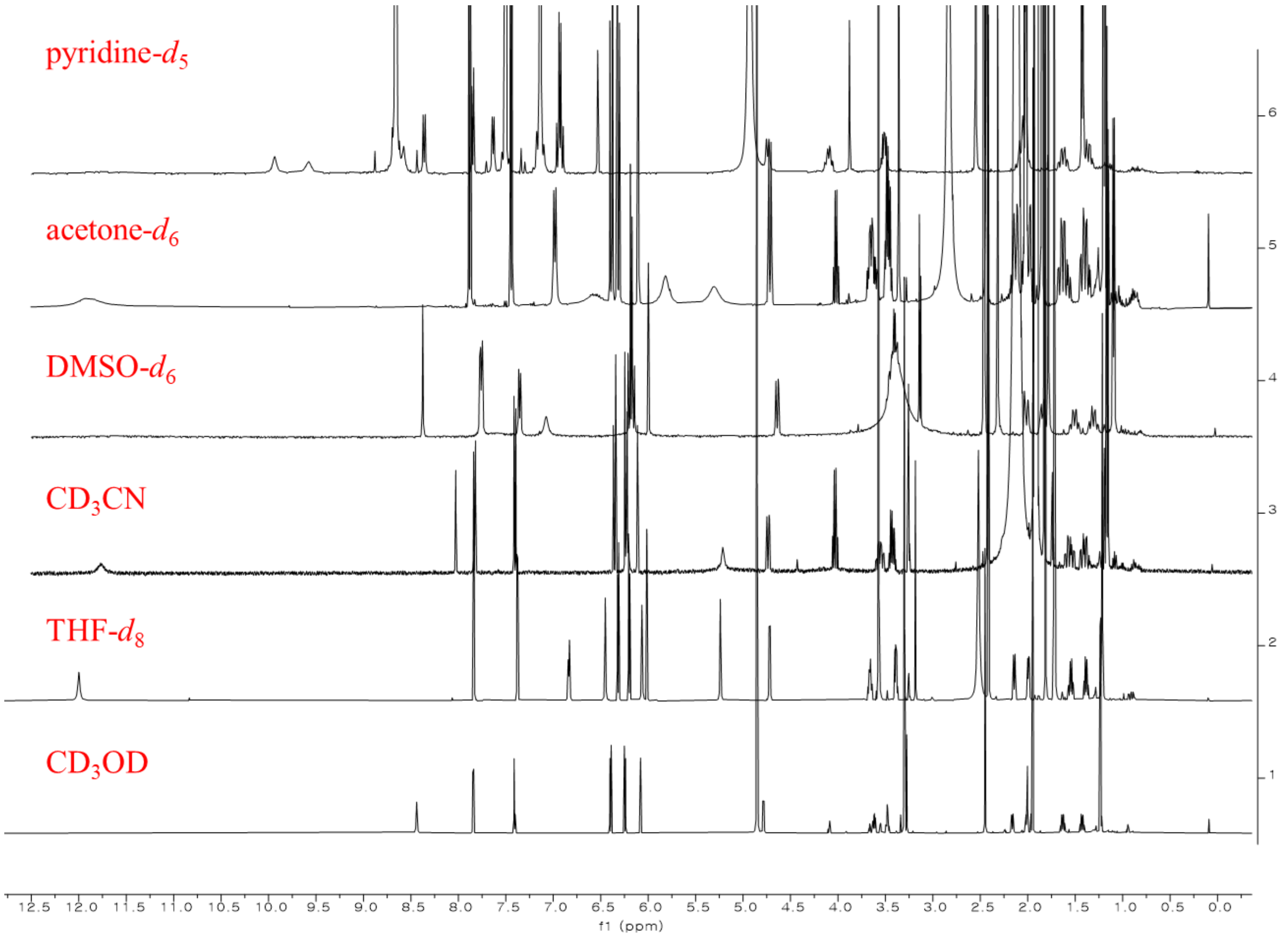

Figure S2. ^{1}H NMR spectrum of tandocyclinone A (**1**) at 800 MHz in THF-d_8.

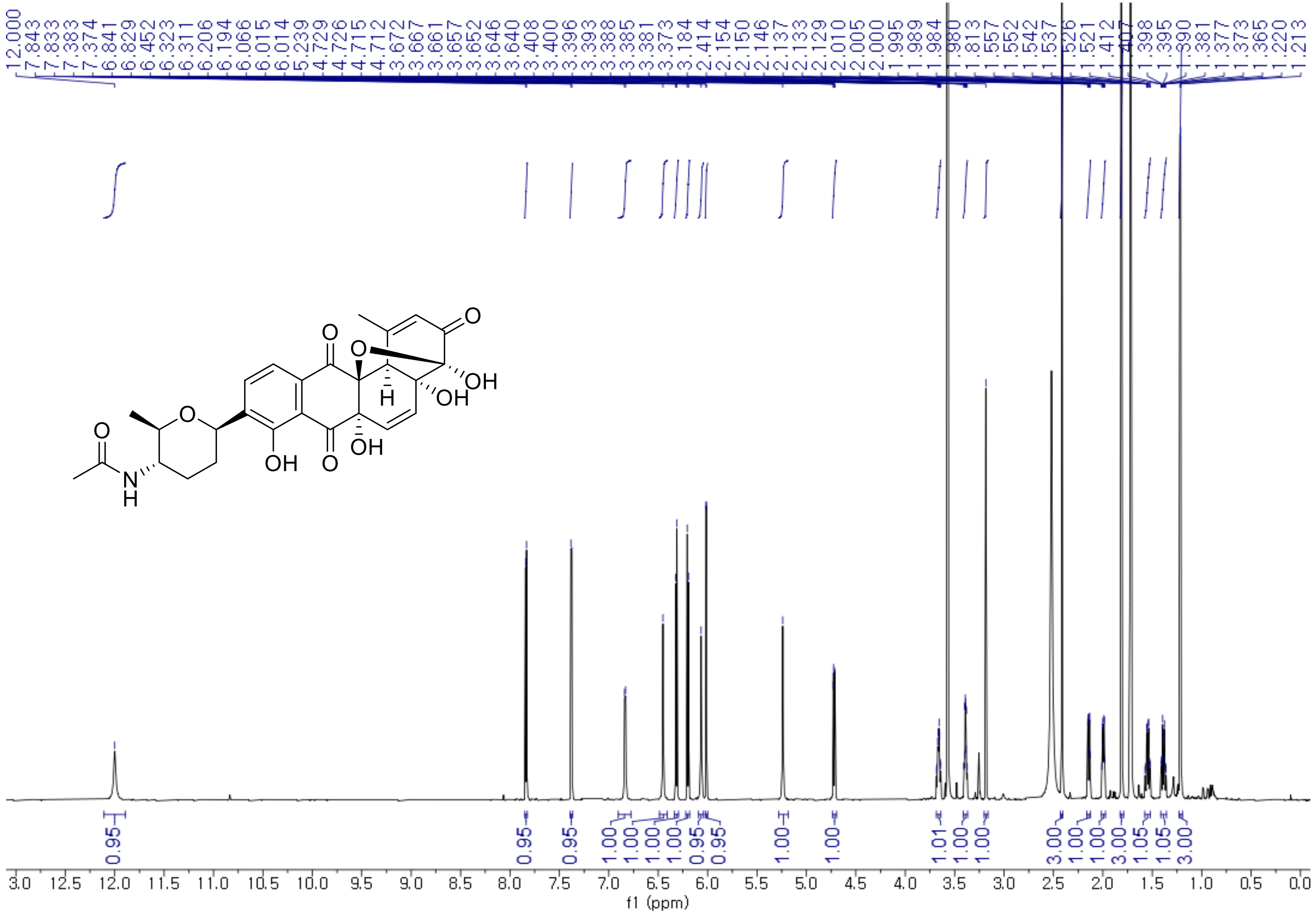

Figure S3. ^{13}C NMR spectrum of tandocyclinone A (**1**) at 200 MHz in THF-d_8.

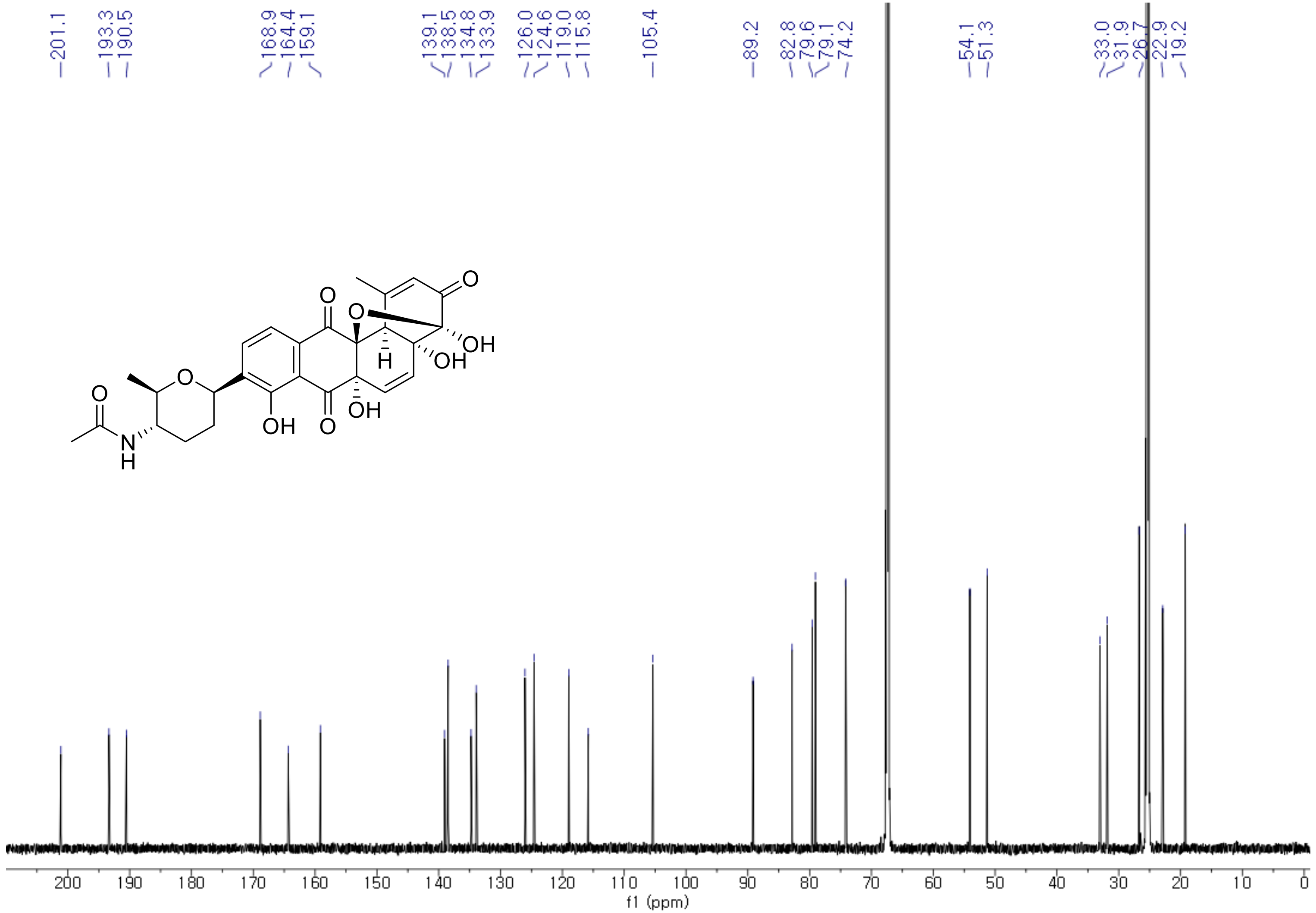

Figure S4. COSY NMR spectrum of tandocyclinone A (**1**) at 800 MHz in THF-d_8.

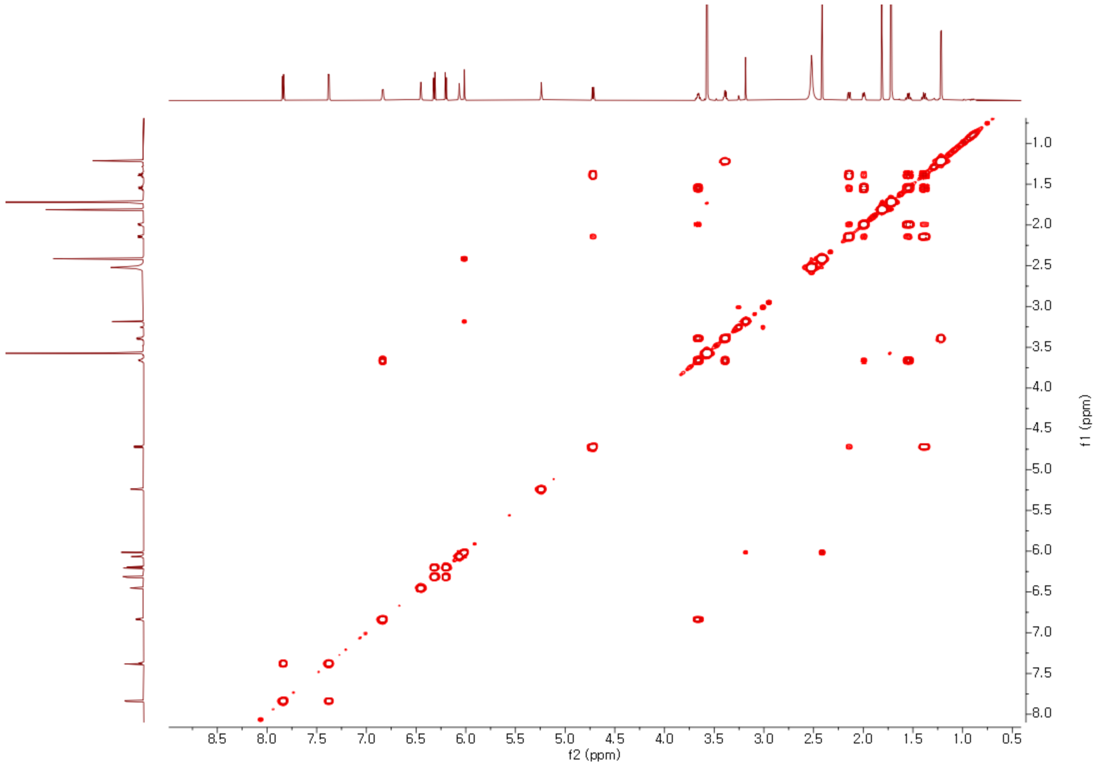

Figure S5. HSQC NMR spectrum of tandocyclinone A (**1**) at 800 MHz in THF-d_8.

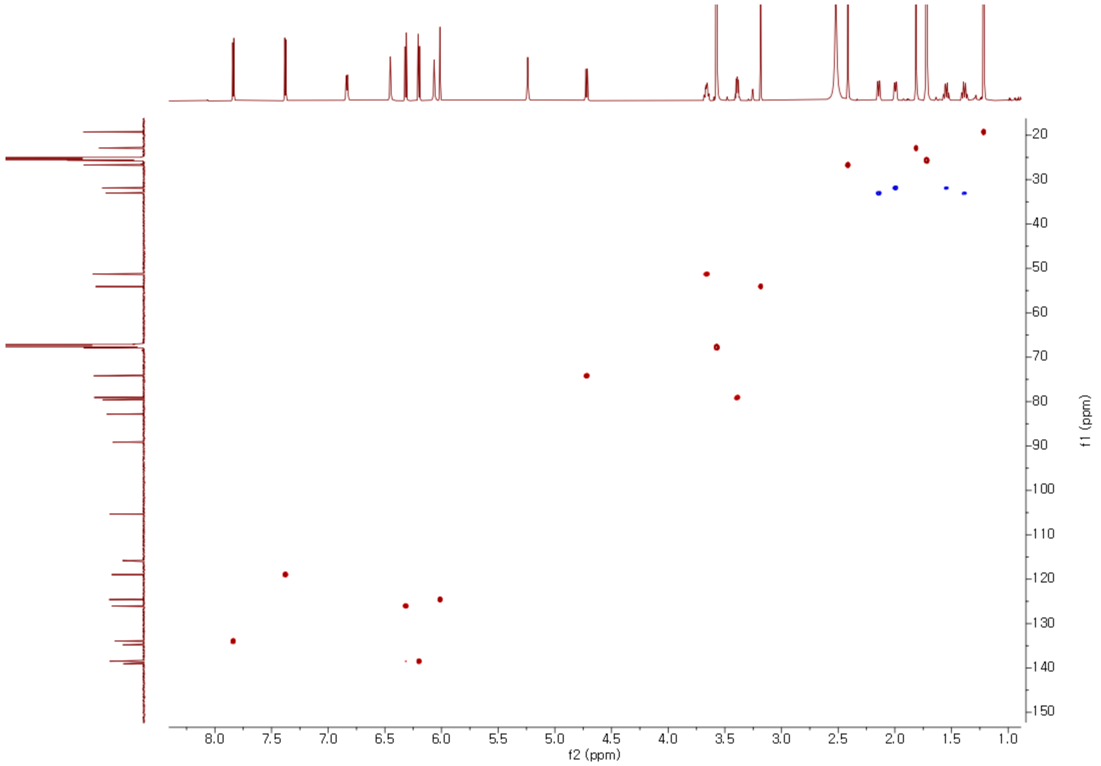

Figure S6. ^{1}H–^{15}N HSQC NMR spectrum of tandocyclinone A (**1**) at 800 MHz in THF-d_8.

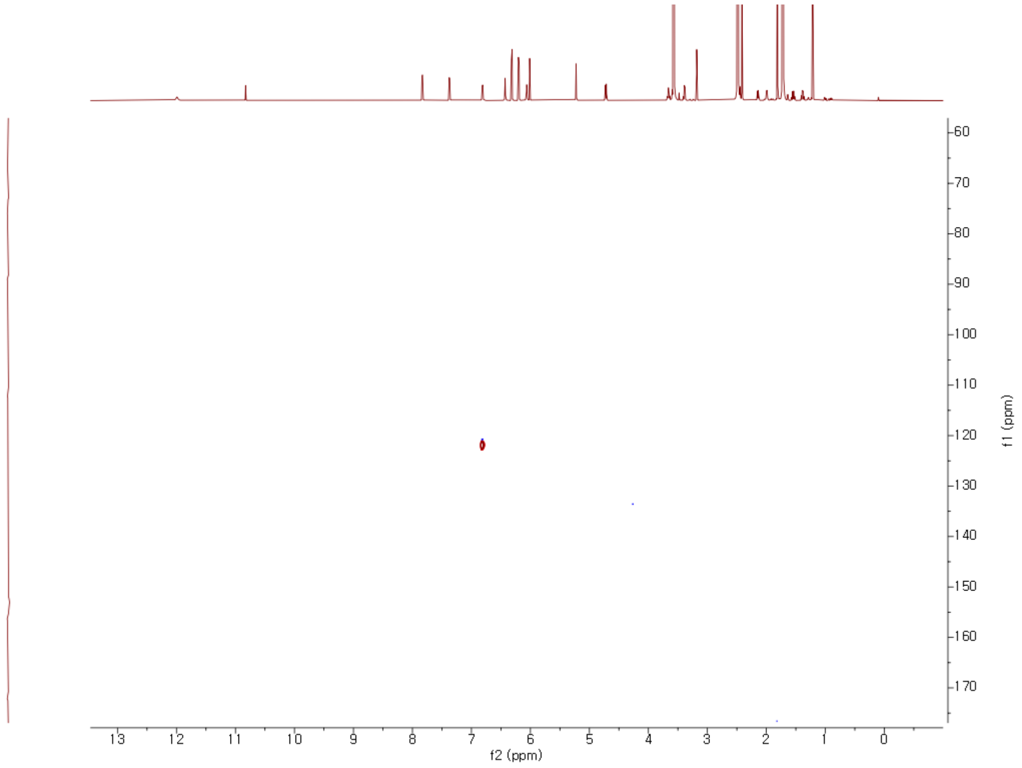

Figure S7. HMBC NMR spectrum of tandocyclinone A (**1**) at 800 MHz in THF-d_8.

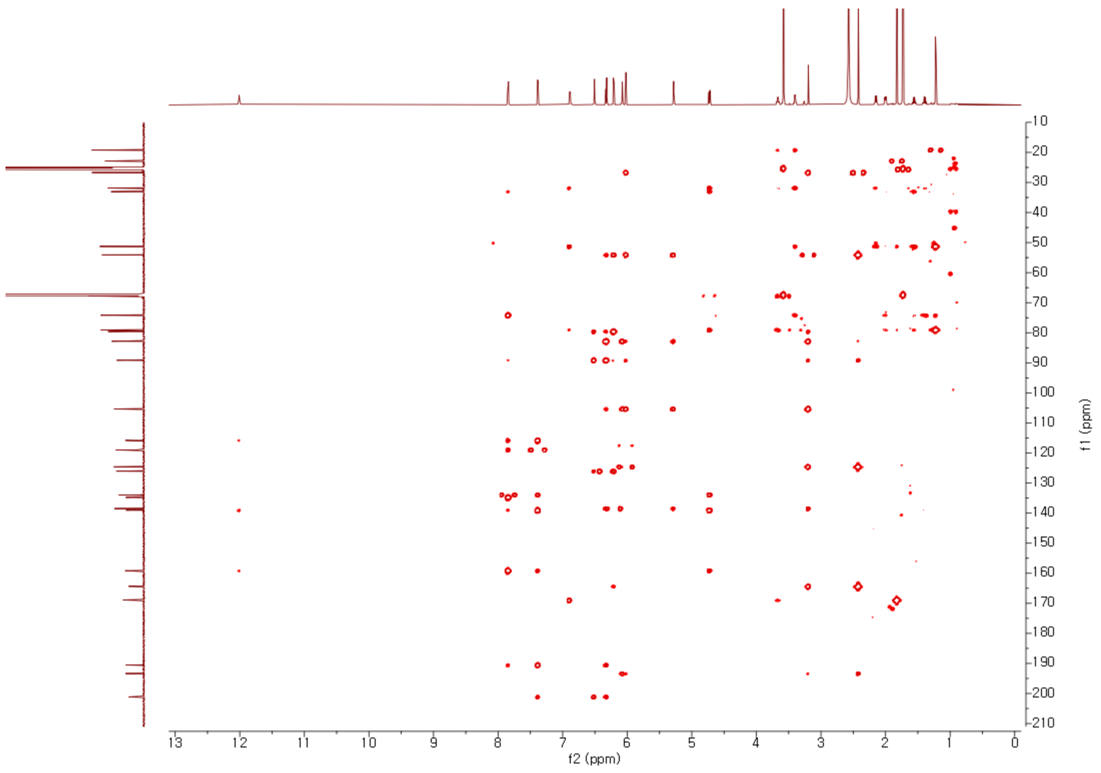

Figure S8. ROESY NMR spectrum of tandocyclinone A (**1**) at 800 MHz in THF-d_8.

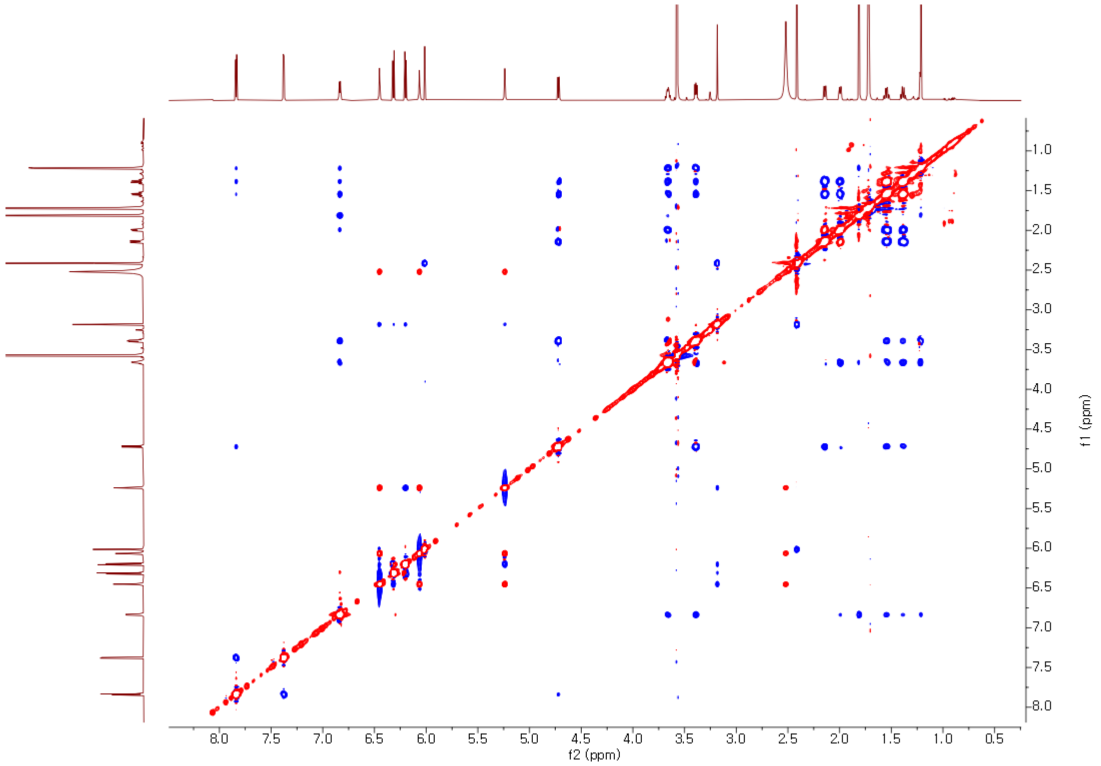

Figure S9. ^{1}H NMR spectrum of tandocyclinone B (**2**) at 800 MHz in THF-d_8.

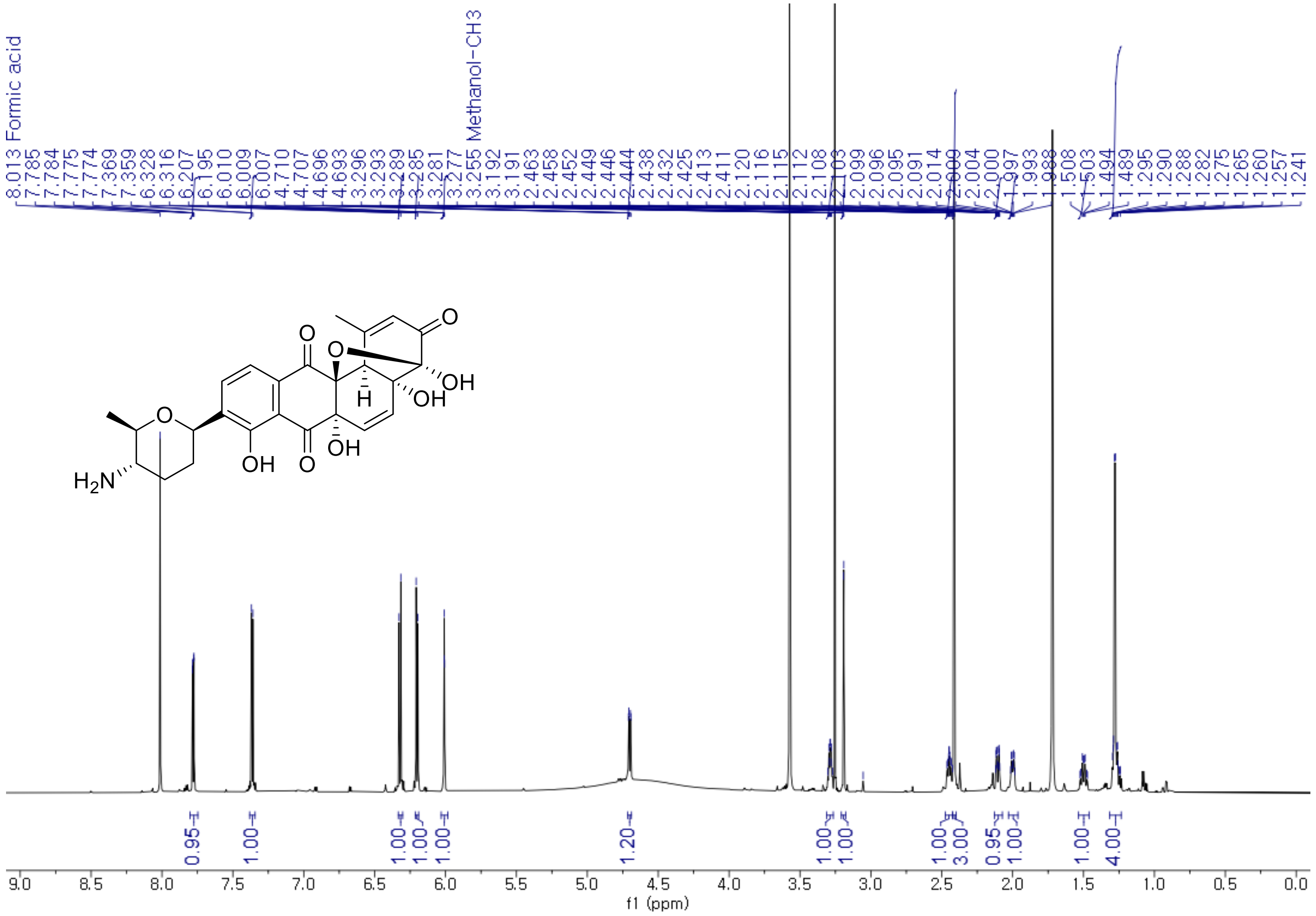

Figure S10. ^{13}C NMR spectrum of tandocyclinone B (**2**) at 200 MHz in THF-d_8.

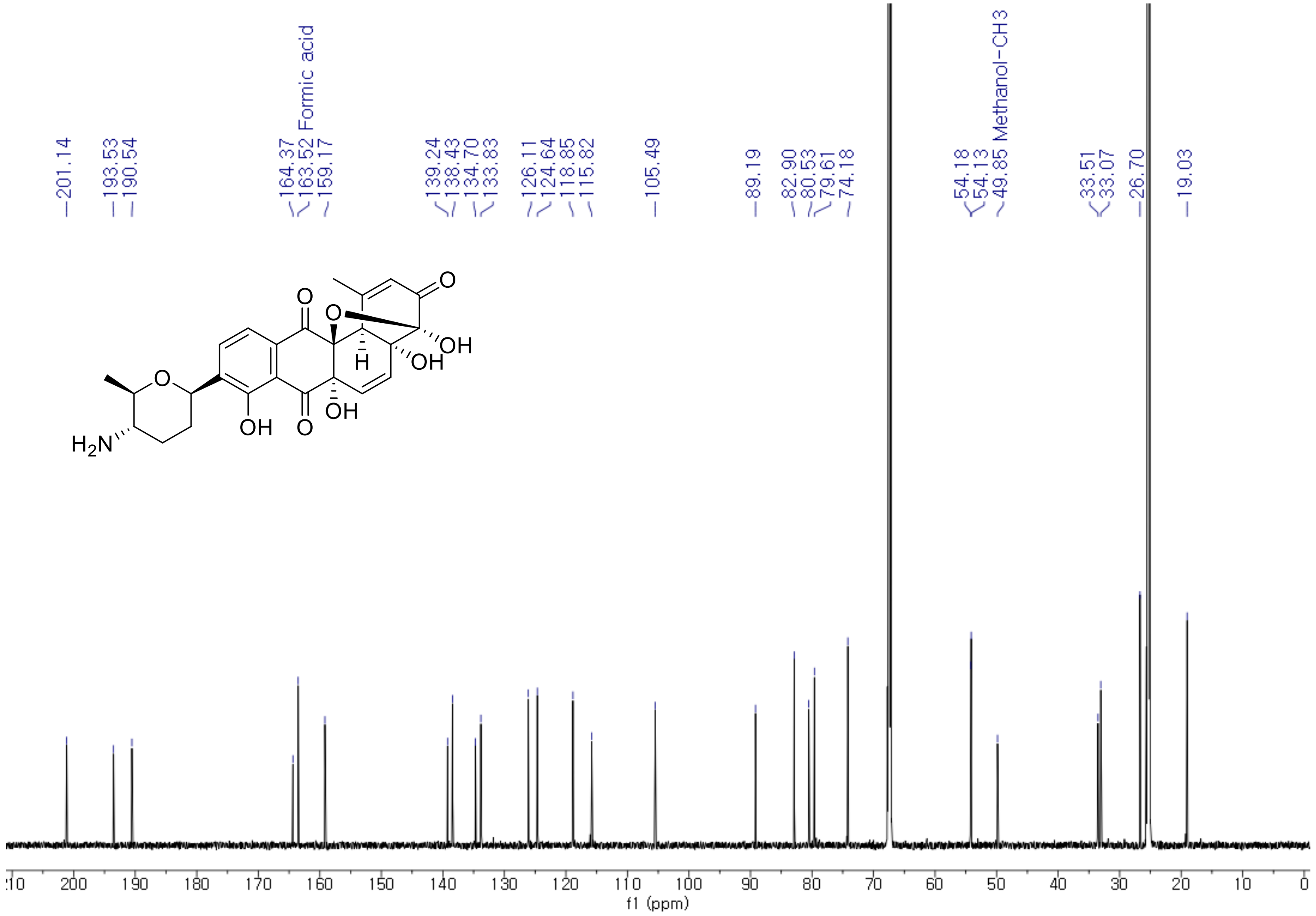

Figure S11. COSY NMR spectrum of tandocyclinone B (**2**) at 800 MHz in THF-d_8.

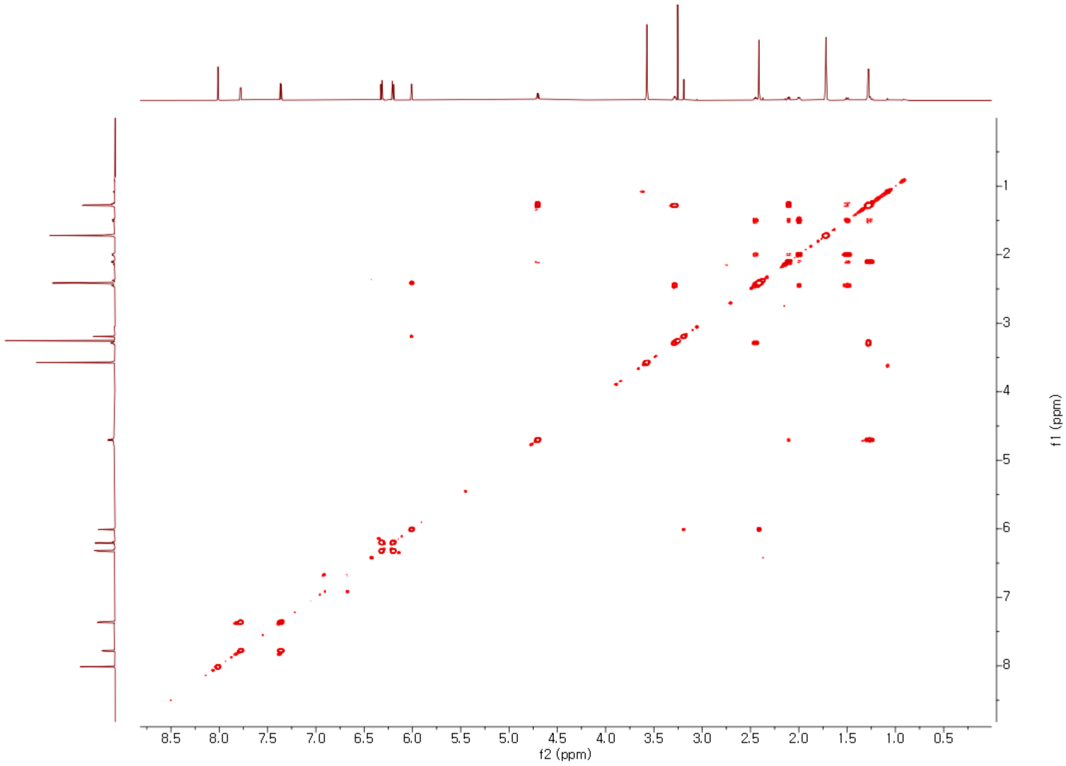

Figure S12. HSQC NMR spectrum of tandocyclinone B (**2**) at 800 MHz in THF-d_8.

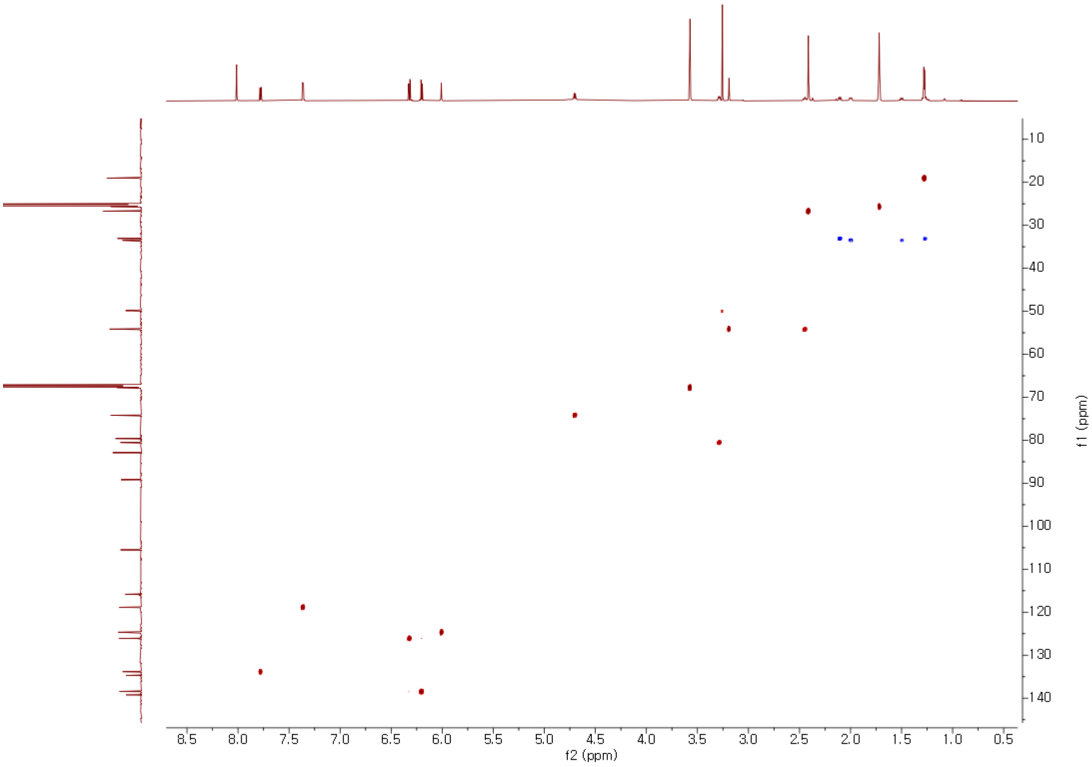

Figure S13. HMBC NMR spectrum of tandocyclinone B (**2**) at 800 MHz in THF-d_8.

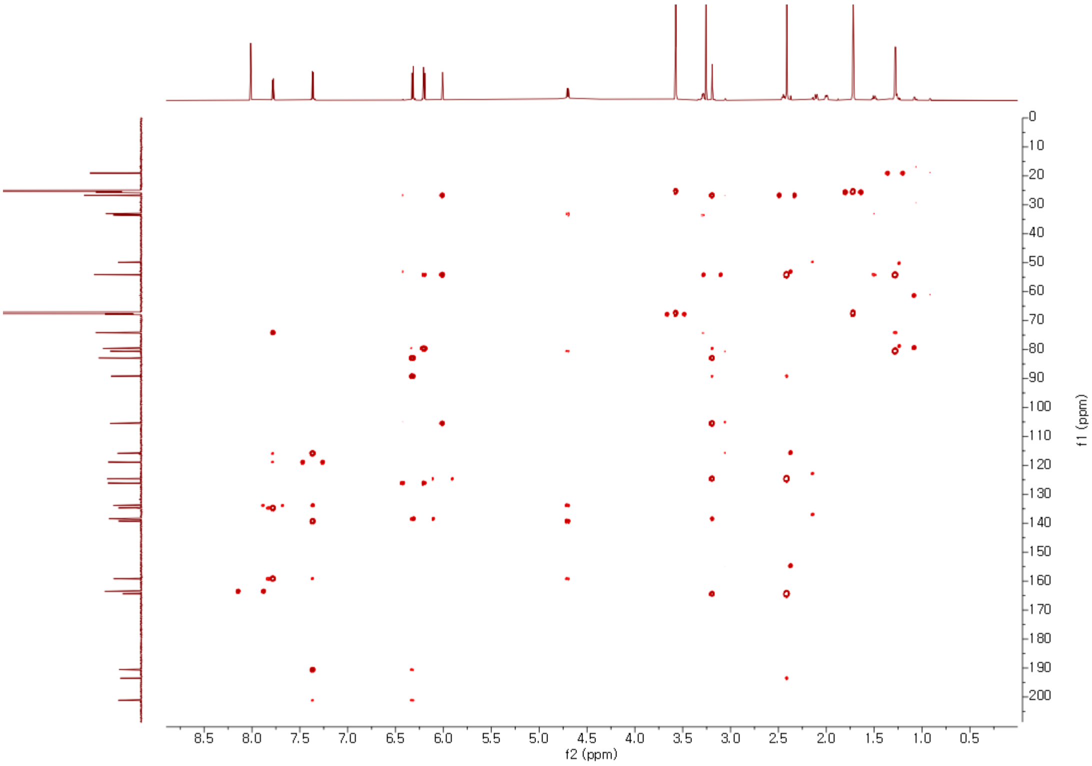

Figure S14. ROESY NMR spectrum of tandocyclinone B (**2**) at 800 MHz in THF-d_8.

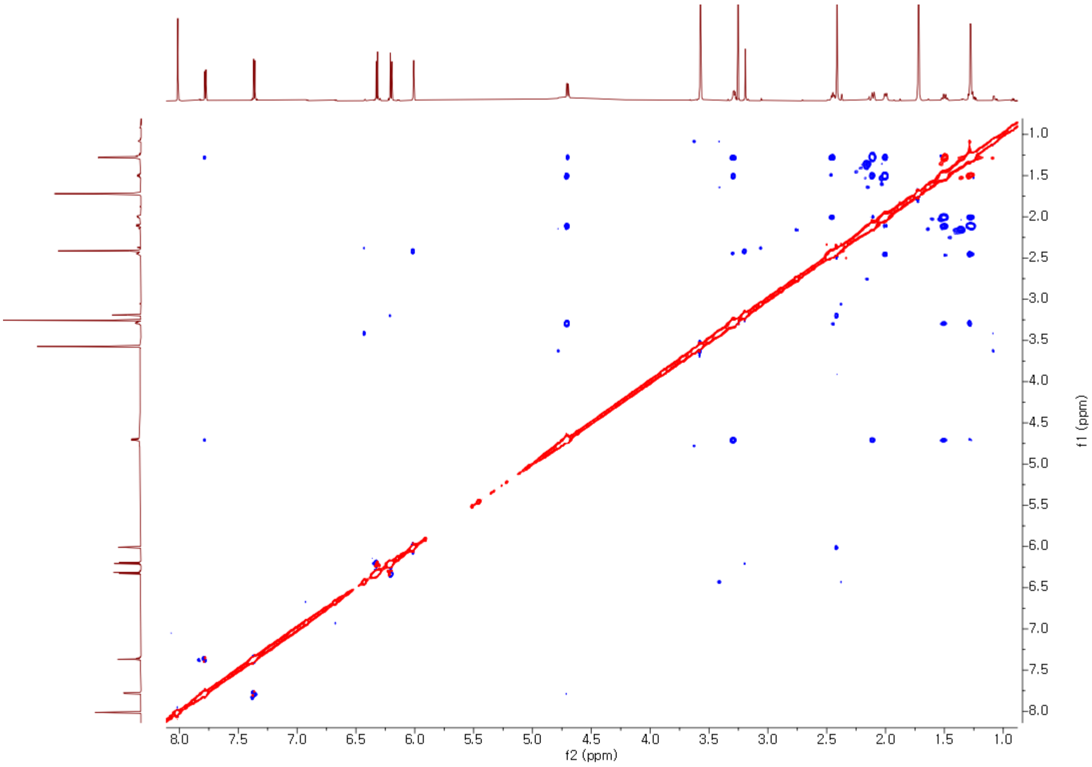

Figure S15. ^{1}H NMR spectrum of tandocyclinone B (**2**) at 400 MHz in CD_3OD.

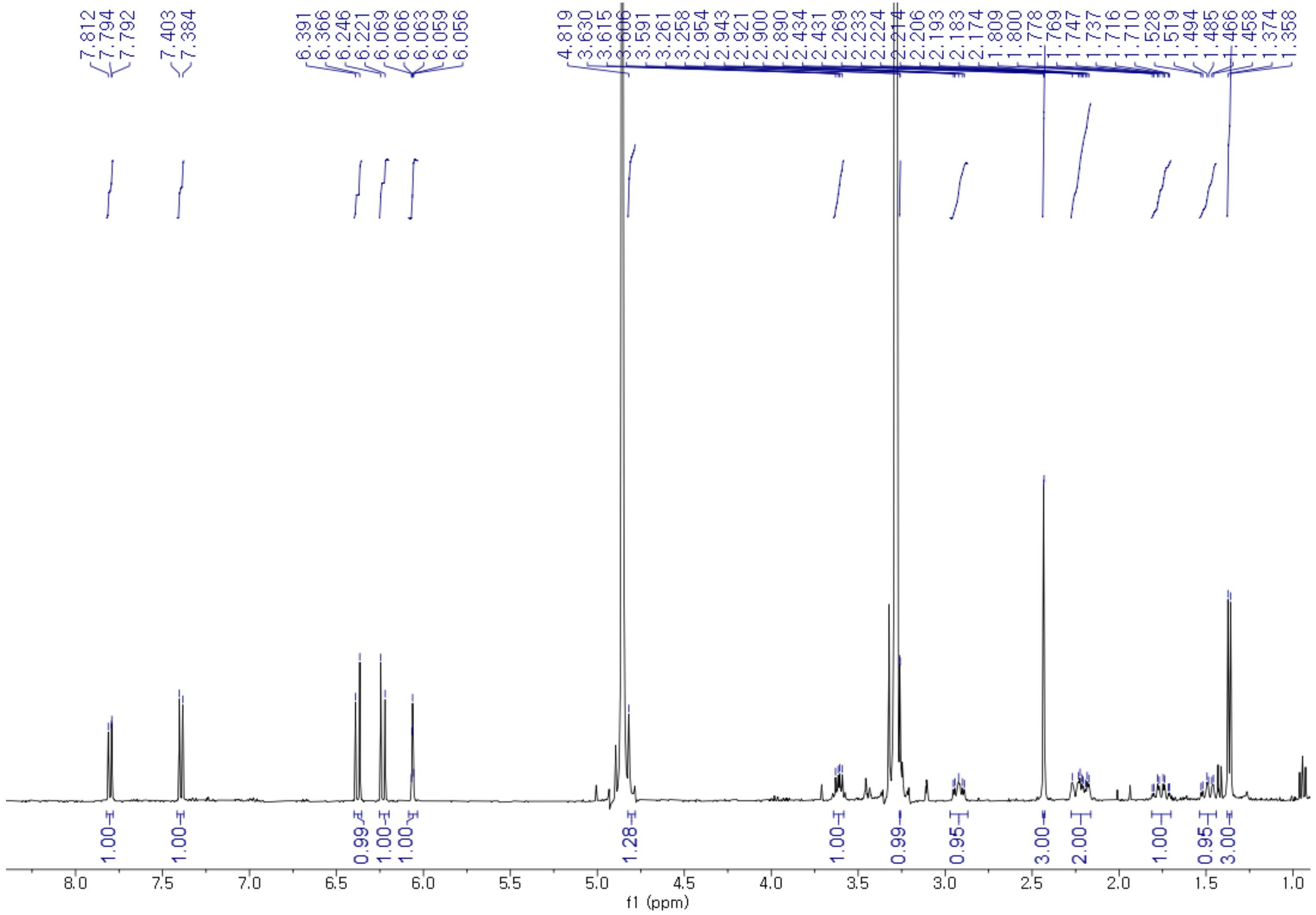

Figure S16. ^{1}H NMR spectrum of acetylation product of tandocyclinone B (**2**) at 400 MHz in CD_3OD.

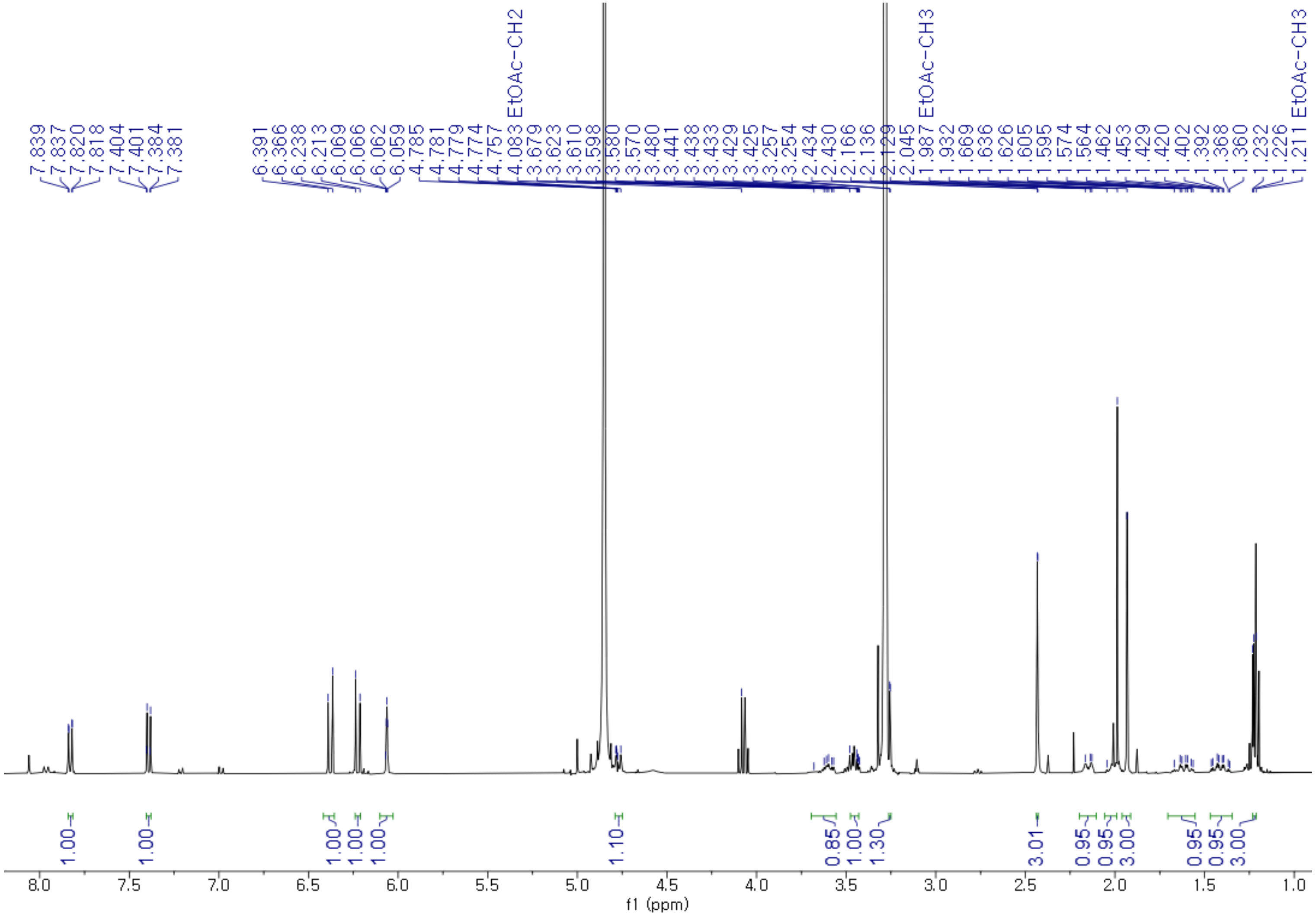

Figure S17. ^{1}H NMR spectrum of tandocyclinone A (**1**) at 400 MHz in CD_3OD.

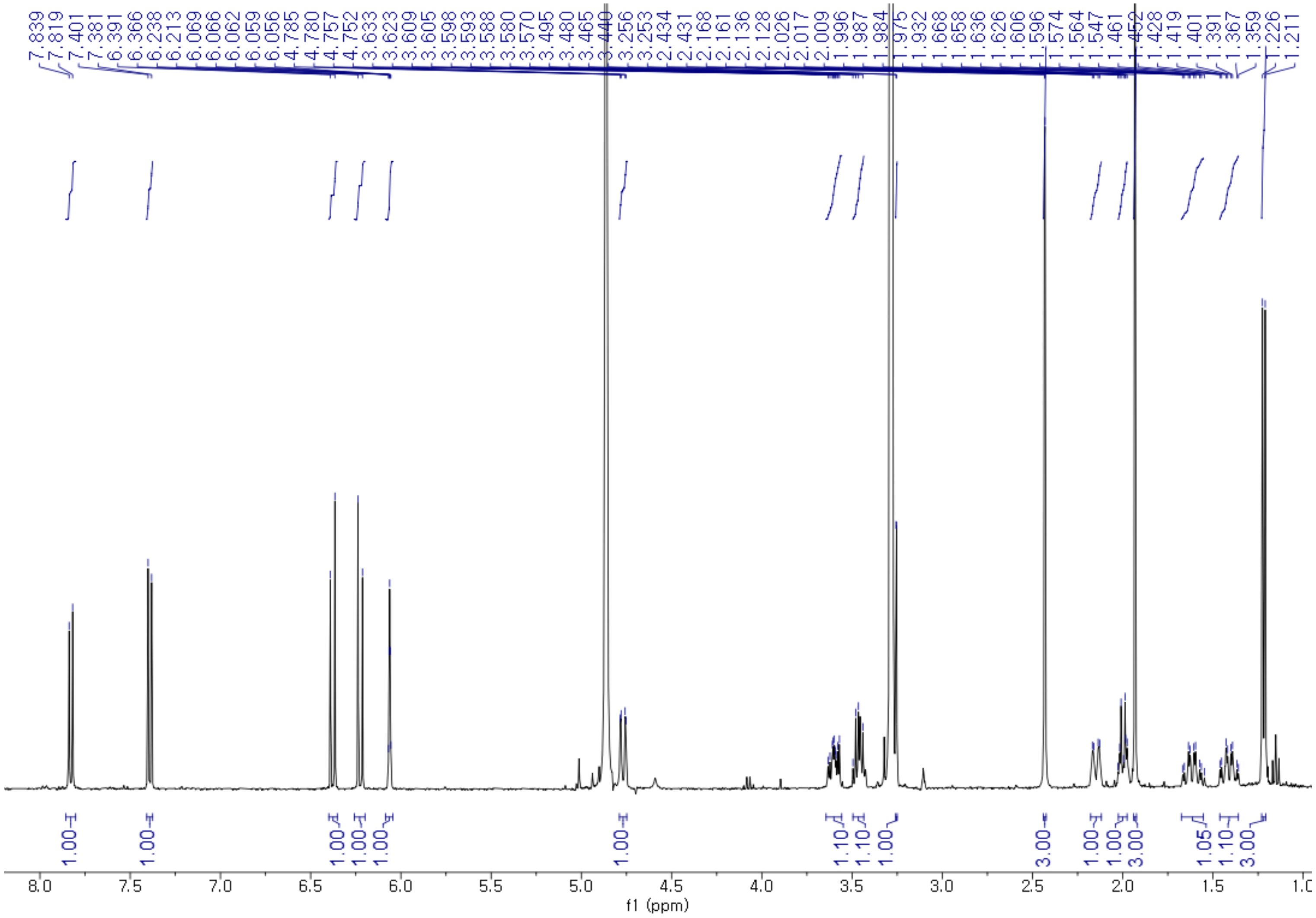

Figure S18. Comparison of ^1H NMR spectra of **2,** acetylation product of **2** and **1** in CD_3OD.

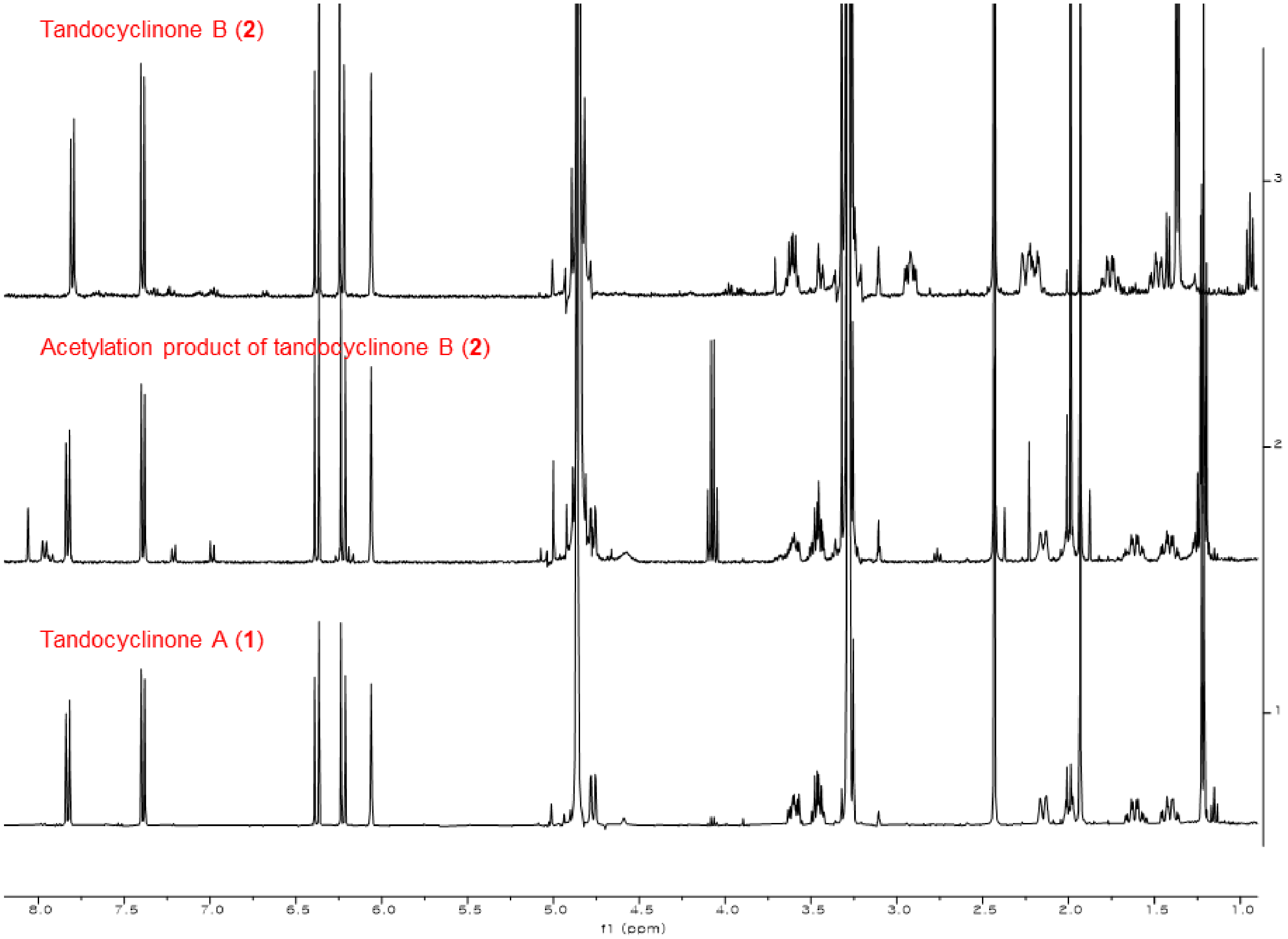

Figure S19. ^{1}H NMR spectrum of (*S*)-MTPA amide (**2a**) of tandocyclinone B (**2**) at 800 MHz in CD_3OD.

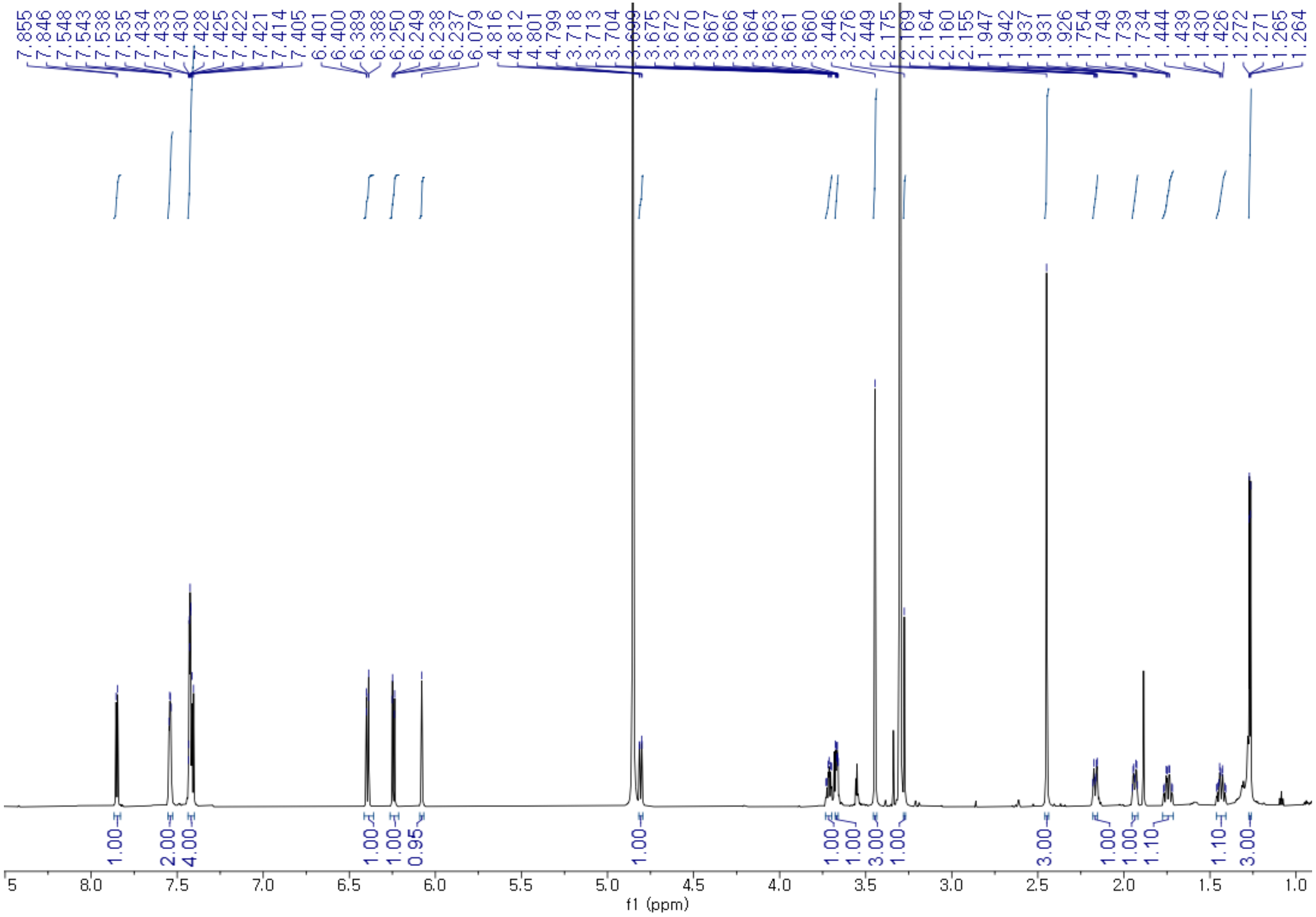

Figure S20. COSY NMR spectrum of (*S*)-MTPA amide (**2a**) of tandocyclinone B (**2**) at 800 MHz in CD_3OD.

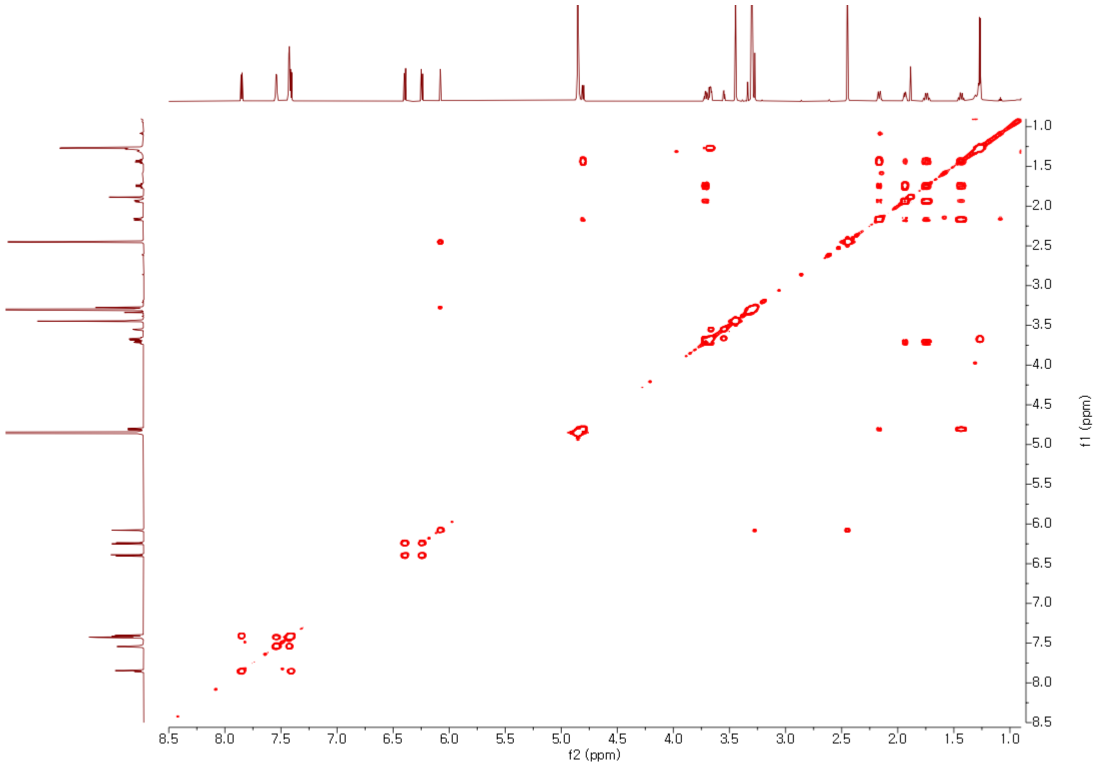

Figure S21. HSQC NMR spectrum of (*S*)-MTPA amide (**2a**) of tandocyclinone B (**2**) at 800 MHz in CD_3OD.

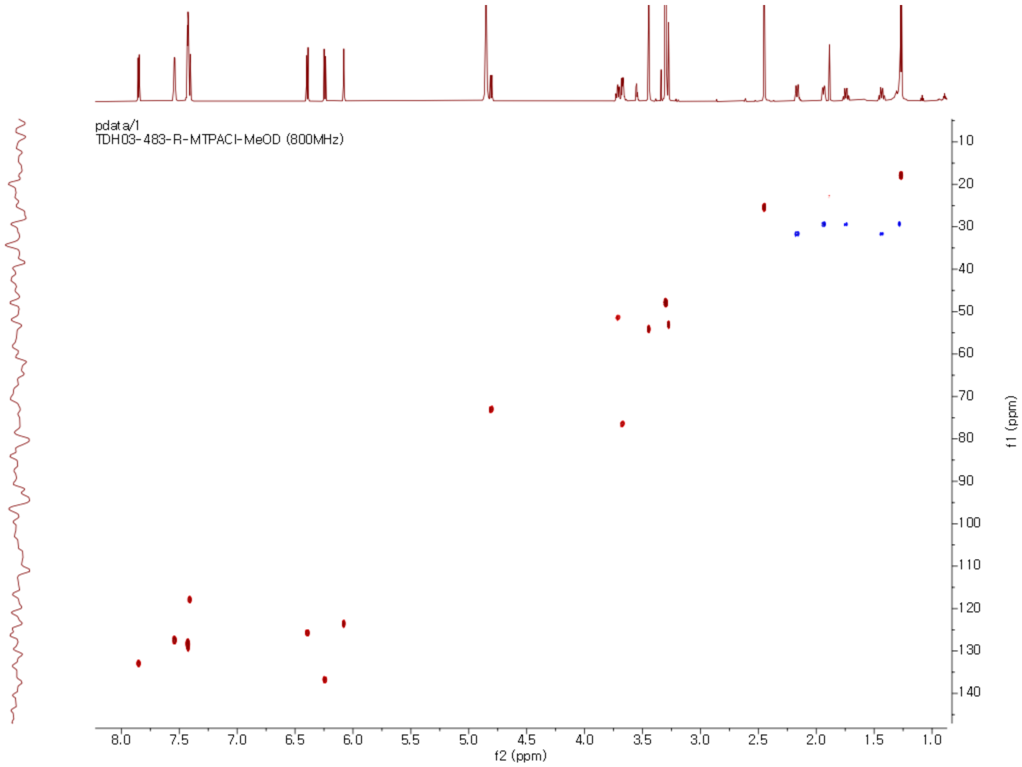

Figure S22. HMBC NMR spectrum of (*S*)-MTPA amide (**2a**) of tandocyclinone B (**2**) at 800 MHz in CD_3OD.

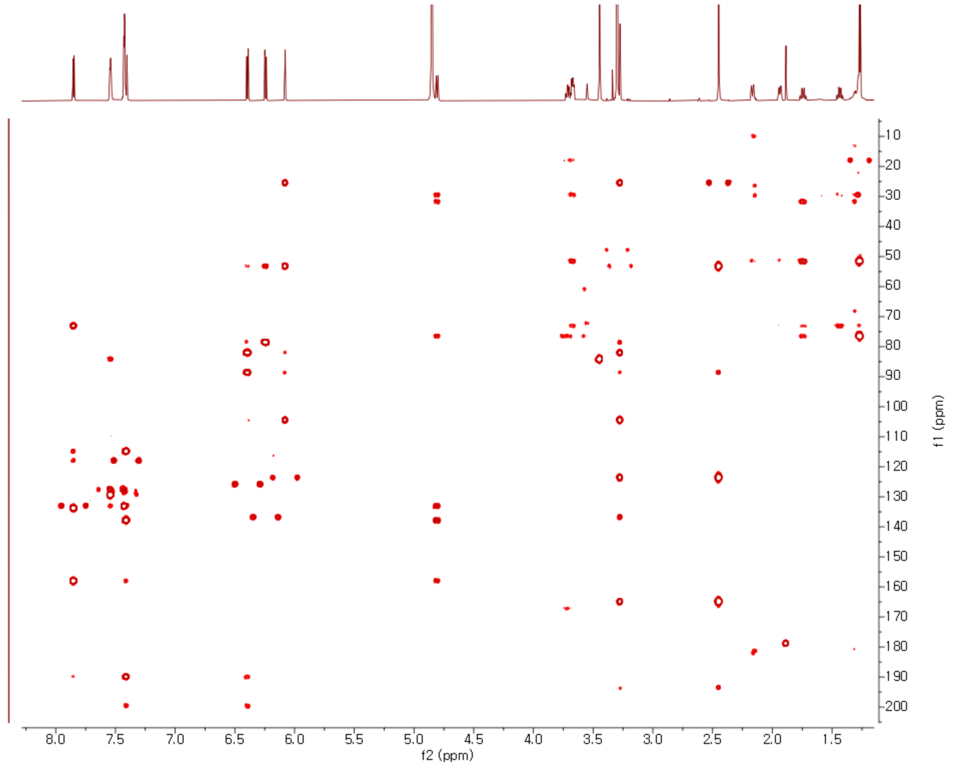

Figure S23. ^{1}H NMR spectrum of (*R*)-MTPA amide (**2b**) of tandocyclinone B (**2**) at 800 MHz in CD_3OD.

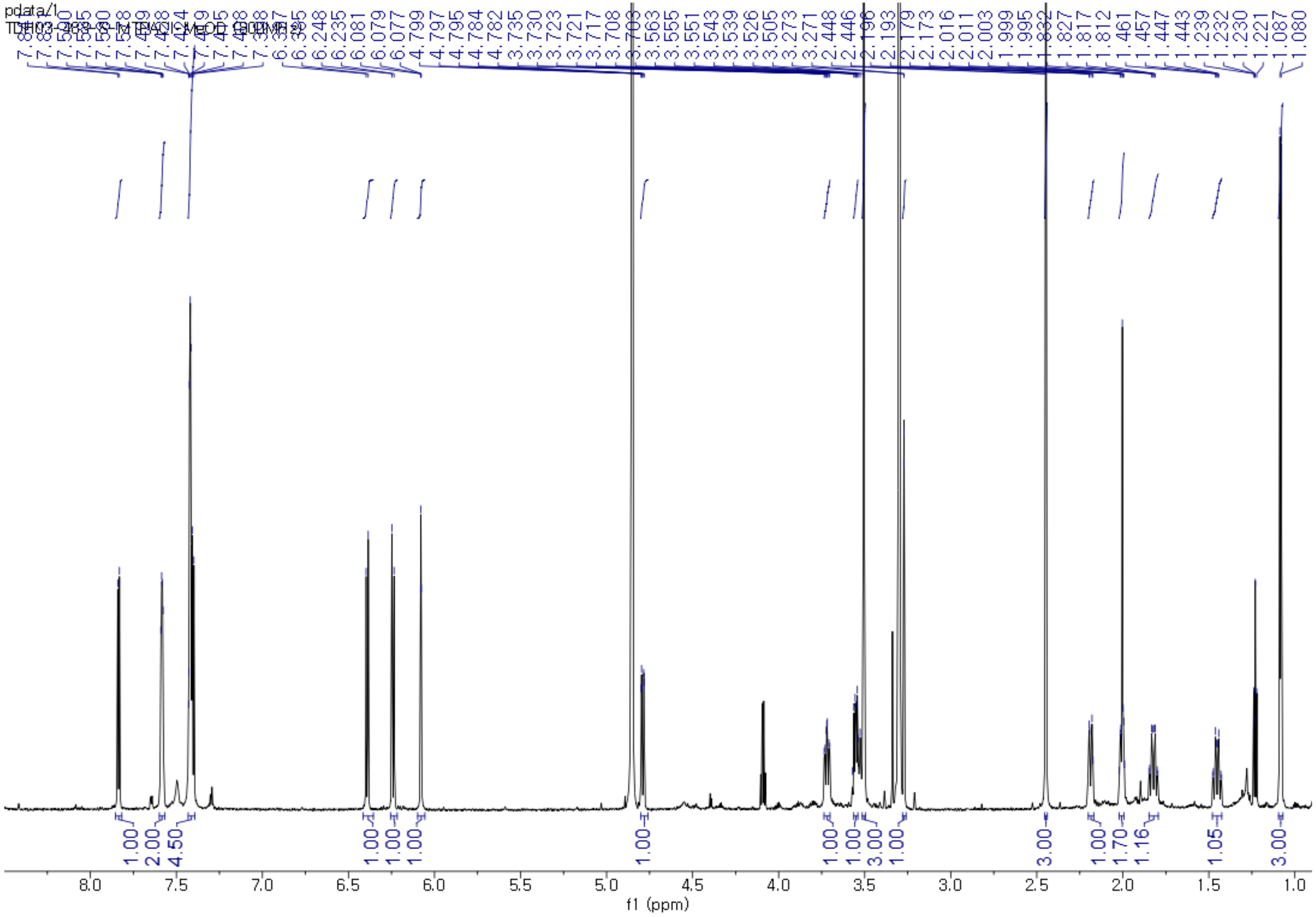

Figure S24. COSY NMR spectrum of (*R*)-MTPA amide (**2b**) of tandocyclinone B (**2**) at 800 MHz in CD_3OD.

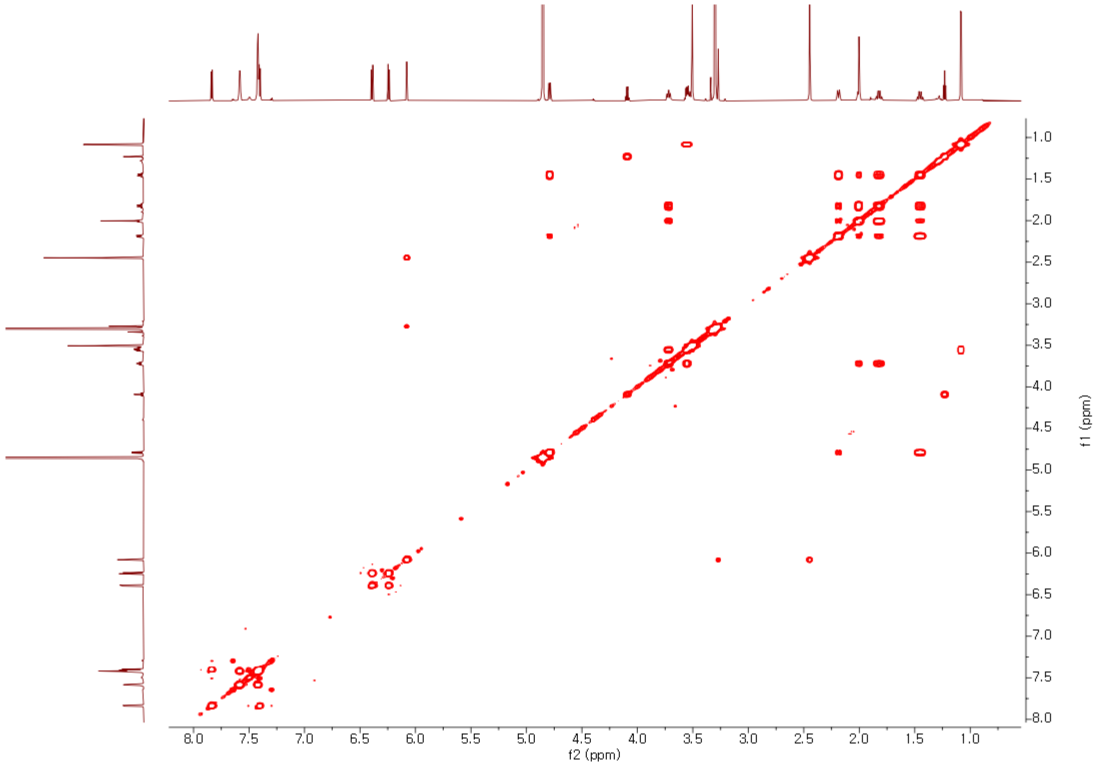

Figure S25. HSQC NMR spectrum of (*R*)-MTPA amide (**2b**) of tandocyclinone B (**2**) at 800 MHz in CD_3OD.

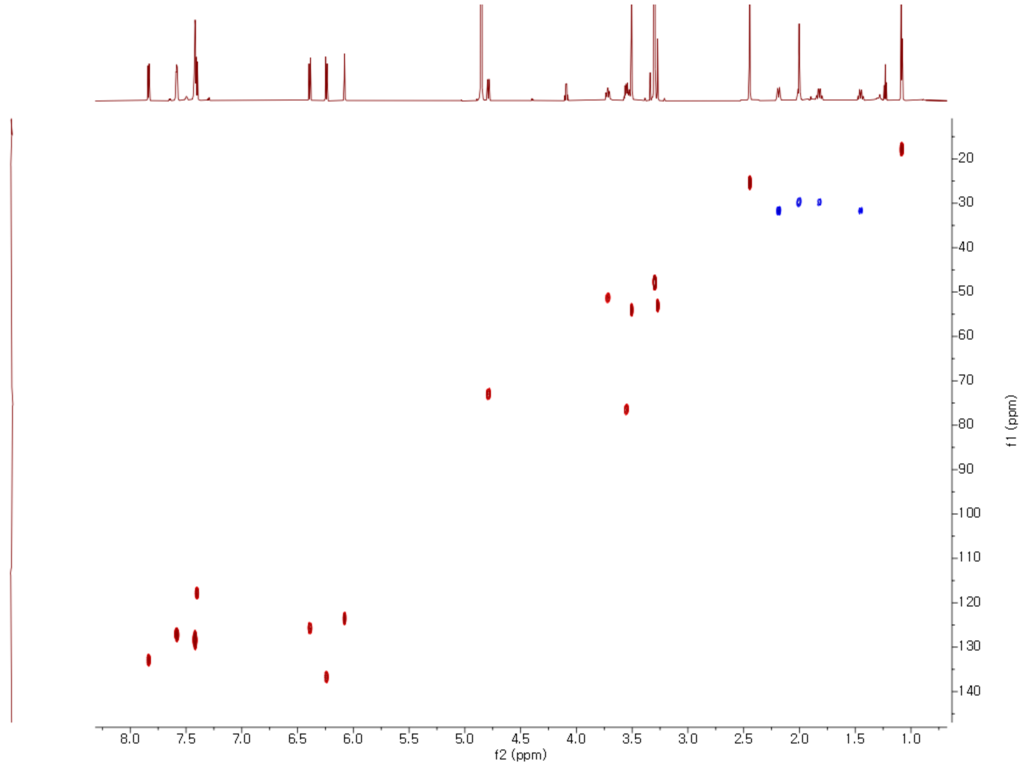

Figure S26. HMBC NMR spectrum of (*R*)-MTPA amide (**2b**) of tandocyclinone B (**2**) at 800 MHz in CD_3OD.

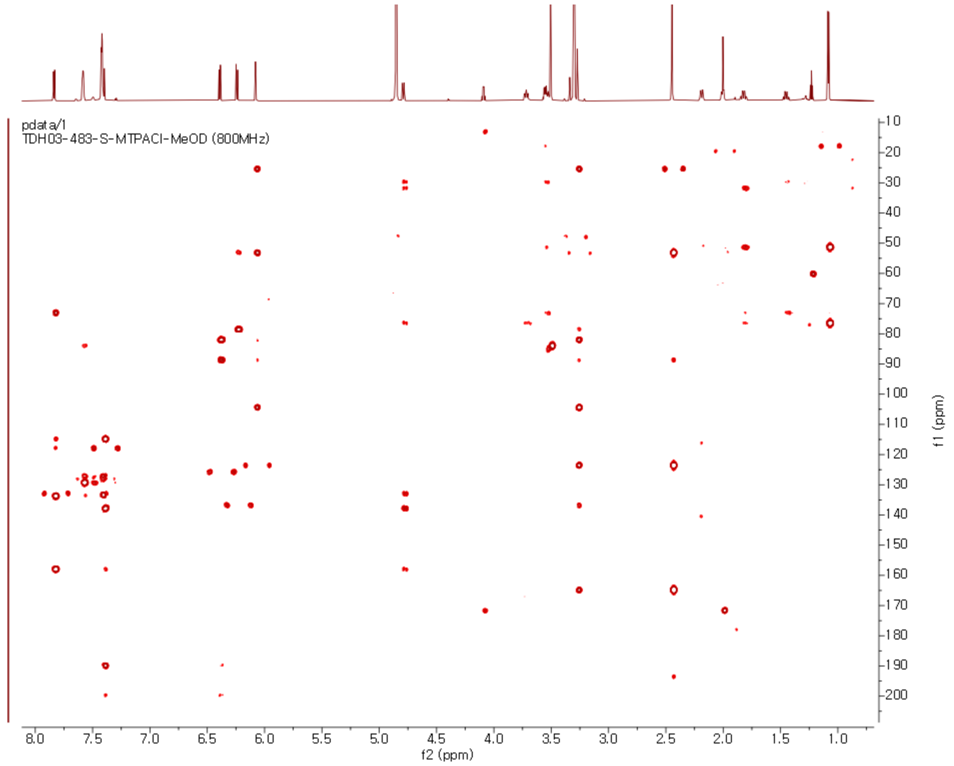

Figure S27. Liquid chromatography profiles of tandocyclinone B (**2**), acetylation product of **2**, and tandocyclinone A (**1**).

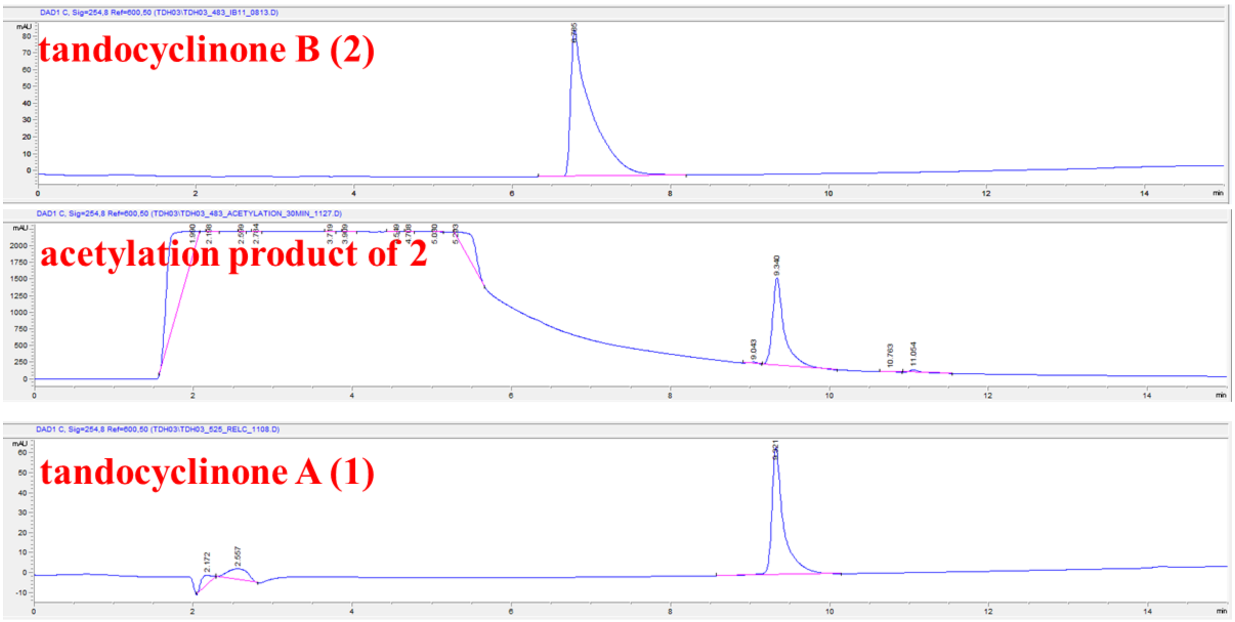

Figure S28. CD spectra for tandocyclinones A (**1**, red), and B (**2**, blue).

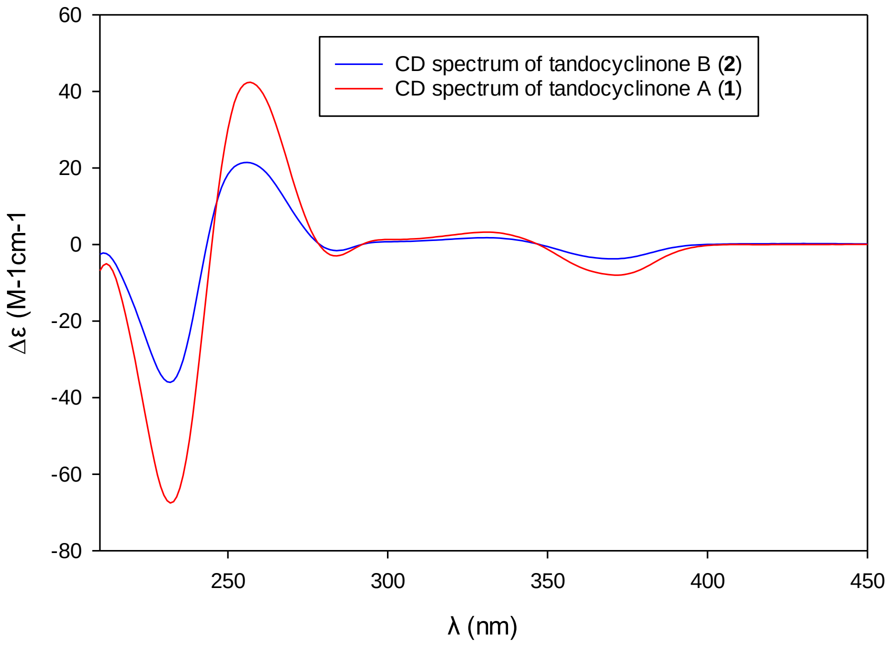

Figure S29. IR spectrum of tandocyclinone A (**1**).

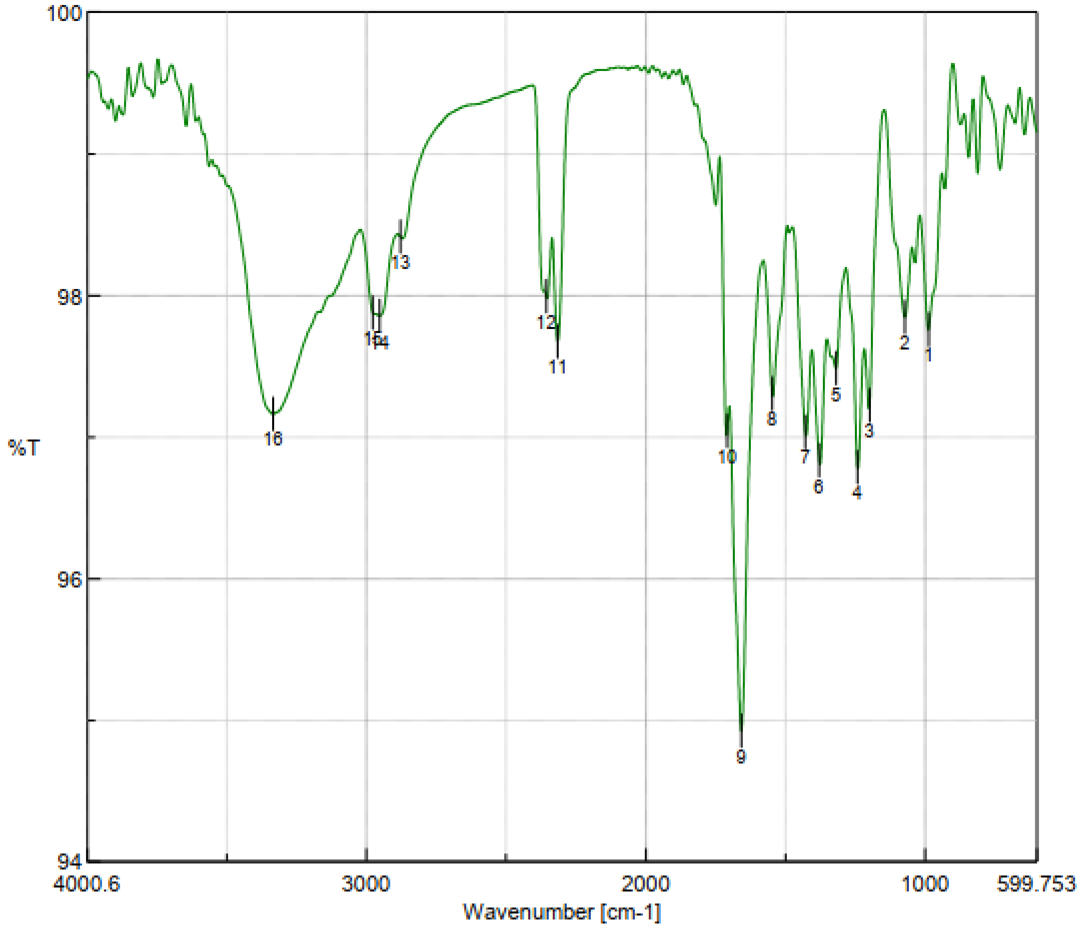

Result of Peak Picking

No.	Position	Intensity	No.	Position	Intensity
1	987.375	97.7578	2	1072.23	97.8433
3	1199.51	97.2256	4	1242.9	96.7881
5	1319.07	97.4874	6	1379.82	96.8371
7	1429.96	97.0349	8	1548.56	97.3074
9	1659.45	94.9246	10	1709.59	97.0423
11	2318.02	97.677	12	2357.55	97.9919
13	2878.24	98.4111	14	2953.45	97.853
15	2978.52	97.8753	16	3334.32	97.163

[Comment]

Sample Name	TDH03_525_2ND
Comment	
User	
Division	
Company	공동기기실

[Measurement Information]

Model Name	FT/IR-4200typeA
Serial Number	B038361018
Light Source	Standard
Detector	TGS
Accumulation	50
Resolution	4 cm-1
Zero Filling	On
Apodization	Cosine
Gain	Auto (2)
Aperture	Auto (7.1 mm)
Scanning Speed	Auto (2 mm/sec)
Filter	Auto (30000 Hz)

Figure S30. IR spectrum of tandocyclinone B (**2**).

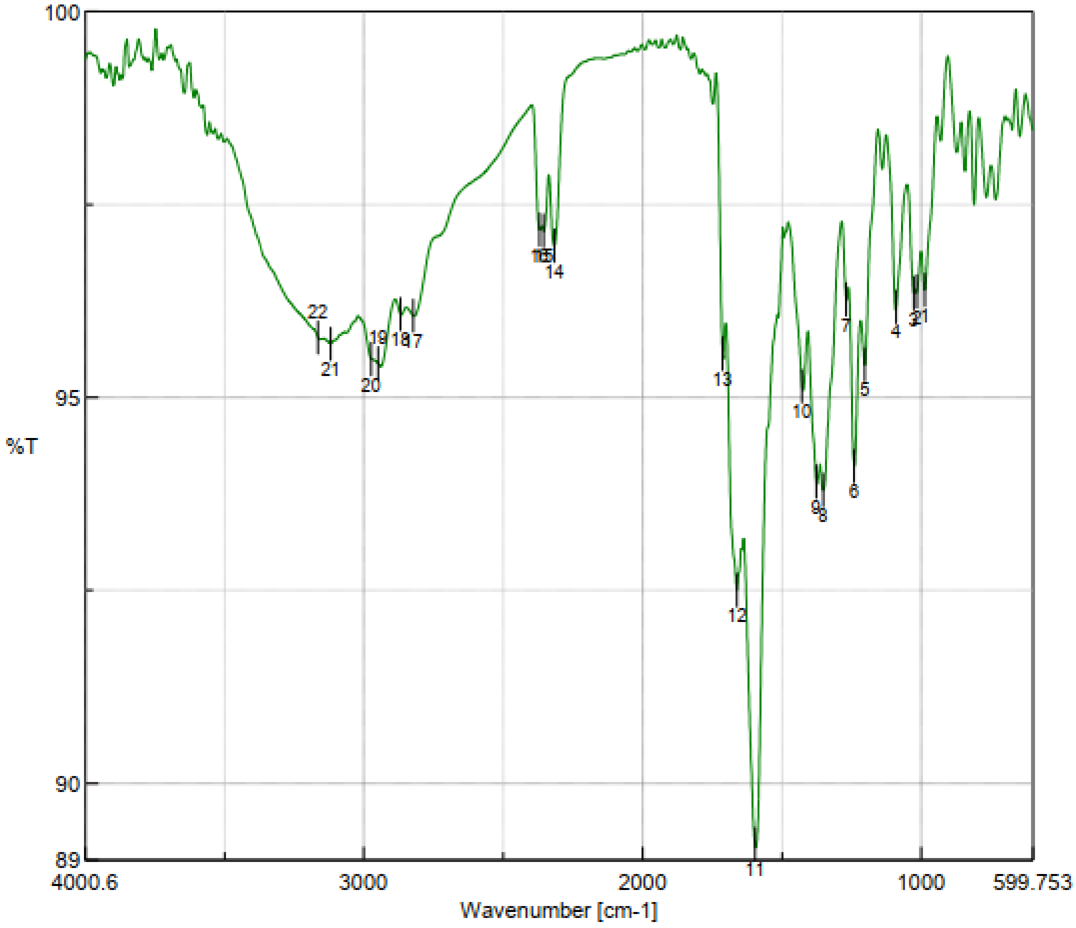

Result of Peak Picking

No.	Position	Intensity	No.	Position	Intensity
1	987.375	96.3899	2	1015.34	96.3713
3	1027.87	96.3545	4	1089.58	96.1707
5	1203.36	95.4207	6	1240.97	94.1056
7	1268.93	96.2687	8	1350.89	93.7919
9	1377.89	93.9009	10	1428.99	95.1378
11	1598.7	89.2046	12	1663.3	92.4979
13	1713.44	95.5586	14	2317.05	96.9642
15	2355.62	97.161	16	2370.09	97.1714
17	2823.28	96.0583	18	2868.59	96.077
19	2948.63	95.4344	20	2973.7	95.4925
21	3119.3	95.6909	22	3164.61	95.773

[Comment]

Sample Name	TDH03_483_3rd
Comment	
User	
Division	
Company	공동기기실

[Measurement Information]

Model Name	FT/IR-4200typeA
Serial Number	B038361018
Light Source	Standard
Detector	TGS
Accumulation	50
Resolution	4 cm-1
Zero Filling	On
Apodization	Cosine
Gain	Auto (2)
Aperture	Auto (7.1 mm)
Scanning Speed	Auto (2 mm/sec)
Filter	Auto (30000 Hz)

Figure S31. HR-ESI-MS data of tandocyclinone A (**1**).

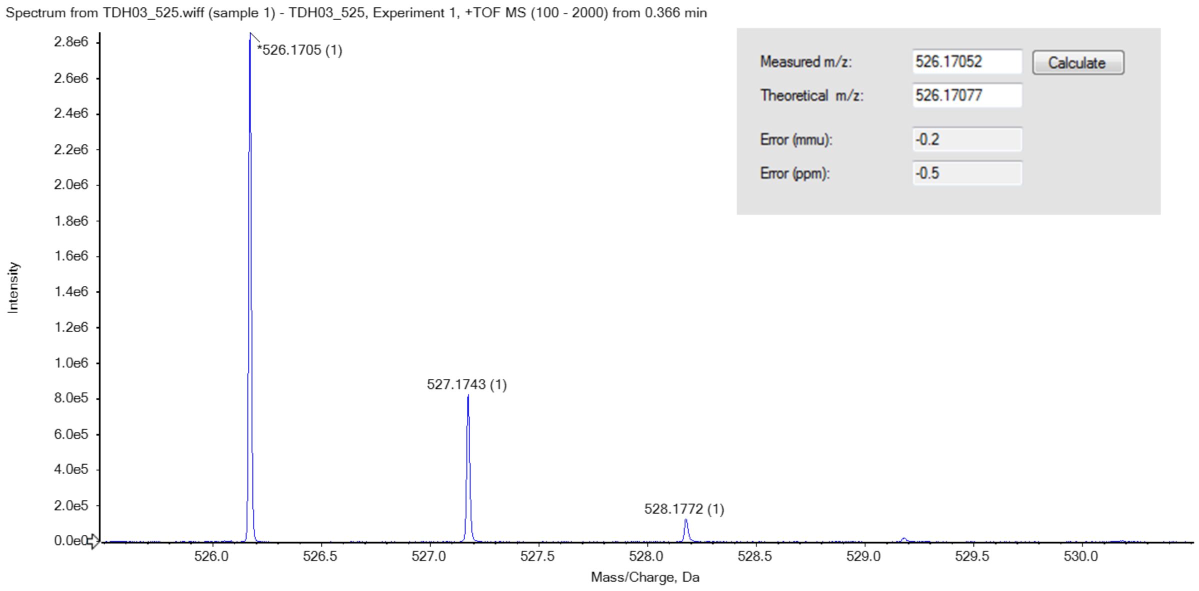

Figure S32. HR-ESI-MS data of tandocyclinone B (**2**).

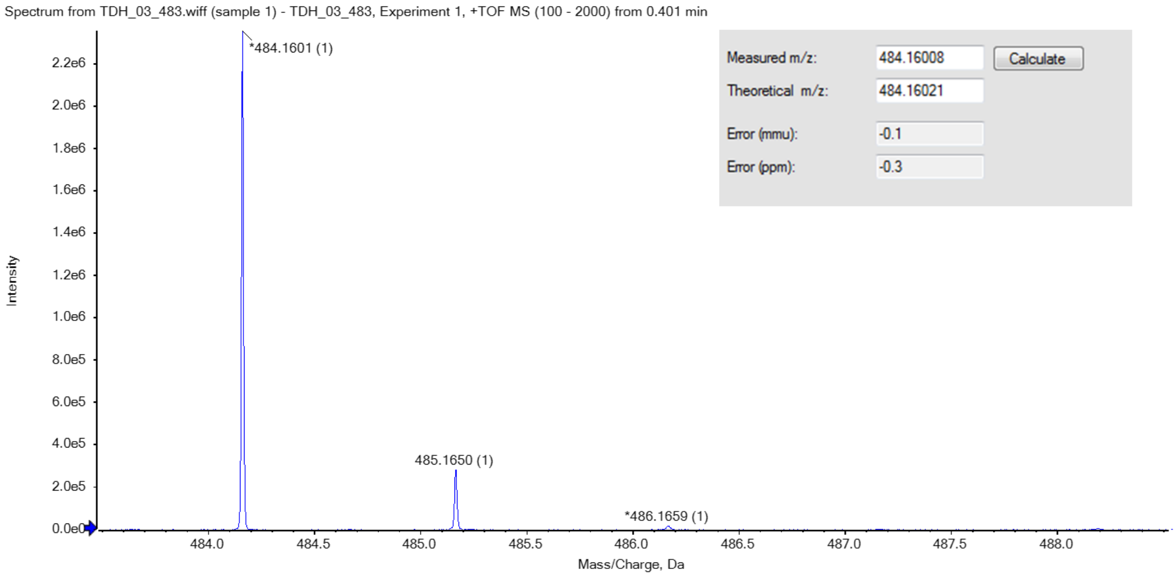

Figure S33. HR-ESI-MS data of (*S*)-MTPA amide (**2a**) of tandocyclinone B (**2**).

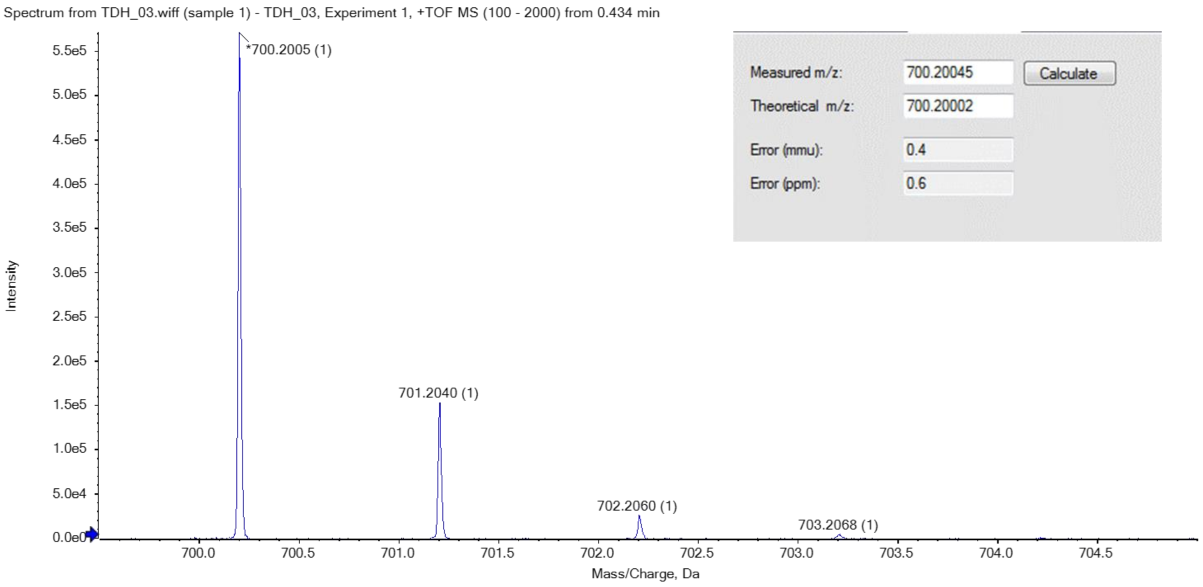

Figure S34. Expanded HR-ESI-MS data of (*R*)-MTPA amide (**2b**) of tandocyclinone B (**2**).

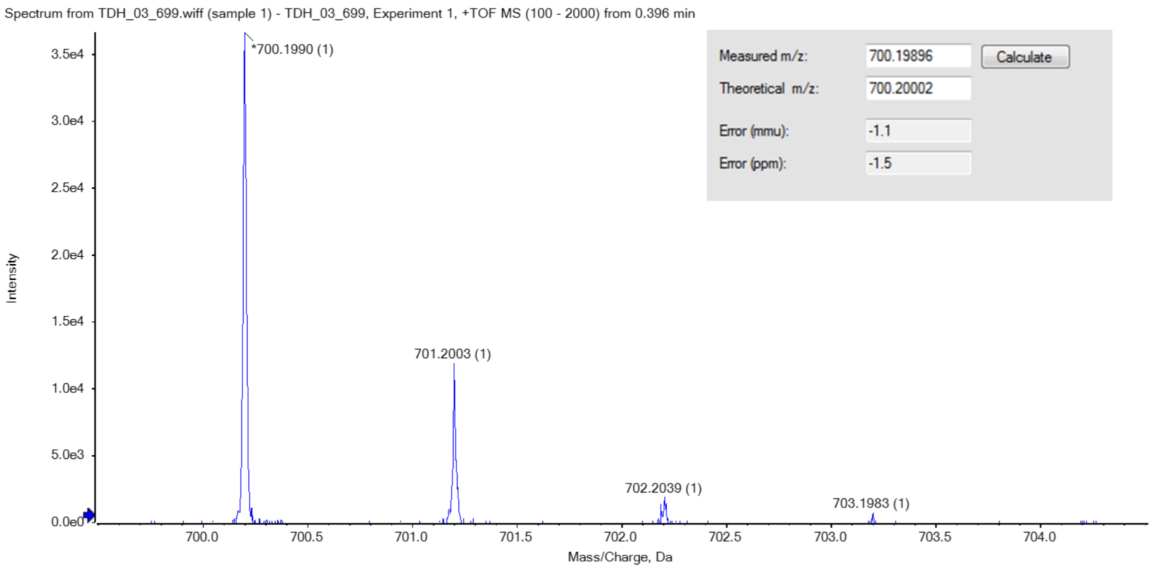

Figure S35. The simulated models of two possible diastereomers **1A** (4*S*,4a*R*,6a*S*,12a*S*,12b*S*,1′*R*,4′*S*,5′*R*) and **1B** (4*R*,4a*S*,6a*R*,12a*R*,12b*R*,1′*R*,4′*S*,5′*R*) of tandocyclinone A (**1**).

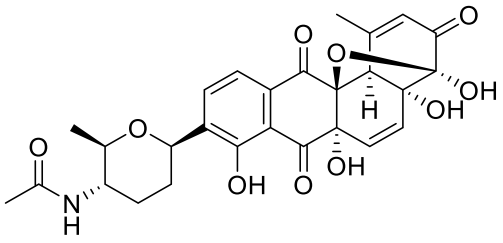

1A

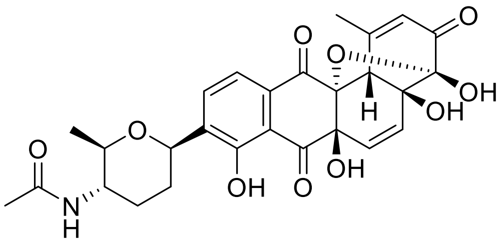

1B

Table S1. Energy analysis for diastereomers **1A** (4*S*,4a*R*,6a*S*,12a*S*,12b*S*,1′*R*,4′*S*,5′*R*) and **1B** (4*R*,4a*S*,6a*R*,12a*R*,12b*R*,1′*R*,4′*S*,5′*R*) of tandocyclinone A (**1**).

Conformer	DFT energy (Hartree)	ΔE（Kcal/mol）	Boltzmann distribution
**1A**			
2	-1851.804159032	0.00	50.06
3	-1851.804122001	0.02	47.64
1	-1851.801399921	1.73	2.30
**1B**			
2	-1851.804093846	0.00	49.62
3	-1851.804047192	0.03	47.71
1	-1851.801185696	1.82	2.67

Table S2. ECD calculation of conformer 1 of diastereomer **1A** .

Total energy = -1851.801399921
Kinetic energy = 1843.391596526
Potential energy = -3695.192996447

Parameters of Level DFT
DFT settings (Functional B3LYP / Gridsize M3)
Geometry optimization options (Energy 10^{-6} Hartree, Gradient norm │dE / dxyz│= 10^{-3} Hartree/Bohr)

Energy minimized coordinates of conformer 1 of diastereomer **1A** at the basis set def2-TZVP for all atoms(Å).

Atom	Atomic coordinates			Atom	Atomic coordinates		
C	2.64546617	-5.74996681	17.41241928	O	19.12001953	-0.85561729	0.68881342
C	3.47145224	-7.56104483	15.28761035	H	14.91751076	2.45560120	1.80821877
C	5.54282416	-6.35629565	13.65439101	O	14.86052367	-2.34584481	5.84000359
C	4.70240820	-3.71400153	12.76455884	C	-1.72688777	-7.46713340	18.14446991
O	4.06277854	-2.19463325	14.89386512	C	-3.43689638	-8.70866522	20.10326974
C	1.95321153	-3.12960916	16.30783704	O	-2.43866607	-7.10632406	15.96740110
C	1.37137290	-1.14797706	18.31123511	H	4.25364373	-5.44326575	18.68699421
C	6.73938247	-2.35453695	11.28691604	H	1.81666847	-8.00607335	14.12628321
C	7.23620480	-3.08625053	8.76479655	H	4.14172567	-9.34017574	16.11503949
C	9.16183967	-1.86747054	7.35740471	H	5.96330482	-7.54734164	12.01507318
C	10.57827828	0.10035763	8.50876893	H	7.29598719	-6.14221302	14.74524415
C	10.04857815	0.83844075	10.96991990	H	3.02837934	-3.93409665	11.54455888
C	8.14701598	-0.39656190	12.33642962	H	0.32605384	-3.36307261	15.04013228
N	0.62977833	-6.81904079	18.99047180	H	-0.27265418	-1.70083009	19.43635428
O	5.81183815	-4.93932439	7.77156605	H	2.99168757	-0.89340861	19.57916156
C	12.64290998	1.38694457	7.10131783	H	0.96999566	0.66935767	17.40863159
C	9.57223077	-2.55430470	4.71332720	H	11.13749255	2.35954029	11.80758181
O	13.50620684	3.41961026	7.75448286	H	7.72137348	0.17326921	14.25794020
C	13.68291743	-0.09969043	4.83093040	H	1.05775915	-7.20587421	20.80826577
C	15.76613202	1.19385026	3.20336741	H	6.37809629	-5.17406731	5.99684488
C	16.83364148	-1.26114360	2.00016929	H	15.55884897	-3.52181879	-1.30006366
C	14.86514419	-2.50183146	0.34310068	H	10.99909134	-3.33504379	-0.28696992
C	12.40368585	-2.39025518	0.87506648	H	20.86992829	2.07412461	7.29828090
C	11.45680511	-0.96649476	3.14680558	H	8.82758626	0.84256222	1.22666888
O	8.31716446	-4.21001737	3.62905937	H	18.95734926	-5.68754888	5.45686503
C	17.77649651	2.54329738	4.74187793	H	16.14836377	6.24423440	4.92153421
C	19.39392358	1.18394831	6.18321129	H	19.41857495	6.14756575	5.70693324
C	19.32035529	-1.55856017	6.12684074	H	18.43268162	5.84130642	2.50817267
C	17.21967227	-2.75901528	4.48112040	H	19.91639179	-2.50148855	0.49336089
O	10.11859107	1.30085152	2.45036335	H	-5.29911596	-7.81743811	20.01235939
O	17.71769849	-5.27254119	4.13097932	H	-3.68227955	-10.70468623	19.60893773
C	17.96180683	5.35621893	4.47217506	H	-2.72213186	-8.59150362	22.04198958
O	20.75920746	-2.99064643	7.25348656				

Table S3. ECD calculation of conformer 2 of diastereomer **1A** .

Total energy = -1851.804159032
Kinetic energy = 1843.392954138
Potential energy = -3695.197113171

Parameters of Level DFT
DFT settings (Functional B3LYP / Gridsize M3)
Geometry optimization options (Energy 10^{-6} Hartree, Gradient norm │dE / dxyz│= 10^{-3} Hartree/Bohr)

Energy minimized coordinates of conformer 2 of diastereomer **1A** at the basis set def2-TZVP for all atoms(Å).

Atom	Atomic coordinates			Atom	Atomic coordinates		
C	1.80158061	-5.90025287	16.42924930	O	19.39274055	-0.88440481	0.99040331
C	2.62367277	-7.56094311	14.19208633	H	15.33579740	2.61576026	2.06015588
C	4.83874392	-6.36221794	12.75759571	O	14.73961362	-2.39442336	5.78275973
C	4.19355163	-3.61344762	12.05020056	C	-0.56904226	-7.35841348	20.22001784
O	3.53910771	-2.22288346	14.25795780	C	-2.97743790	-8.65067033	21.12413605
C	1.35059900	-3.14971917	15.54248284	O	1.11351547	-6.74590951	21.68603427
C	0.81656546	-1.31424334	17.69226110	H	3.28721950	-5.86732720	17.86886870
C	6.37048256	-2.27192425	10.76798277	H	1.01584815	-7.79833472	12.88894558
C	6.94875725	-2.85283015	8.22515141	H	3.12561116	-9.45171865	14.87464636
C	9.01092673	-1.66030279	7.00110743	H	5.27677151	-7.44195206	11.04775966
C	10.47326969	0.13709896	8.35528728	H	6.53532633	-6.34526971	13.95017222
C	9.86132202	0.73625781	10.83495942	H	2.56825530	-3.63823105	10.74309952
C	7.83015617	-0.47812323	12.02159445	H	-0.25641362	-3.14939479	14.20332906
N	-0.42154472	-6.95843732	17.66210723	H	-0.91046839	-1.84555028	18.69558289
O	5.47066336	-4.54198259	7.03267388	H	2.37860450	-1.31309060	19.04942210
C	12.67592346	1.39095972	7.13825506	H	0.58829152	0.59994765	16.94177742
C	9.52538594	-2.19744694	4.34017754	H	10.98862705	2.12976757	11.82903617
O	13.59823463	3.33663917	7.95347623	H	7.34458006	-0.02170680	13.95918186
C	13.76477043	-0.02360826	4.84612836	H	-1.85393304	-7.56830156	16.55522033
C	16.01856675	1.22633404	3.42427836	H	6.11617008	-4.68473593	5.27440479
C	17.01015847	-1.21902592	2.13998456	H	15.80803904	-3.20525921	-1.35824460
C	15.07662445	-2.23731675	0.29987626	H	11.21430029	-2.78655208	-0.60472082
C	12.59779627	-1.99962857	0.69240749	H	20.89549461	1.55121213	7.86234464
C	11.59824334	-0.64878435	2.98633843	H	9.21939015	1.43230738	1.01732664
O	8.22733025	-3.68812169	3.08039459	H	18.64992762	-5.95828336	5.44229589
C	18.00423495	2.35763905	5.15749745	H	16.58868853	6.13709986	5.46121349
C	19.44332779	0.82016852	6.60873716	H	19.79074316	5.79149324	6.43528429
C	19.21231843	-1.90514214	6.38157052	H	18.99444317	5.72890642	3.17032830
C	17.15200359	-2.87667051	4.54424522	H	20.10465653	-2.56253231	0.74940407
O	10.44414171	1.73501040	2.35230435	H	-2.49690620	-10.53101553	21.84484433
O	17.52146720	-5.39219118	4.07345524	H	-3.75892967	-7.56840437	22.70227220
C	18.37162472	5.16412289	5.06972403	H	-4.42491200	-8.85175893	19.65831456
O	20.49069195	-3.48666167	7.50092462				

Table S4. ECD calculation of conformer 3 of diastereomer **1A** .

Total energy = -1851.804122001
Kinetic energy = 1843.388272313
Potential energy = -3695.192394314

Parameters of Level DFT
DFT settings (Functional B3LYP / Gridsize M3)
Geometry optimization options (Energy 10^{-6} Hartree, Gradient norm │dE / dxyz│= 10^{-3} Hartree/Bohr)

Energy minimized coordinates of conformer 3 of diastereomer **1A** at the basis set def2-TZVP for all atoms(Å).

Atom	Atomic coordinates			Atom	Atomic coordinates		
C	1.81072613	-5.99701019	16.46670405	O	19.43957612	-0.77180902	1.01489773
C	2.78490026	-7.73243152	14.34928772	H	15.22690589	2.56718358	1.99754729
C	4.99183691	-6.51044287	12.92062711	O	14.84243256	-2.38167327	5.82911144
C	4.25188864	-3.83310036	12.05184804	C	-0.67234888	-7.30865725	20.23994076
O	3.47036330	-2.36339232	14.16575474	C	-3.07675451	-8.62013778	21.12704100
C	1.27658042	-3.31796010	15.42075469	O	0.90082438	-6.50366493	21.73404510
C	0.58324240	-1.39696087	17.44776236	H	3.23758614	-5.82390025	17.95449784
C	6.40399741	-2.46043444	10.76119403	H	1.23472807	-8.11095911	13.01088929
C	7.04763625	-3.10731115	8.25024997	H	3.34695204	-9.55975728	15.14830463
C	9.07327787	-1.86813088	7.01156837	H	5.53030058	-7.65642662	11.28367479
C	10.44275080	0.03074038	8.32365528	H	6.64782106	-6.35728332	14.15976450
C	9.77417463	0.68278811	10.77556105	H	2.66983577	-3.99712136	10.70328699
C	7.77295520	-0.56932401	11.97359729	H	-0.27710991	-3.46030563	14.02757300
N	-0.41141391	-7.09215307	17.66962611	H	-1.15660341	-1.95073706	18.41595277
O	5.66193728	-4.90038878	7.09938225	H	2.09017909	-1.25414858	18.85811553
C	12.60564383	1.33891103	7.09240103	H	0.30221852	0.46233297	16.58530552
C	9.63598339	-2.45945756	4.37156962	H	10.83152025	2.15160397	11.73740985
O	13.44750002	3.33545361	7.87109402	H	7.23761120	-0.06457417	13.88600078
C	13.76534014	-0.07894668	4.83661795	H	-1.75691457	-7.84831475	16.54442537
C	15.96621949	1.23902423	3.39330282	H	6.33766525	-5.07483915	5.35543432
C	17.07014252	-1.18696386	2.16518407	H	15.97092315	-3.29637292	-1.29472257
C	15.19094664	-2.32916047	0.34150864	H	11.36014971	-3.06995150	-0.56094505
C	12.70265603	-2.19514438	0.72295299	H	20.81169196	1.87599569	7.83253775
C	11.63594227	-0.84407503	2.98611941	H	9.17242057	1.08358761	0.96413902
O	8.42570250	-4.05202466	3.15008780	H	18.90668208	-5.77662667	5.57275595
C	17.89474619	2.49471919	5.10614669	H	16.31386695	6.21426534	5.32591507
C	19.39673431	1.05423218	6.59271820	H	19.52540142	6.03044648	6.31195951
C	19.28720443	-1.68280836	6.42468491	H	18.74063486	5.86282045	3.04788948
C	17.27709414	-2.78406017	4.60526412	H	20.22527014	-2.42165887	0.81055768
O	10.37358921	1.46922696	2.29913441	H	-4.29369263	-9.28206699	19.58952129
O	17.75818212	-5.29047967	4.19000018	H	-2.55351845	-10.23232638	22.31272136
C	18.13859809	5.31214790	4.95803057	H	-4.15146361	-7.31006618	22.31434281
O	20.63130157	-3.18130720	7.58085426				

Table S5. ECD calculation of conformer 1 of diastereomer **1B** .

Total energy = -1851.801185696
Kinetic energy = 1843.391653858
Potential energy = -3695.192839555

Parameters of Level DFT
DFT settings (Functional B3LYP / Gridsize M3)
Geometry optimization options (Energy 10^{-6} Hartree, Gradient norm │dE / dxyz│= 10^{-3} Hartree/Bohr)

Energy minimized coordinates of conformer 1 of diastereomer **1B** at the basis set def2-TZVP for all atoms(Å).

Atom	Atomic coordinates			Atom	Atomic coordinates		
C	-28.64492164	-9.00466975	-4.18394303	O	-5.36332591	-4.77617303	-10.41437238
C	-26.94919431	-7.80434855	-2.14544195	H	-9.53381180	-1.30130379	-9.80463582
C	-24.60928967	-6.57902258	-3.34507906	O	-11.68478325	-7.17021164	-9.31575225
C	-23.26982773	-8.48210420	-5.09589401	C	-30.83819679	-12.22029096	-1.45450240
O	-25.00458610	-9.40502704	-6.93789076	C	-33.40938769	-13.04233696	-0.44685762
C	-27.07699316	-10.79538998	-5.88740653	O	-28.88063111	-13.26959606	-0.79033696
C	-28.55529844	-11.86313536	-8.11229093	H	-29.36497864	-7.50097399	-5.41934845
C	-21.04793540	-7.32148815	-6.47543362	H	-26.37774755	-9.28316562	-0.81555114
C	-18.76331591	-6.89534756	-5.15610837	H	-28.03753251	-6.39890489	-1.07759975
C	-16.66237166	-5.80078162	-6.40655386	H	-23.29680421	-5.92940030	-1.88324781
C	-16.89008109	-5.11577511	-8.99122371	H	-25.15655869	-4.93100214	-4.48340339
C	-19.15100975	-5.50522802	-10.26336363	H	-22.58191119	-10.07028089	-3.93730443
C	-21.19665693	-6.61913625	-9.00561485	H	-26.34133843	-12.34023253	-4.71167523
N	-30.88891267	-10.23242010	-3.10850035	H	-30.13623445	-13.02202263	-7.45468310
O	-18.67729625	-7.55094335	-2.70417081	H	-29.29805181	-10.33353082	-9.29841627
C	-14.71451302	-3.98639585	-10.36719506	H	-27.31821895	-13.03953110	-9.28003086
C	-14.34583903	-5.27766896	-4.99543237	H	-19.28741249	-4.96372309	-12.23563912
O	-14.96627561	-2.83732048	-12.34679182	H	-22.94840936	-6.98164787	-10.00547314
C	-12.11767023	-4.48020758	-9.16050508	H	-32.58583540	-9.46996247	-3.52833000
C	-9.77056389	-3.24935857	-10.44146864	H	-16.95650037	-7.12774439	-2.08361849
C	-7.76632542	-5.06041547	-9.29303366	H	-5.82590831	-5.05794528	-5.56873851
C	-7.65611742	-4.76086061	-6.45451610	H	-9.62524198	-4.04163308	-3.04222506
C	-9.69782462	-4.19590634	-5.08829713	H	-9.51456663	-5.88882493	-16.48765273
C	-12.22286448	-3.77776188	-6.32657288	H	-12.85534757	-0.60267465	-4.48650808
O	-14.10704462	-5.79636833	-2.72280940	H	-8.12149613	-10.98767392	-10.20599457
C	-9.78638523	-3.41431816	-13.30513778	H	-8.28439606	0.18474933	-14.34847416
C	-9.52800499	-5.68913900	-14.44446055	H	-10.03575224	-1.31776022	-16.79231680
C	-9.06707642	-7.93982604	-12.94300456	H	-11.62645776	0.08390666	-14.18012114
C	-9.05796486	-7.57475029	-10.03919458	H	-4.40122550	-6.28586081	-9.99378822
O	-12.98914717	-1.16591508	-6.22991373	H	-33.58276883	-12.43841097	1.52686711
O	-8.04175949	-9.66220558	-8.90088239	H	-35.01176920	-12.27983829	-1.51216978
C	-9.95159417	-0.98600679	-14.75381105	H	-33.49752208	-15.10631751	-0.47177193
O	-8.68945819	-10.07825574	-13.76359474				

Table S6. ECD calculation of conformer 2 of diastereomer **2B** .

Total energy = -1851.804093846
Kinetic energy = 1843.399002355
Potential energy = -3695.203096201

Parameters of Level DFT
DFT settings (Functional B3LYP / Gridsize M3)
Geometry optimization options (Energy 10^{-6} Hartree, Gradient norm │dE / dxyz│= 10^{-3} Hartree/Bohr)

Energy minimized coordinates of conformer 2 of diastereomer **2B** at the basis set def2-TZVP for all atoms(Å).

Atom	Atomic coordinates			Atom	Atomic coordinates		
C	-28.11283176	-9.89002125	-3.45022128	O	-5.40014076	-4.45686157	-10.78735123
C	-26.34941553	-8.65608015	-1.49940118	H	-9.66249095	-1.16190382	-9.87661730
C	-24.16427341	-7.25871466	-2.79581679	O	-11.56252778	-7.12037411	-9.43303907
C	-22.83213990	-9.03371647	-4.68046602	C	-32.59455477	-11.09212798	-2.67452198
O	-24.61356679	-9.99252075	-6.45362007	C	-34.33862207	-12.71369778	-1.05712896
C	-26.57087217	-11.49390483	-5.34936184	O	-33.41563716	-9.62458046	-4.26375638
C	-28.13898513	-12.53567370	-7.52465542	H	-29.11749269	-8.43047935	-4.51719941
C	-20.74281999	-7.72866056	-6.13468888	H	-25.56991809	-10.13206478	-0.25365962
C	-18.41228194	-7.24554169	-4.92235336	H	-27.43695342	-7.36839096	-0.29429980
C	-16.42669725	-6.02819667	-6.24436986	H	-22.80315366	-6.56928983	-1.39863366
C	-16.81759641	-5.28158538	-8.79157805	H	-24.88612604	-5.62122753	-3.84492037
C	-19.12597303	-5.72571977	-9.95653598	H	-22.00208573	-10.61956971	-3.61126952
C	-21.05652544	-6.95862301	-8.63001271	H	-25.68506558	-13.07335919	-4.30323955
N	-30.06128849	-11.38235394	-2.20022006	H	-29.61716236	-13.79797993	-6.82160271
O	-18.16796898	-7.97067146	-2.49887782	H	-29.04379798	-10.99481374	-8.56769386
C	-14.76064099	-4.02977542	-10.24388560	H	-26.92518408	-13.59293643	-8.82415752
C	-14.06140961	-5.45045481	-4.93908621	H	-19.38872239	-5.13394312	-11.90138683
O	-15.15988279	-2.83792444	-12.17268444	H	-22.84375433	-7.36224913	-9.54839592
C	-12.08756156	-4.45352790	-9.18554911	H	-29.52010059	-12.58658613	-0.81952701
C	-9.85811517	-3.10163208	-10.55169686	H	-16.43551727	-7.48715286	-1.95770549
C	-7.72990868	-4.86063916	-9.55421539	H	-5.60048218	-4.86823519	-5.93504051
C	-7.48449818	-4.62444766	-6.71805212	H	-9.29878879	-4.06442746	-3.19216807
C	-9.47262177	-4.17360009	-5.23525878	H	-9.81571004	-5.58230275	-16.66979474
C	-12.07271076	-3.82772272	-6.33173261	H	-12.72303484	-0.72544176	-4.37686068
O	-13.68410668	-6.02476169	-2.69860342	H	-7.90145947	-10.77000914	-10.60787872
C	-10.01534833	-3.19768693	-13.41428155	H	-8.70958315	0.48530267	-14.42778445
C	-9.73230797	-5.43305282	-14.62398874	H	-10.50890763	-1.02785203	-16.82974101
C	-9.10852021	-7.69915545	-13.20736271	H	-12.03325594	0.24129096	-14.11236343
C	-8.96315463	-7.40449370	-10.29917506	H	-4.36718799	-5.94440425	-10.46871010
O	-12.93873453	-1.25458577	-6.12262039	H	-35.70750745	-13.64867811	-2.29160693
O	-7.81275418	-9.47848071	-9.26932288	H	-33.35126460	-14.14434064	0.06576122
C	-10.34462957	-0.74372519	-14.78895868	H	-35.40319050	-11.48243362	0.22239693
O	-8.69071360	-9.80059085	-14.10071987				

Table S7. ECD calculation of conformer 3 of diastereomer **2B** .

Total energy = -1851.804047192
Kinetic energy = 1843.395474134
Potential energy = -3695.199521326

Parameters of Level DFT
DFT settings (Functional B3LYP / Gridsize M3)
Geometry optimization options (Energy 10^{-6} Hartree, Gradient norm │dE / dxyz│= 10^{-3} Hartree/Bohr)

Energy minimized coordinates of conformer 3 of diastereomer **2B** at the basis set def2-TZVP for all atoms(Å).

Atom	Atomic coordinates			Atom	Atomic coordinates		
C	-28.100556	-9.82102081	-3.3555951	O	-5.39953377	-4.38161795	-10.7305696
C	-26.3768281	-8.45635353	-1.45683878	H	-9.75002334	-1.13799783	-10.0929008
C	-24.1981255	-7.10066249	-2.80658394	O	-11.5192924	-7.09942382	-9.29266702
C	-22.81848	-8.95661157	-4.57550946	C	-32.5705692	-11.0847199	-2.61910127
O	-24.5652825	-10.0443811	-6.30820988	C	-34.3202823	-12.6658432	-0.96809061
C	-26.5119176	-11.5101035	-5.14007896	O	-33.3787817	-9.7630261	-4.33809979
C	-28.036667	-12.703171	-7.26807855	H	-29.1064591	-8.43759433	-4.51882072
C	-20.7364473	-7.70083402	-6.0824643	H	-25.5900434	-9.85023589	-0.12379243
C	-18.4313394	-7.09746842	-4.87526413	H	-27.4952292	-7.12022232	-0.33581179
C	-16.4540765	-5.92132542	-6.24586184	H	-22.8631847	-6.313298	-1.4360442
C	-16.8259132	-5.34179394	-8.83919503	H	-24.9325538	-5.53550707	-3.95274951
C	-19.1098041	-5.90410706	-10.0015983	H	-21.9730863	-10.4653116	-3.41013532
C	-21.0331614	-7.09135312	-8.62389604	H	-25.6148055	-13.0107613	-3.99241248
N	-30.0480696	-11.2605636	-2.04492051	H	-29.499859	-13.9502332	-6.50888919
O	-18.2034181	-7.6694661	-2.40938214	H	-28.9581508	-11.2422298	-8.40699323
C	-14.7743478	-4.1424489	-10.3426394	H	-26.7890355	-13.8107857	-8.49145579
C	-14.1198288	-5.21121871	-4.94980195	H	-19.3587294	-5.4391002	-11.9823603
O	-15.1693594	-3.08907175	-12.3511881	H	-22.800448	-7.58628984	-9.53591756
C	-12.108332	-4.43608624	-9.22322209	H	-29.5166213	-12.3532303	-0.57101146
C	-9.89058335	-3.1213287	-10.6431842	H	-16.4880664	-7.11805697	-1.87859746
C	-7.73766686	-4.76119201	-9.5056709	H	-5.66257236	-4.48584718	-5.86510717
C	-7.53992957	-4.33808344	-6.68741622	H	-9.41967809	-3.59701076	-3.22976293
C	-9.55991355	-3.8407443	-5.26369552	H	-9.69924499	-5.98944348	-16.5874554
C	-12.1506589	-3.62960129	-6.41536021	H	-12.903916	-0.4240705	-4.67359153
O	-13.7594033	-5.64107553	-2.67448278	H	-7.75656071	-10.7280419	-10.1776056
C	-10.0027943	-3.40555607	-13.4959969	H	-8.76543484	0.23275426	-14.7309008
C	-9.64966262	-5.70673948	-14.5546585	H	-10.4979548	-1.47365986	-17.0513098
C	-8.99352154	-7.86086566	-12.986317	H	-12.0869005	-0.06615022	-14.4412273
C	-8.90079614	-7.37647108	-10.1017756	H	-4.33757384	-5.8208639	-10.3032239
O	-13.080928	-1.07051379	-6.38401648	H	-35.7402026	-11.4207154	-0.12458125
O	-7.71932455	-9.35166304	-8.92389075	H	-35.3265249	-14.0191443	-2.16689247
C	-10.3679208	-1.05404891	-15.0314383	H	-33.350397	-13.6993263	0.53961178
O	-8.51094838	-10.00418	-13.7363571				

Figure S36. The proposed polyketide cyclization patterns of (a) tandocyclinones and (b) angucyclinones.

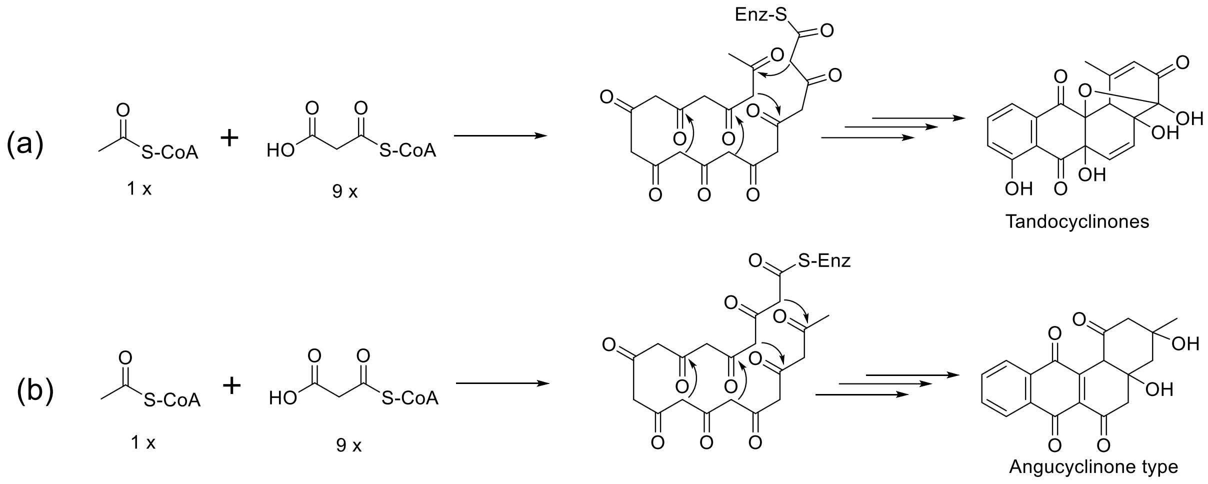

Proposed biosynthetic pathways of tandocyclinones A and B (1 and 2)

The core structure of **1** and **2** could be biosynthesized by a common type II PKS. A set of type II polyketide synthase (PKS) catalyzes the condensation of an acetyl-CoA starter unit with 9 malonyl-CoA extender units to synthesize the linear backbone (i) via Claisen condensation. Then, intramolecular cyclization and aromatization could be achieved to afford the benz[*a*]anthracene ring (ii). This intermediate may have undergone a series of oxidation reactions at C-3, C-6a, and C-12 to yield intermediate iii. Subsequent nucleophilic attack of 12a-OH on carbonyl C-4 to give rise to the hemiacetal group at C-4 and ether bridged C-4–O–C-12 linkage (iv). The double bond between C-5 and C-6 is likely formed under reduction on carbonyl C-6 and later a loss of H_2O to form v. As in the discussion in the manuscript, a known pyranonaphthoquinone BE-54238A with the involvement of a 4′-amino-4′-deoxy-α-D-amicetose (α-d-forasmine) as the deoxyaminosugar biosynthetic precursor during *C*-glycosylation was also isolated from the same bacterial strain. Thus, intermediate v is proposed to be C-glycosylated by a glycotransferase at C-9 to generate tandocyclinone B (**2**). Tandocyclinone A (**1**) is rationally formed by an acetylation enzyme.

Scheme S1. Proposed biosynthetic pathways of tandocyclinones A and B (**1** and **2**)

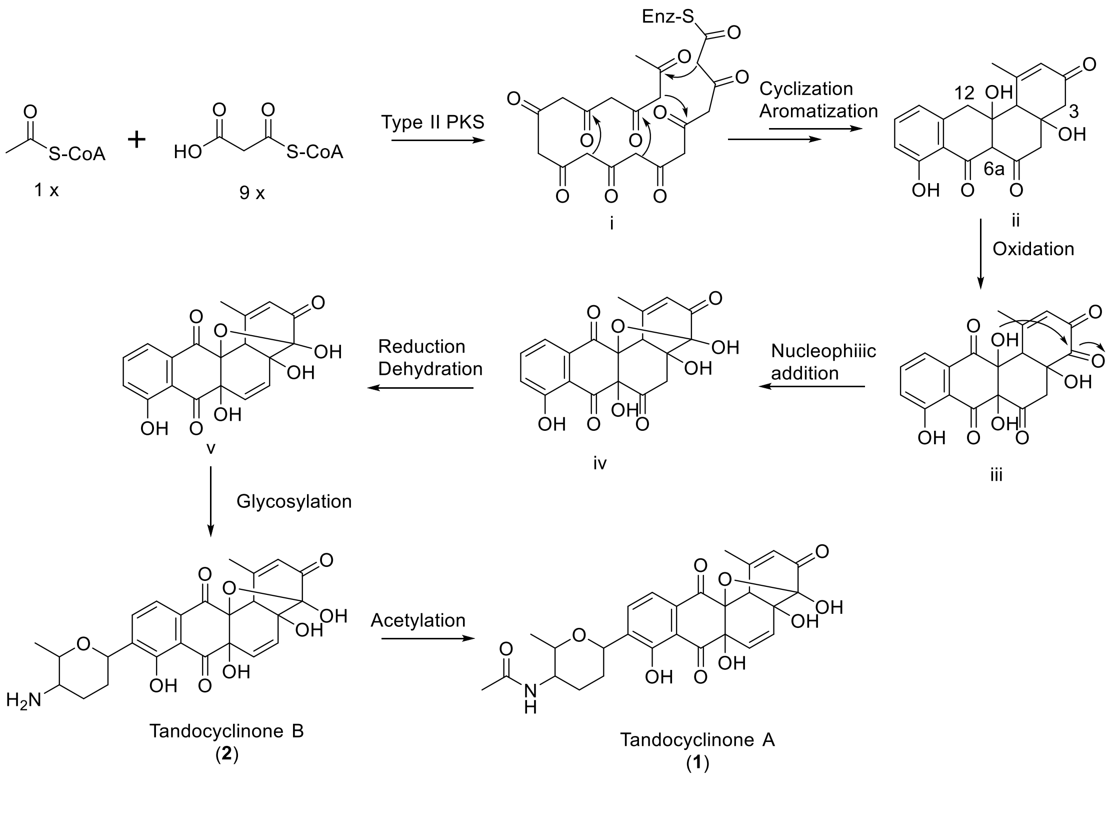

Figure S37. ^{1}H NMR (600 MHz, DMSO-d_6) and UV-vis (inset) spectra of BE-54238A (mass 397) from reference.

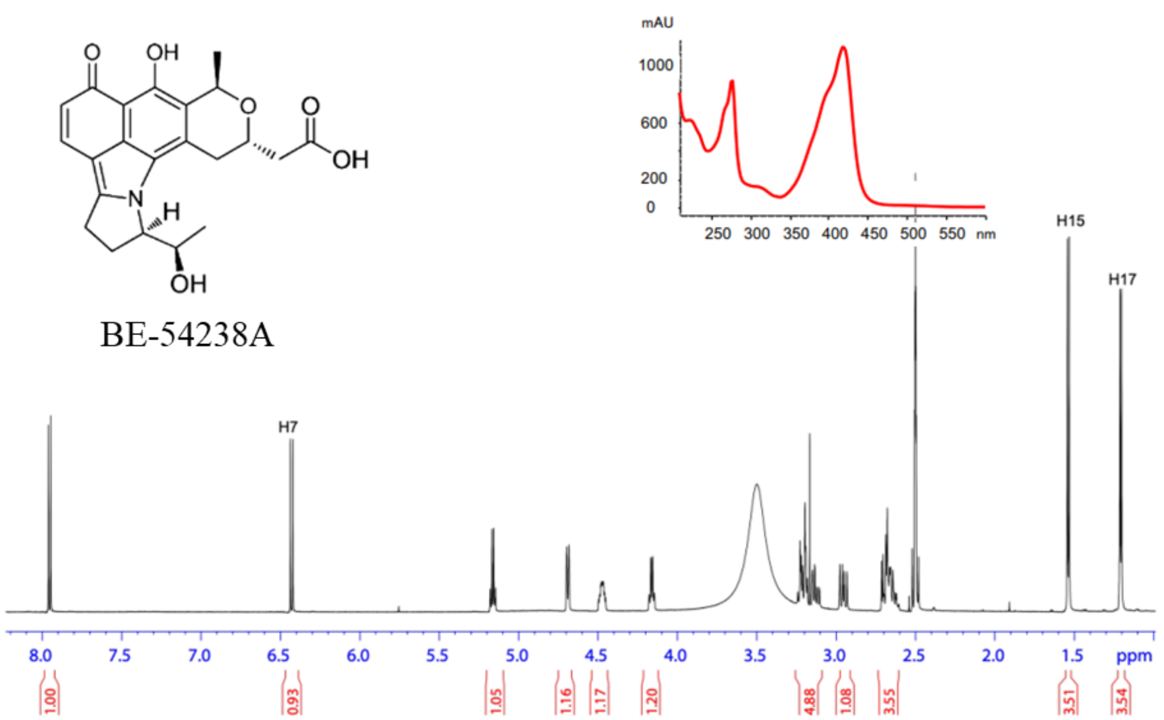

Figure S38. ^{1}H NMR (800 MHz, DMSO-d_6), UV-vis (inset) and MS (positive mode, inset) spectra of TDH03.397.

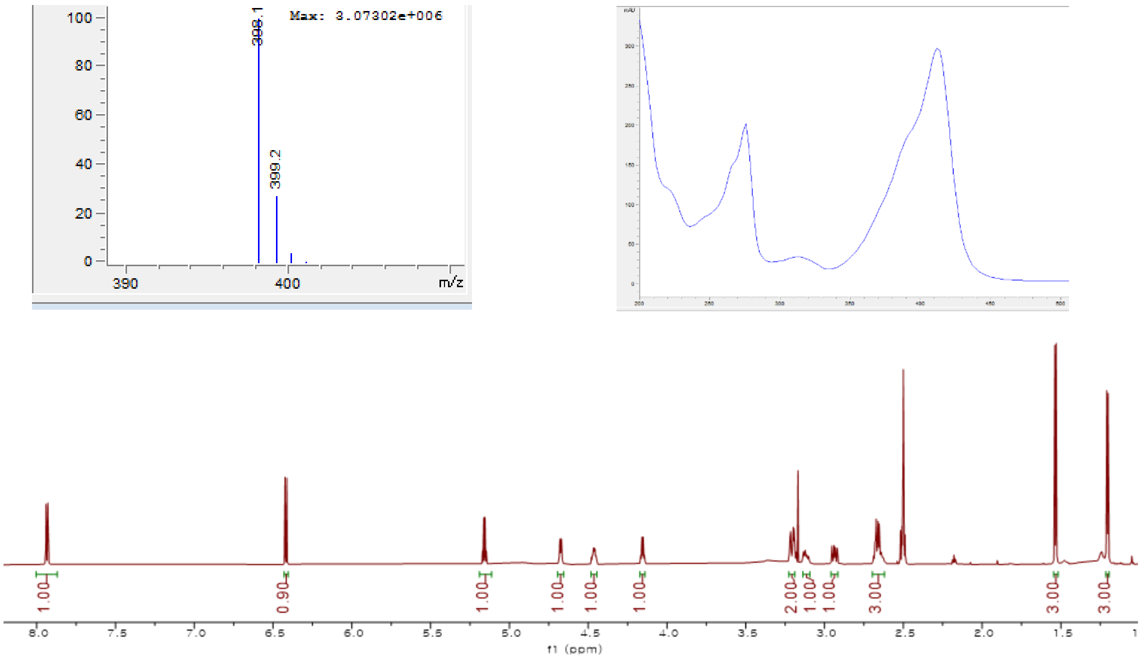

Supplement: Supplementary file 1 [file marinedrugs-21-00500-s001.zip › marinedrugs-2591180-supplementary.pdf]
